# LSD1 Regulates Neurogenesis in Human Neural Stem Cells Through the Repression of Human-Enriched Extracellular Matrix and Cell Adhesion Genes

**DOI:** 10.1093/stmcls/sxad088

**Published:** 2023-12-28

**Authors:** Asha S Channakkar, Leora D’Souza, Aparajita Kumar, Kishan Kalia, Srilekha Prabhu, Kruttika Phalnikar, Puli Chandramouli Reddy, Bhavana Muralidharan

**Affiliations:** Institute for Stem Cell Science and Regenerative Medicine (inStem), Bangalore, India; Regional Centre for Biotechnology, Faridabad, India; Institute for Stem Cell Science and Regenerative Medicine (inStem), Bangalore, India; Institute for Stem Cell Science and Regenerative Medicine (inStem), Bangalore, India; Institute for Stem Cell Science and Regenerative Medicine (inStem), Bangalore, India; Institute for Stem Cell Science and Regenerative Medicine (inStem), Bangalore, India; Institute for Stem Cell Science and Regenerative Medicine (inStem), Bangalore, India; Centre of Excellence in Epigenetics, Department of Life Sciences, Shiv Nadar Institution of Eminence, Delhi, NCR, India; Institute for Stem Cell Science and Regenerative Medicine (inStem), Bangalore, India

**Keywords:** LSD1/KDM1A, histone-modifier, extracellular matrix proteins, cell adhesion genes, Notch signaling pathway, human neuronal development, human-specific mechanisms

## Abstract

Neurogenesis begins with neural stem cells undergoing symmetric proliferative divisions to expand and then switching to asymmetric differentiative divisions to generate neurons in the developing brain. Chromatin regulation plays a critical role in this switch. Histone lysine-specific demethylase LSD1 demethylates H3K4me1/2 and H3K9me1/2 but the mechanisms of its global regulatory functions in human neuronal development remain unclear. We performed genome-wide ChIP-seq of LSD1 occupancy, RNA-seq, and Histone ChIP-seq upon LSD1 inhibition to identify its repressive role in human neural stem cells. Novel downstream effectors of LSD1 were identified, including the Notch signaling pathway genes and human-neural progenitor-enriched extracellular matrix (ECM) pathway/cell adhesion genes, which were upregulated upon LSD1 inhibition. LSD1 inhibition led to decreased neurogenesis, and overexpression of downstream effectors mimicked this effect. Histone ChIP-seq analysis revealed that active and enhancer markers H3K4me2, H3K4me1, and H3K9me1 were upregulated upon LSD1 inhibition, while the repressive H3K9me2 mark remained mostly unchanged. Our work identifies the human-neural progenitor-enriched ECM pathway/cell adhesion genes and Notch signaling pathway genes as novel downstream effectors of LSD1, regulating neuronal differentiation in human neural stem cells.

## Graphical abstract

**Graphical Abstract:**
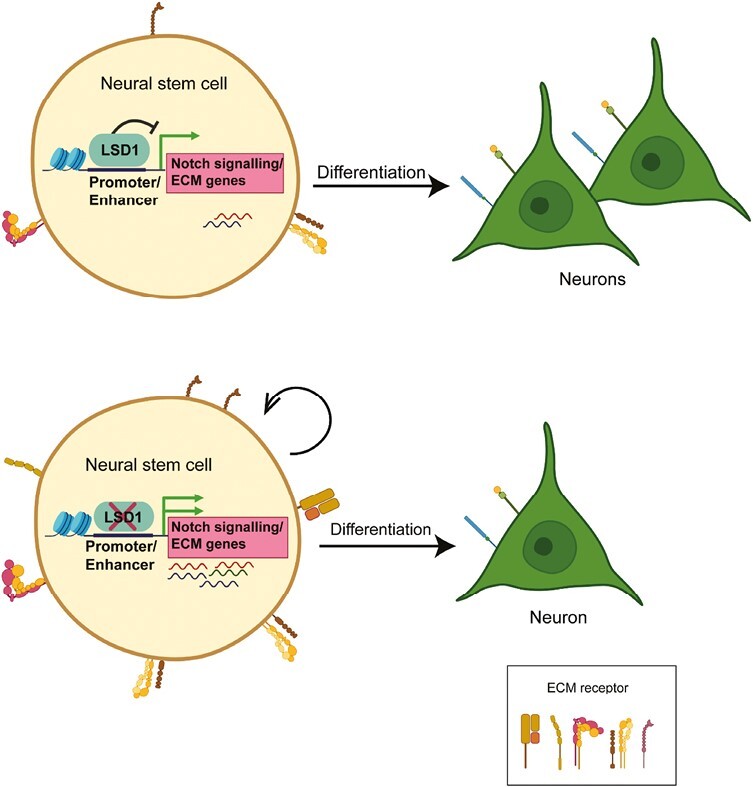

**Significance Statement**

The billions of neurons in the brain originate from neural stem cells, which exhibit similarities and differences in mouse and human neurogenesis. Our research demonstrates that the highly conserved chromatin modifier LSD1 primarily functions as a repressor, regulating human progenitor-enriched extracellular matrix (ECM), and cell adhesion genes involved in neuronal differentiation. These genes also display enriched H3K4me2 marks in the promoter/transcription start site (TSS) regions, identifying them as novel targets for the histone-demethylating enzyme LSD1.

## Introduction

Dynamic modulation of chromatin status via epigenetic regulation is critical for driving gene expression in neurodevelopmental time and space.^1,2^ Activation and repression of key gene regulatory networks are required for the rapid expansion of the progenitor pool by proliferation followed by the onset of neurogenesis, neuronal differentiation, and maturation.^3,4^ Regulation of gene expression is mediated by changes in DNA methylation and histone tails,^5,6^ which alter the landscape of chromatin accessibility.

Core histone proteins undergo various post-translational modifications, well-studied of which are phosphorylation, acetylation, and methylation^7^ These modifications enable alternation of chromatin structure, which is critical for inducing transcriptional responses. Methylations of the lysine residues of histone are particularly crucial for the regulation of developmental genes.^8^ These modifications are enabled by histone writers (methyl transferases) and erasers (demethylases).^9,10^

LSD1/KDM1A-lysine-specific histone demethylase 1 is the first demethylase to be identified as part of the C-terminal binding protein 1 (CtBP1) corepressor complex.^11^ It is a flavin adenine dinucleotide-dependent amine oxidase and is part of the Nucleosome Remodelling and Deacetylase (NuRD) complex and co-REST complex.^12,13^ It removes methyl groups from lysine residues in histones, specifically from the mono- or di-methylated lysines on histone 3 (H3K4me1/2 and H3K9me1/2), functioning as both an activator and a repressor. LSD1/KDM1A demethylates active H3K4me2 and repressive H3K9me2 marks.^14,15^

In murine corticogenesis, LSD1 controls the proliferation of apical radial glia (aRG) in the ventricular zone and subsequent neuronal migration.^12,16^ Knockdown of *LSD1* results in defective proliferation of the progenitors followed by cell cycle exit and premature neuronal differentiation. The *LSD1* knockdown brains also show a reduction in the number of progenitors.^16^

Conversely, inhibition of LSD1 function in fetal human neural stem cells leads to reduced neurogenesis and increased progenitor proliferation. Thus, LSD1 is necessary for human neuronal differentiation.^17^ LSD1 mediates this function by inhibiting *HEYL*—a Notch downstream effector in human neural stem cells.^17^ In recent years, research has highlighted significant differences between human and murine brain development. These differences include prolonged developmental timing, increased number and diversity of progenitors, slow neuron maturation rate, expansion of cortical parenchyma, and human-specific behavioral phenotypes.^18-21^ Epigenetic regulatory networks, novel transcription factors and enhancers, and the acquisition of novel target genes for conserved genes may have driven the evolution of these distinctively human aspects of cortical development.^22,23^

Additionally, human-specific and human brain-enriched genes may explain some of the unique traits of humans.^24-27^ Genes linked to cell adhesion and extracellular matrix (ECM) components are among these genes with enriched expression in the human brain.^28^

LSD1 is in the top 2% of evolutionarily constrained genes and is conserved across different species.^29^ Evolutionarily constrained genes are under strong selective pressure to remain intact and functional. Yet, its function is different between mouse and human neural progenitor cells. This suggests that its downstream effector genes may be different between the two species.

Therefore, to explore the molecular mechanisms by which LSD1 regulates neurogenesis in human neural stem cells, we conducted a genome-wide occupancy analysis of LSD1 in the chromatin of these progenitors along with global transcriptomic profile and epigenetic changes upon LSD1 inhibition.

LSD1 inhibition resulted in the upregulation of several genes of the Notch signaling pathway namely transcription factors such as *HES6* and *TLE1*. Several human neural progenitors enriched ECM component and cell adhesion genes, namely, *LGALS3BP*, *SERPINE1*, and *NOTUM* were also significantly upregulated upon LSD1 inhibition.

Reduced neurogenesis was observed as a result of LSD1 inhibition, and overexpression of downstream effector genes mimicked this inhibition and resulted in reduced neurogenesis. We further analyzed changes in the genome-wide occupancy of the marks regulated by LSD1, namely H3K4me1/2 and H3K9me1/2, upon LSD1 inhibition. Our results revealed that LSD1 inhibition specifically increased active and enhancer marks, namely, the H3K4me1/2 and the H3K9me1, globally and did not affect the repressive H3K9me2 marks suggesting that LSD1 functions as a repressor in regulating neurogenesis. We also observed distinct upregulation of H3K4me2 and H3K4me1 marks at the TSS (transcription start site) and enhancers respectively of our downstream effector genes and almost no or minimal change in the H3K9me2 mark.

Using human neural stem cells (hNSCs), we have reported for the first time that LSD1 binds and functionally regulates several downstream effector genes involved in Notch signaling and human neural progenitor enriched genes in the cell adhesion and extracellular matrix organization.

## Materials and Methods

### hNSCs Culture and Maintenance

XCL1-human neural stem cells (XCL1-NSCs, Lifeline Cell Technology—IC-0001) were cultured on Matrigel (Corning—354277) coated dishes in a medium consisting of a 1:1 mixture of DMEM/F-12 (Gibco—21331020) and neurobasal (Gibco—21103049) media supplemented with N2 (Gibco—17502048), B27 without vitamin A (Gibco—12587010), insulin (Invitrogen—12585014), glutaMAX (Gibco—35050061), penicillin–streptomycin (Gibco—15140-122), β-mercaptoethanol (Gibco—31350010), sodium pyruvate (Gibco—11360070), non-essential amino acids (Gibco—11140050), and fibroblast growth factor (FGF, Peprotech—100-18B). For seeding cells for in vitro assays, we used StemPro Accutase (Gibco-A1110501) to dissociate the cells to attain a single-cell suspension for counting.

### hNSC Differentiation into Neurons and Astrocytes

For neuronal differentiation, hNSCs well were seeded in wells coated with poly-d-lysine (0.1 mg/mL) (Sigma—P7280-5MG) and laminin (10 µg/mL) (Invitrogen—23017015) in a medium containing of a 1:1 mixture of DMEM/F-12 (Gibco—21331020) and neurobasal (Gibco—21103049) media supplemented with N2 (Gibco—17502048), B27 (Gibco—17504044), insulin (Invitrogen—12585014), glutaMAX (Gibco—35050061), penicillin–streptomycin (Gibco—15140-122), β-mercaptoethanol (Gibco—31350010), sodium pyruvate (Gibco—11360070), non-essential amino acids (Gibco—11140050), hereafter called “N2–B27 differentiation media without FGF” and BDNF and GDNF were added to the media at the concentration of 10 ng/µL. Cells were differentiated for 14 and 21 days in vitro and media change was done on alternate days.

For astrocyte differentiation, hNSCs were cultured in DMEM (+l-glutamine) (Gibco—11995040) media supplemented with N2 and 2% FBS (Gibco—16141079) for 14 and 21 days in vitro, media change was performed on alternate days. Immunostaining was performed for different neuronal and astrocyte markers.

The following primary antibodies were used at 1:1000 dilution:

**Table:** 

Antibody	Host species and Cat. No
DCX	Rabbit, Abcam—ab18723
TUJ1	Rabbit, CST-#5666
TBR1	Chicken, Merck-AB2261
TLE4	Mouse, Santa Cruz-sc-365406
CTIP2	Rat, Abcam-ab18465
SATB2	Mouse, Abcam-ab51502
BRN2	Rabbit, CST-12137S
GFAP	Mouse, Sigma-G3893
S100B	Chicken, Synaptic systems-287 006

### Plasmid Construct Generation

Full-length human CDS of *HES6*, *TLE1*, *SERPINE1*, *NOTUM*, and *LGALS3BP* were amplified using Platinum SuperFi II Green PCR Master Mix (Invitrogen—12369050) and cloned into *pCAG-IRES-EGFP* (a kind gift from Prof Gordon Fishell, Harvard Medical School). Inserts were confirmed by Sanger sequencing.

### hNSCs LSD1 Inhibitor Assay

hNSCs (5 × 10^4^) were seeded in coated wells in hNSC maintenance media containing FGF. After 4 hours of seeding, the cells were treated with either vehicle (nuclease-free water) or 10 µm of GSK-LSD1 (Merck—SML1072-5MG) in N2-B27 differentiation media without FGF. Fresh media containing 10 µm GSK-LSD1 or vehicle was replaced every day. For qPCR analysis, RNA-seq and histone-ChIP-seq cells were harvested in 48 hours and for immuno-cytochemistry cells were harvested in 7 days. For EdU assays, cells were treated with 10 µm EdU for 2 hours before fixing and immunostaining. For assessing cell death, propidium iodide (1 µg/mL) treatment was done for 30 min.

### Western Blotting

XCL1-hNSCs treated with vehicle and 10 µm GSK-LSD1 were harvested after 48 hours for western blot analysis. Cells were lysed in RIPA buffer (25 mM Tris-HCl, pH 7.6, 150 mM NaCl, 1% NP40, 0.5% sodium deoxycholate, 0.1% SDS) containing protease inhibitor cocktail (Sigma—P8340). An equal amount of protein was resolved on a 15% SDS-polyacrylamide gel and electroblotted onto a PVDF membrane (Sigma—3010040001). The membrane was blocked in 5% BSA (Sigma—A7030) for 1 hour at room temperature. The membrane was incubated with primary antibodies (H3K4me2, Diagenode—C15410035, 1:2000; H3K27ac, Diagenode—C15410174; 1:2000, and Lamin B1, 1:2000, Abcam—ab16048) diluted in 3% BSA overnight at 40 °C followed by washes with TBST and incubation with respective HRP-conjugated secondary antibodies. Protein bands were detected using SuperSignal West Pico PLUS Chemiluminescent Substrate kit (Thermo Fisher Scientific-34580) images were taken using the iBright FL 1000 imaging system.

### ChIP Sequencing

ChIP-seq was performed as previously described.^95^ Briefly, hNSCs were harvested and were dual cross-linked with 2 mM disuccinimidyl glutarate (DSG, Proteochem—c1104-100 mg) for 30 min, followed by 1% formaldehyde (Invitrogen—28906) for 8 min and finally quenched with 0.125 M glycine for 5 minutes. The fixed cells were lysed in cell lysis buffer (10 mM Tris-HCl, pH 8.0, 10 mM NaCl, and 0.5% NP40) followed by nuclear lysis buffer (50 mM Tris-HCl, pH 8.0, 10 mM EDTA, 1% SDS, and 1% NP40). Chromatin was sheared using the ultrasonicator (Covaris—S220) to achieve an average fragment length of 100-300 bp. One hundred micrograms of sheared chromatin were used for immunoprecipitation with LSD1 antibody (Abcam—ab17721) and 10% of chromatin was stored as input. The chromatin–antibody complexes were pulled down using Dynabeads A and G (Invitrogen—10002D, 10004D) used at a 1:1 ratio at 4 °C overnight. The beads were then serially washed with low salt buffer (20 mM Tris-HCl, pH 8.0, 150 mM NaCl, 2 mM EDTA, 0.1% SDS, 1% Triton X-100—3×), high salt buffer (20 mM Tris-HCl, pH 8.0, 200 mM NaCl, 2 mM EDTA, 0.1% SDS, 1% Triton X-100—2×), LiCl buffer (0.25 M LiCl, 1 mM EDTA, 10 mM Tris-HCl, pH 8.0, 1% NP-40, 1% sodium deoxycholate—1 wash), and TE buffer (10 mM Tris-HCl, pH 8.0, 1 mM EDTA—2×). Chromatin was eluted by incubating the beads at 65 °C for 30 minutes at 80g in 300 µL of elution buffer (0.1 M NaHCO_3_, 1% SDS) and reverse crosslinked using 300 mM sodium chloride and RNAseA at 65 °C at 52g rpm overnight. The samples were then treated with 2 µL proteinase K (20 mg/mL), 20 µL of 1 M Tris-HCl, pH 8.0 and 10 µL of 0.5 M EDTA at 42 °C for 1 hour at 52g. The DNA was further purified using phenol-chloroform-isoamyl alcohol and ethanol precipitated along with glycoblue coprecipitant (Invitrogen—AM9516). Precipitated DNA was quantified using a Qubit4 fluorometer (Thermo Fisher Scientific). Each sample yielded approximately 33 million single-end reads, with a consistent distribution of reads across samples.

### Histone ChIP-Sequencing

Cells were cross-linked using 1% formaldehyde for 1 minute followed by 5 minute quenching in 0.125 M glycine. ChIP sequencing was performed as above using H3K4me1 (Diagenode—CS-037-100), H3K4me2 (Diagenode—C15410035), H3K9me1 (Abcam—ab8896), and H3K9me2 (CST—9753S) antibodies. Library preparation, sequencing, and analysis were performed as described below. Each sample yielded an average of 17 million single-end reads, with a consistent distribution of reads across samples.

Primers used (5ʹ-3ʹ)

**Table:** 

Gene	Forward primer	Reverse primer
*HES6*	TTTTGGCAAAGAATTCATGGACTACAAAGACGATGACGACAAGGCGCCACCCGCGGCG	AAATGATATCGAATTCTCACCAAGGCCTCCAGACACTCC
*TLE1*	TTTTGGCAAAGAATTCATGGACTACAAAGACGATGACGACAAGTTCCCGCAGAGCCGGC	AAATGATATCGAATTCTCAGTAGATGACTTCATAGACTGT
*SERPINE1*	TTTTGGCAAAGAATTCATGGACTACAAAGACGATGACGACAAGCAGATGTCTCCAGCCC	AAATGATATCGAATTCTCAGGGTTCCATCACTTGGCC
*NOTUM*	TTTTGGCAAAGAATTCATGGACTACAAAGACGATGACGACAAGGGCCGAGGGGTGCGC	AAATGATATCGAATTCCTAGCTTCCGTTGCTCAGCATCCC
*LGALS3BP*	TTTTGGCAAAGAATTCATGGACTACAAAGACGATGACGACAAGACCCCTCCGAGGCTC	AAATGATATCGAATTCCTAGTCCACACCTGAGGAGTTGG
*HEYL*	CATCGACGTGGGCCAAGAG	CGCCGTTTCTCTATGATCCCT
*GAPDH*	CTGACTTCAACAGCGACACC	TAGCCAAATTCGTTGTCATACC

### Library Preparation, Sequencing, and Data Analysis

Libraries were prepared using NEBNext Ultra II DNA Library Prep with Sample Purification Beads (E7103L) and sequencing was performed on the Illumina HiSeq 2500. Previously described analysis pipelines were used.^96,97^ Specifically, for QC of reads pipeline described in Andrews (2010),^98^ for trimming (^99^; parameters—u 5), for alignment^100^ (BWA v0.7.17), for peak calling,^101^ for annotation of peaks to genes—HOMER^102-104^ were used. Samtools^100^ (v. 1.6) and BEDTools (v. 2.25.0)^105^ were used to interconvert and handle the aligned read files.

DeepTools v3.1.3 was used to generate BigWig files, Metagene plots, and ChIP-seq heatmaps.^106^ For the downstream analysis input was subtracted from the relevant sample readings using deep Tools bamCompare function. ChIP sequencing was performed for 2 biological replicates (*n* = 2).

The motif analysis was performed using MEME-ChIP^107^ using the default settings. The tracks were visualized using the Integrated Genomics Viewer (IGV) browser.^108^ The G: Profiler tool in the R package was used to produce gene associations, GO keywords, and KEGG pathways for LSD1-bound elements.^109^ The reference genome used was the human genome (GRCh37).

### RNA Sequencing and Analysis

RNA was extracted from cells using TRIzol as per the manufacturer’s protocol (Invitrogen—15596018). The experiment was performed for 3 biological replicates (*n* = 3). RNA quality check was performed using the Agilent 2100 Bioanalyzer System. One microgram of isolated RNA was used to prepare a cDNA library using the NEBNext Ultra II Directional RNA Library Prep with Sample Purification Beads (Cat. No.-E7765L). The cDNA libraries were sequenced using HiSeq 2500 for high-throughput DNA sequencing. Sequencing yielded around 40 million single-end reads per sample. Analysis of the RNA seq data was performed as per the previously published analysis pipeline.^97,110^ Briefly, for QC of reads pipeline described in (Andrews, 2010^98^), for trimming (Martin, 2011^99^; parameters—u 5), for alignment STAR v2.7.3a.^111^ MultiBamCov^105^ a component of BEDTools was used to generate read count matrix files for exons/genes using Ensembl release 104 (GRCh37) gene annotations. Differential transcript analysis was performed using EdgeR (57) on the R platform (v3.4.0). Log2-fold change ≥ 0.32 and FDR < 0.05 was used as a cut-off to get DEGs.

### Nucleofection

Cells (2 × 105) were nucleofected with 1 µg of plasmid DNA in nucleocuvettes as per the manufacturer’s protocol (Lonza—V4XP-3032) with the CA137 program and plated into a well of an 8-well chamber slide coated with poly-d-lysine and laminin. Media change was performed every alternate day and the cells were maintained in neuronal differentiation media for 7 days. For EdU assays, cells were treated with 10 µm EdU for 2 hours before fixing and immunostaining. For assessing cell death, propidium iodide (1 µg/mL) treatment was done for 30 minutes.

### Immunocytochemistry

For immunostaining, cells were fixed with 4% PFA in PBS for 10 minutes at room temperature, followed by quenching with 20 mM glycine and 75 mM ammonium chloride. The cells were blocked and permeabilized using PBS containing 10% donkey serum (Abcam—ab7475) and 0.1% Triton X-100 (Sigma—T8787) for 30 minutes at 37 °C. They were then incubated with primary antibodies in PBS with 5% donkey serum and 0.1% Triton X-100 overnight at 4 °C. After 3 washes with PBS for 5 minutes each, the cells were incubated with appropriate Alexa Fluor secondary antibodies for 2 hours at 37 °C. The cells were then rinsed another 4× with PBS/0.1% Triton X-100 for 5 minutes before counterstaining with DAPI (Invitrogen—D1306) for 10 minutes at room temperature. The slides were again washed 3× with PBS and mounted with fluoro shield (Merck-F6182). The cells were immunostained using the following primary antibodies against PAX6 (1:200; rabbit, Biolegend—901301), and NESTIN (1:2000; mouse, Merck—mab5326), or SOX2 (1:500 or 1:1000; mouse, Santa Cruz—sc365823), LSD1 (1:1000; rabbit, Abcam—ab17721), or DCX (1:3000, rabbit, Abcam—ab18723), and GFP (goat biotinylated, Abcam-ab6658 or mouse, Invitrogen—A11120). The following secondary antibodies were used at 1:1000—donkey anti-rabbit Alexa Fluor 488 plus (Invitrogen—A32790), donkey anti-mouse Alexa Fluor plus 488 (Invitrogen—A32766), donkey anti-rabbit Alexa Fluor plus 555 (Invitrogen—A32794), and donkey anti-mouse Alexa Fluor plus 555 (Invitrogen—A32773). The images were acquired at 20× or 40× magnification on an Olympus FV3000 confocal microscope with FV31S-SW 2.1 213 viewer software and analyzed using Fiji ImageJ software (Version 1.52n) and the cell counter plugin. EdU Click-iT assay was performed using EdU-Click-iT plus cell proliferation assay kit (Invitrogen—C10638) using the manufacturer’s protocol.

### Statistical Analysis

Biological replicates used for statistical tests are reported in each figure legend. All statistical analyses were performed using GraphPad Prism v9.4.0 (GraphPad Software) or R package. Parametric data were analyzed by unpaired 2-tailed Student’s *t*-test. Data are presented as mean ± SEM and regarded statistically significant if *P* < .05.

## Results

### Identification of LSD1 Genome-Wide Occupancy in Human Neural Stem Cells

**Figure 1.**
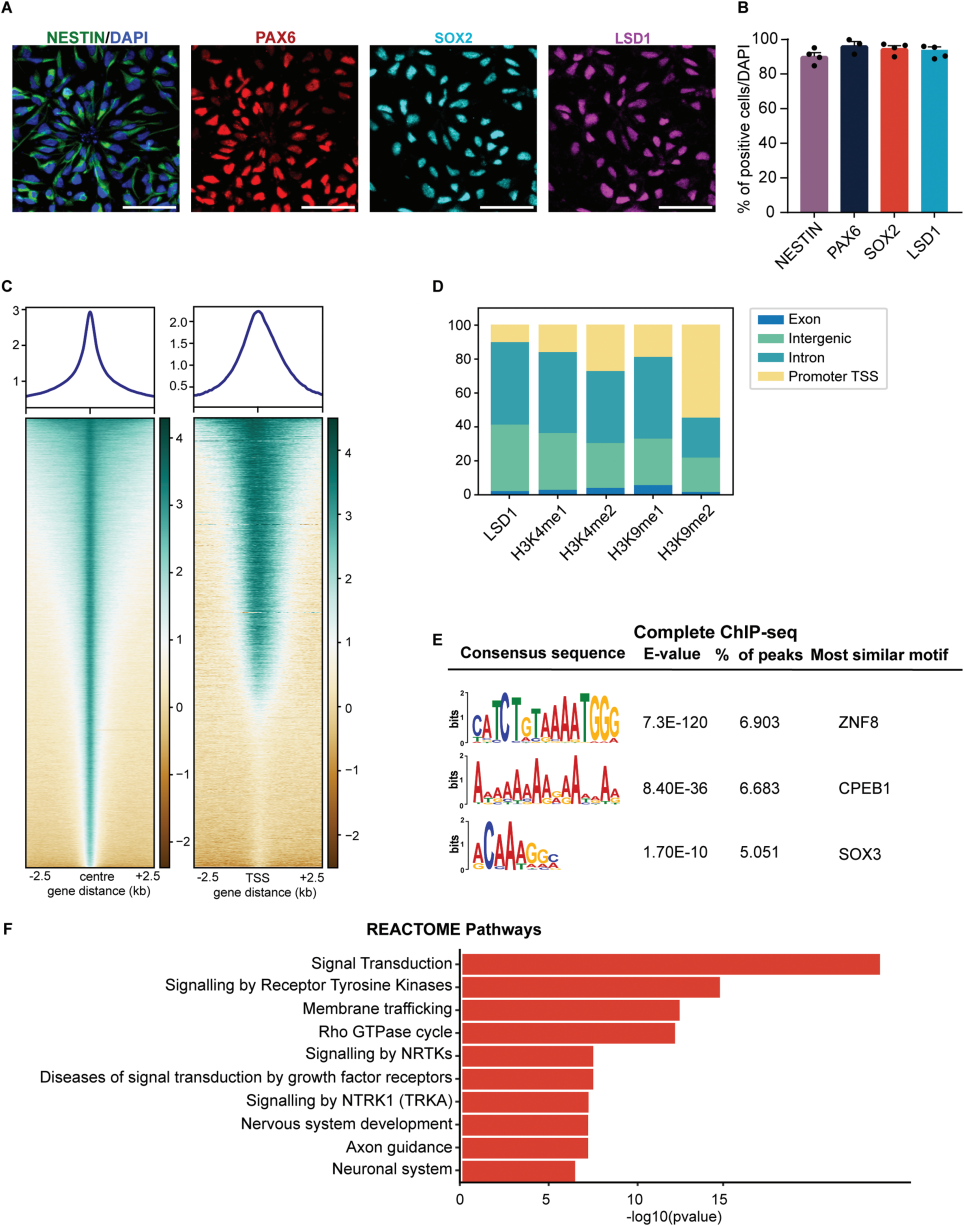
Genome-wide occupancy analysis of LSD1 in human NSCs. (**A**) Confocal images showing the expression of NSC markers NESTIN, PAX6, and SOX2; and LSD1. Nuclei were counterstained with DAPI (blue). Scale bar 50 µm. (**B**). Quantification of (A), *n* = 4 independent experiments. (**C**) Heat map showing the ChIP-seq read density for LSD1 occupancy within a region spanning ± 2.5 kb around the center (left panel) and TSS (right panel) of each annotated peak in hNSCs, each line in the heatmap represents an individual LSD1 binding site, shown above is the average profile plot of LSD1. (**D**) Distribution of LSD1 ChIP-seq peaks and overlap of LSD1 peaks with corresponding histone marks ChIP-seq peaks for H3K4me1, H3K4me2, H3K9me1 and H3K9me2, across different genomic regions. (**E**) Motif enrichment analysis showing the most abundant DNA sequence motifs identified in LSD1 ChIP-seq peaks. (**F**). The REACTOME pathway enrichment analysis for LSD1-bound regions. Bar plots show the top 10 pathway categories.

Using human PSC-derived neural stem cells (hNSCs) XCL1-NSCs, we investigated the role of LSD1 in human neurogenesis. This model system has been previously utilized for in vitro studies on human neuronal differentiation.^30-32^ To confirm progenitor markers expression, we performed immunocytochemistry for PAX6, SOX2, and NESTIN in hNSCs (Fig. 1A). More than 90% of hNSCs expressed these markers (90.1% for NESTIN, 96% for PAX6, and 95% for SOX2) (Fig. 1B). LSD1 is expressed throughout the developing human neocortical primordium.^17^ We also observed LSD1 expression in these hNSCs, with 94% of hNSCs expressing the LSD1 protein (Fig. 1A, 1B). Furthermore, we show that these hNSCs can generate different cortical neuronal subtypes and astrocytes in vitro. We subjected the XCL1 NSCs to differentiation and conducted a temporal assessment of neuronal subtype marker expression at 2 time points, 14DIV and 21DIV of culture (Supplementary Fig. S1A). After 14 days of differentiation, we observed the temporal expression of deep layer markers, including TBR1, TLE4, and CTIP2, as well as pan-neuronal markers TUJ1 and DCX. At 21DIV, we observed the expression of superficial layer markers SATB2 and BRN2, along with the expression of astrocyte markers such as GFAP and S100β (Supplementary Fig. S1A).

To investigate the genome-wide DNA binding dynamics of LSD1 in human neural stem cells, we conducted chromatin immunoprecipitation followed by sequencing (ChIP-seq). Heatmap analysis revealed that LSD1 binding in hNSCs centered around peak regions, with 10% of these peaks found near gene transcription start sites (TSS; Fig. 1C). We identified 151,488 binding events across the genome, with the highest enrichment in intron and intergenic regions, accounting for 46% and 38% of binding sites, respectively. Promoter/TSS binding contributed to 10% of the binding (Fig. 1D).

We extracted all LSD1-bound peaks and annotated them using HOMER to identify downstream target genes. To uncover overrepresented functional cellular processes and pathways in this dataset, we performed DAVID GO biological pathway analysis on all LSD1 downstream effector genes, or exclusively genes with LSD1 bound to their TSS. The top relevant GO pathways included nervous system development, WNT signaling, axon guidance, and cell proliferation (Supplementary Fig. S1B, S1C).

LSD1 demethylates both H3K4me1/2 and H3K9me1/2. To examine the overlap between these marks and LSD1 binding sites in the genome, we performed histone ChIP-seq for H3K4me1, H3K4me2, H3K9me1, and H3K9me2 (Supplementary Fig. S5A). Upon intersecting the Histone ChIP-seq dataset with the LSD1 ChIP-seq dataset we observe 81% of H3K4me1, 69% of H3K4me2, and 76% of H3K9me1 are LSD1 bound and are present in intronic and intergenic regions combined (Fig. 1D). In contrast, only 44% of H3K9me2 marks were LSD1 bound and detected in introns and intergenic regions (Fig. 1D). This suggests that LSD1 in hNSCs is more frequently bound at regions marked by active (H3K4me2) and enhancer marks (H3K4me1 and H3K9me1) than the repressive H3K9me2 mark.

LSD1 functions as a chromatin remodeler and requires interaction with DNA-bound transcription factors.^33^ To identify likely transcription factors associated with LSD1-bound genomic regions, we performed de novo motif enrichment using MEME analysis. This revealed enrichment of an AT-rich ZNF8 binding motif, as well as binding sequences for RNA binding protein CPEB1 and transcription factor SOX3 (Fig. 1E). Additionally, MEME analysis of LSD1 peaks bound to TSS regions identified C-rich binding motifs for zinc finger transcription factors, including EGR1, KLF15, KLF12, and ZNF93. C-rich regions form intercalated motif structures called i-motifs. The presence of these structures in the promoter/TSS regions is typically associated with high transcriptional activity of the genes^34-36^ (Supplementary Fig. S1D).

Furthermore, functional enrichment analysis comparing our dataset with REACTOME pathways revealed that the top 10 pathways were signal transduction, nervous system development, axon guidance, and neuronal system, suggesting LSD1 plays a crucial role in human neurogenesis (Fig. 1F).

Histone modifiers regulate noncoding regulatory elements by adding or removing specific histone marks associated with transcriptional activation or repression leading to transcriptomic changes. Our LSD1 genome occupancy data demonstrates that LSD1 predominantly binds to distal regulatory elements in neural stem cells. To address its global role in gene expression we performed RNA-seq to identify up/downregulated genes upon its inhibition.

### Identification of LSD1 Direct Target Genes Demonstrates its Role as a Repressor of Signal Transduction, Transcription, Extracellular Matrix, and Cell Adhesion Genes

To identify genes directly regulated by LSD1, we compared binding sites from ChIP-seq with differentially expressed genes using RNA-seq on hNSCs in neuronal differentiation medium. hNSCs were treated with a specific LSD1 inhibitor (GSK-LSD1), and the effect on the transcriptome profile was examined (Supplementary Fig. S2A). GSK-LSD1 inhibitor, also known as OG-668, is a well-studied molecule known for its specific inhibition of LSD1 demethylase activity. Testing several known LSD1 inhibitors showed that GSK-LSD1/OG-668 displayed remarkable potency with a low IC50.^37^ Significantly, GSK-LSD1 was the sole inhibitor that did not affect other structurally related amine oxidases. Additionally, GSK-LSD1 was utilized to inhibit LSD1 function in an autism model of Shank3, effectively rescuing social deficits and behavioral symptoms of autism.^38^ It was also used in K562 leukemia cells to globally increase H3K4me2 levels.^39^

We assessed LSD1 inhibition efficacy by measuring mRNA transcript levels of a previously identified target gene, *HEYL*.^17^ GSK-LSD1 treatment increased *HEYL* mRNA expression in our experiment (Supplementary Fig. S2B). Additionally, we evaluated the specificity of the LSD1 inhibitor’s action on LSD1 by quantifying the global change in H3K4 dimethylation levels in hNSCs treated with GSK-LSD1. Our results revealed a significant increase (1.5-fold change) in H3K4me2 protein levels in GSK-LSD1-treated hNSCs compared to the vehicle-treated cells. The levels of H3K27ac showed no discernible difference between the 2 conditions (Supplementary Fig. S2C).

**Figure 2.**
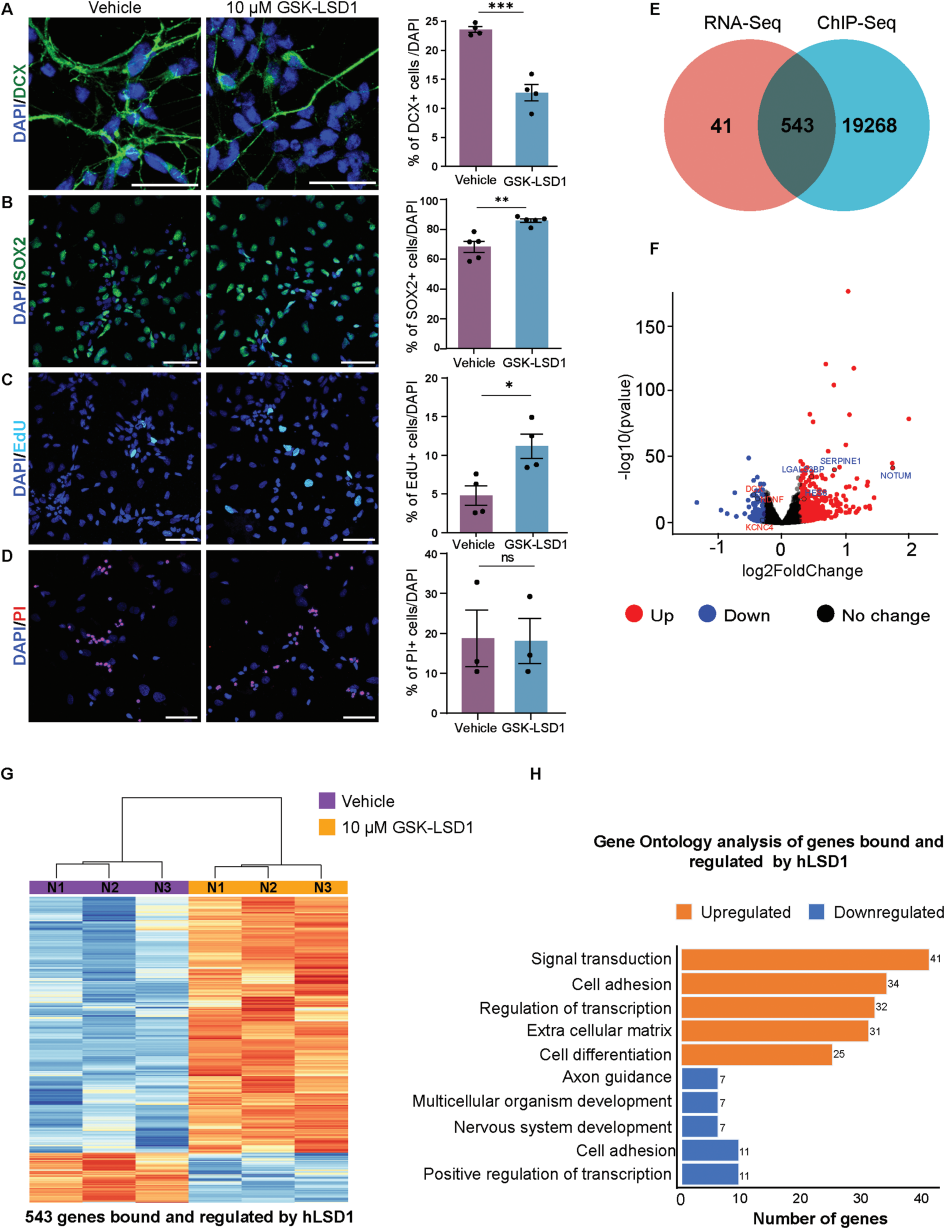
LSD1 primarily acts as a transcriptional repressor during neurogenesis. hNSCs were treated with vehicle or 10 µm GSK-LSD1 for 7 days under differentiation conditions. For quantifying cycling progenitors, an EdU pulse was given for 2 h before fixation and for assessing cell death, cells were treated with propidium iodide (PI) for 30 min before fixing. Representative confocal images (left) and quantification (right) showing DCX+ (*n* = 4) (**A**), SOX2+ (*n*  = 5). (**B**) EdU+ (*n* = 4). (**C**) and PI+ (*n* = 3) (**D**) cells in hNSCs treated with vehicle or GSK-LSD1 (left). Nuclei are counterstained with DAPI (blue). Scale bar 50 µm. Error bars represent SEM, ^*^P < .05, ^**^P < .01, ^***^P < .001. (**E**) Venn diagram showing the overlap between LSD1 bound and regulated genes. (**F**) Volcano plot representing differentially expressed genes in 10 µm GSK-LSD1 treated hNSCs compared with vehicle. (**G**) Heatmap showing the expression of LSD1 bound and regulated genes for 3 biological replicates. Five hundred and forty-three genes were direct targets and regulated by LSD1, of these, 455 genes were upregulated and 88 genes were downregulated. (**H**) Gene ontology analysis shows the enrichment of genes associated with signal transduction, cell adhesion, and extracellular matrix pathway genes.

We validated the impact of LSD1 inhibition on neurogenesis and observed a decrease in neuron generation upon GSK-LSD1 treatment (Vehicle-23.5%, GSK-LSD1-12.7%) (Fig. 2A). To determine whether the reduced neurogenesis is attributed to increased proliferation or apoptosis, we conducted EdU incorporation experiments, as described in the methods section. Additionally, we quantified the number of proliferating SOX2+ cells and assessed cell death using Propidium Iodide (PI) staining in hNSCs treated with either the vehicle or GSK-LSD1.

Our observations revealed a significant increase in the population of SOX2 + hNSCs (from 68.3% to 85.8%) and EdU-positive cycling progenitors (from 4.8% to 11.2%) under inhibitor treatment (Fig. 2B, 2C). We did not observe a substantial difference in the PI + cell count between vehicle and GSK-LSD1-treated cells (18.73% and 18.08%, respectively) (Fig. 2D).

These findings suggest that blocking LSD1 function prompts hNSCs to re-enter the cell cycle, resulting in the production of an increased number of proliferating progenitors, thereby leading to a reduction in neuronal production.

From our RNA-seq we identified 584 significantly dysregulated genes upon LSD1 inhibition, out of which 483 genes were upregulated and 101 genes were downregulated upon LSD1 inhibition (Supplementary Fig. S2D, S2E). To uncover regulatory mechanisms, we combined ChIP-seq and RNA-seq data to elucidate how LSD1 controls gene expression, as either an activator or repressor by correlating binding events with changes in gene expression levels. This comparison yielded 543 potential direct LSD1 target genes that were differentially expressed (Fig. 2E), including 454 upregulated and 88 downregulated genes (Fig. 2F, 2G, Supplementary Table S1). These findings suggest that LSD1 predominantly functions as a repressor, negatively regulating the expression of 454 genes (Fig. 2F, 2G).

Additionally, we conducted an analysis of LSD1 binding at various genomic regions for these dysregulated genes. Our findings indicate that intron and intergenic regions exhibited the highest number of peaks, followed by promoter/TSS regions. Specifically, among the 3797 LSD1 binding peaks associated with upregulated genes, 49.3% were located in introns, 37.4% in intergenic regions, 10.6% in promoters, and 2.7% in exons. For downregulated genes, 49.2% of the 906 LSD1 peaks were found in intergenic regions, 38.8% in introns, 9.5% in promoters, and 2.4% in exons (Supplementary Fig. S2F).

To gain insights into the molecular pathways and biological processes modulated by LSD1, we performed GO biological processes (BP) enrichment analysis on the combined ChIP-seq and RNA-seq datasets. The analysis revealed that the top 4 upregulated GO BP pathways upon LSD1 inhibition were related to signal transduction, cell adhesion, transcription regulation, and extracellular matrix components/organization (Fig. 2H; Supplementary Table S2). In contrast, 2 key pathways downregulated upon LSD1 inhibition were associated with nervous system development and axon guidance. Notably, several crucial neuronal genes, such as DCX (Doublecortin), NDNF (neuron-derived neurotrophic factor), and KCNC4 (Potassium Voltage-Gated Channel Subfamily C Member 4), exhibited significantly reduced expression following LSD1 inhibition (Fig. 2F; Supplementary Table S1).

### LSD1 Directly Binds and Represses Notch Signaling Downstream Effectors to Facilitate Neurogenesis

**Figure 3.**
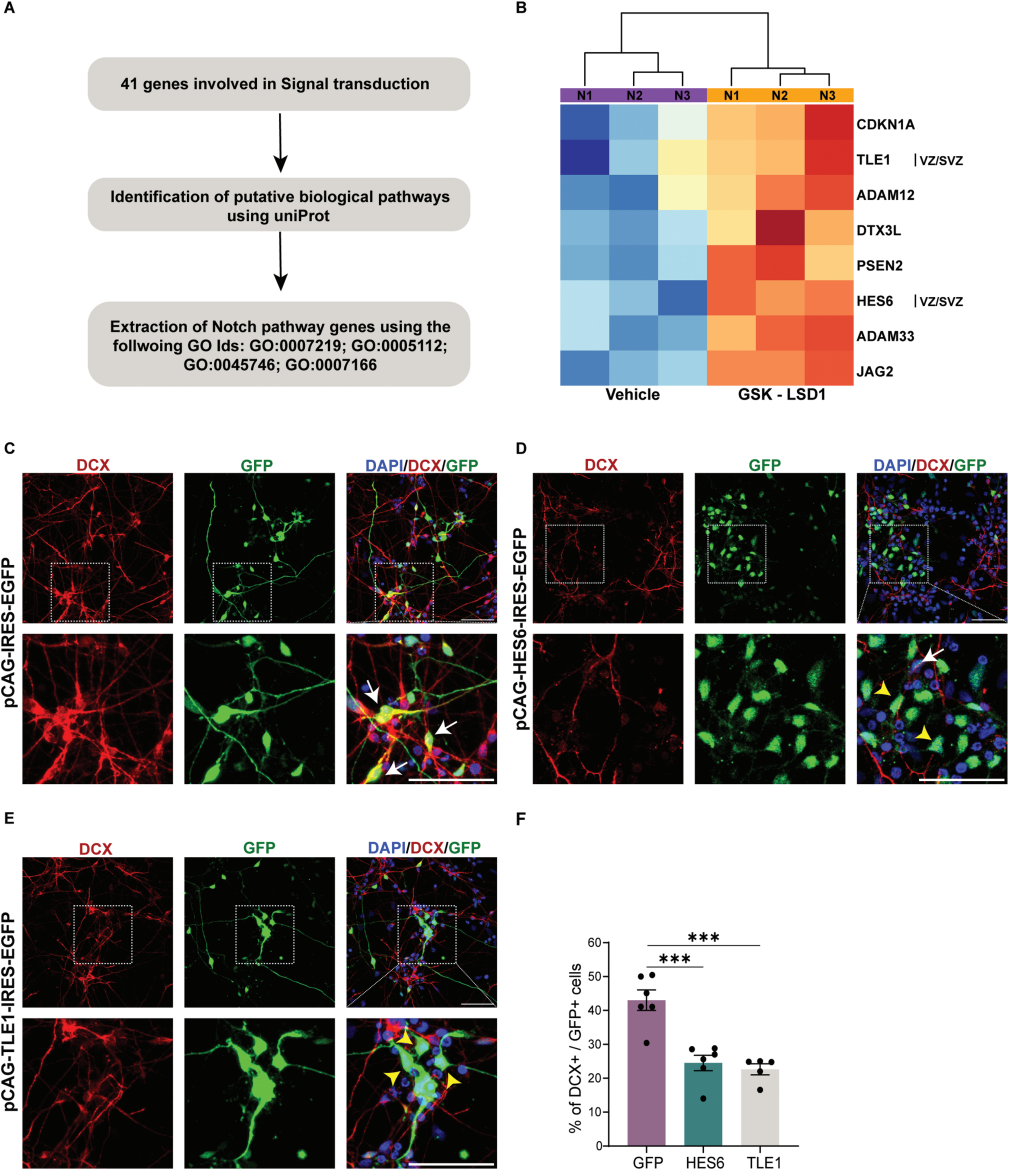
LSD1 negatively regulates the Notch signaling pathway. (**A**) Schematic showing the gene selection criteria. (**B**) Heatmap depicting the expression of the Notch signaling pathway genes under GSK-LSD1 treatment condition against the vehicle in hNSCs for 3 biological replicates. Overexpression of *HES6* and *TLE1* can mimic LSD1 inhibition. Control *EGFP* vector (**C**) *HES6* (**D**) and *TLE1* (**E**) overexpressing hNSCS were differentiated for 7 days. Top: Representative confocal images showing the expression of the neuronal marker DCX and GFP. Bottom: Corresponding high magnification of boxed regions showing the colocalization of DCX and GFP. Double-stained cells are indicated with white arrows and cells positive for GFP alone are indicated with yellow arrowheads. Nuclei were counterstained with DAPI (blue). Scale bar 50 µm. (**F**) Quantification of the number of DCX + GFP + cells in (C--E) shows a decrease in neurons in HES6 and TLE1 over pressing cells compared to the control (*n* = 5 or 6). Error bars represent SEM, ^*^P < .05, ^**^P < .01, ^***^P < .001.

To elucidate the mechanisms by which LSD1 promotes neuronal differentiation, we examined 41 signal transduction-enriched genes and analyzed their association with various biological pathways using UniProt. We observed that many genes from the Notch signaling pathway were enriched in this cohort (Fig. 3A, 3B). Several Notch signaling pathways and downstream effector genes were found to be bound by LSD1 and upregulated in hNSCs treated with GSK-LSD1 (Fig. 3B).

The Notch signaling pathway plays a significant role in neural stem cell survival, maintenance, and proliferation.^40^ Cortex-specific knockout of the *Notch1* receptor resulted in the depletion of apical progenitors and premature neuronal differentiation.^41^*NOTCH2NLB*, a human-specific paralog of the *NOTCH2* gene highly expressed in radial glia cells, when overexpressed in human NSCs and developing mouse cortex, leads to the expansion of cortical progenitors by activating the Notch pathway.^27,42^

LSD1 binds directly and represses several genes of the Notch signaling pathway namely *JAG2*, a Notch ligand, the Disintegrin Metalloproteases *ADAM12* and *ADAM13*, *PSEN2* (Presenilin2), a member of the *y* secretase complex, cell cycle inhibitor *CDKN1A*, and Notch downstream effector genes *TLE1* and *HES6* (Fig. 3B).

To investigate whether overexpression of Notch downstream effector genes mimics LSD1 inhibition and results in reduced neurogenesis in hNSCs, we overexpressed *TLE1* and *HES6* in hNSCs (Fig. 3D, 3E). hNSCs were nucleofected with the *pCAG-IRES2-EGFP* construct in which the *TLE1 and HES6* ORFs were cloned separately under the constitutive CAG promoter and the progenitors were allowed to differentiate 7 DIV. After 7 days of differentiation, the number of DCX-expressing immature neurons was assessed (Fig. 3F). DCX+ cells were significantly decreased to 23% and 22% under *TLE1* and *HES6* overexpression conditions, respectively, when compared to 41% in the control *GFP* nucleofection (Fig. 3C, 3F).

To understand the mechanism behind the reduced neurogenesis upon overexpression of downstream targets, we conducted EdU incorporation experiments, and quantified the number of proliferating SOX2+ cells and assessed cell death using propidium iodide (PI) staining. We observed an overall increase in the count of SOX2 and EdU-positive cells, extending beyond the nucleofected cells (Supplementary Fig. 3A). This observation suggests the presence of non-cell autonomous effects on proliferation. To confirm the proliferation increase, we assessed changes in the counts of SOX2 and EdU-positive cells by quantifying: (1) the total increase in cycling progenitors (SOX2 + EdU + progenitors normalized over the total DAPI count), (2) cell-autonomous increase in cycling progenitors: (SOX2 + EdU + GFP + progenitors normalized over GFP), (3) cell-autonomous increase in the number of progenitors (SOX2 + GFP progenitors normalized over GFP).

From this analysis, we observed that HES6 and TLE1 overexpression were effective in significantly increasing the total number of cycling progenitors (SOX2 + EdU) when normalized to DAPI and also exhibited cell-autonomous effects on the number of cycling progenitors and SOX2 + progenitors when normalized to GFP. There was no significant change in cell death as measured by PI staining (Supplementary Fig. 3B).

These results suggest that LSD1 promotes human neuronal differentiation by blocking the Notch signaling pathway. Specifically, HES6 and TLE1-both Notch downstream effectors inhibit neurogenesis by promoting the proliferation of cycling progenitors by both cell-autonomous and non-cell-autonomous mechanisms.

### LSD1 Directly Binds and Represses Human-Enriched ECM/Cell Adhesion Genes to Promote Human Neuronal Differentiation

**Figure 4.**
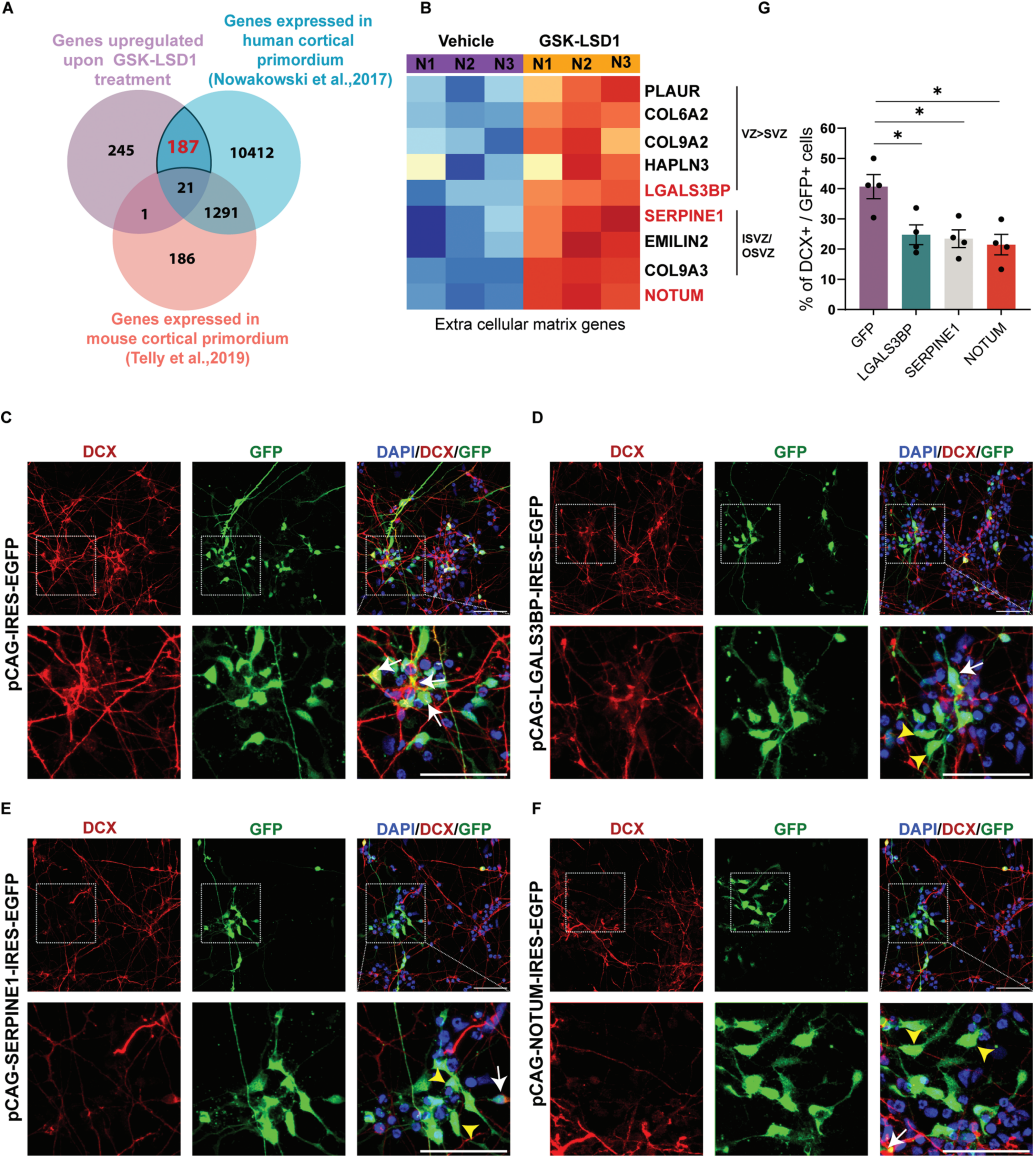
LSD1 controls neuronal differentiation via human brain-enriched ECM/cell adhesion genes. (**A**) Venn diagram summarizing the overlap between genes repressed by LSD1 in hNSCs, genes expressed in developing mouse cortex (data taken from Telly et al., 2019) and genes expressed in developing human cortex (data taken from Nowakowski et al., 2017) (**B**). Heat map depicting the enrichment of extracellular matrix (ECM) pathway genes in the human ventricular zone (hVZ), inner and outer subventricular zone (hISVZ/hOSVZ), in GSK-LSD1, treated hNSCs against the vehicle. (**C**–**F**) Overexpression of ECM-associated genes namely *LGALS3*BP (D), *SERPINE1* (E) or *NOTUM* in hNSCs phenocopies LSD1 inhibition. Top: Representative confocal images showing the expression of DCX and GFP in nucleofected hNSCs. Bottom: Corresponding high magnification of boxed regions showing the colocalization of DCX and GFP. DCX+ GFP+ cells are indicated with white arrows and only GFP+ cells are indicated with yellow arrowheads. Nuclei were counterstained with DAPI (blue). Scale bar 50 µm. (**G**) Graph depicting the number of DCX+ cells normalized to GFP expressing cells reveals a reduction in the neurogenesis upon *LGALS3BP*, *SERPINE1*, and *NOTUM* overexpression (*n* = 4). Error bars represent SEM, ^*^P < .05.

Several human-enriched genes play crucial roles in neurogenesis.^18,26,43^ They perform human-specific functions in the regulation of neural stem cell proliferation, differentiation, maturation, and contribute to human brain complexity.^21^ To identify unique human-enriched downstream target genes of LSD1, we compared bound and upregulated gene datasets with published human and mouse transcriptomic datasets.^44,45^ For human comparison we used single-cell RNA-Seq data from multiple germinal zones and the cortical plate of the developing human brain.^44^ For mouse comparison we used single-cell RNA-seq data from FlashTag pulse-labeled progenitors and neurons.^45^ We identified 187 LSD1 target genes (Supplementary Table S3) with human neural progenitor enriched or human-specific expression (Fig. 4A) and found several genes involved in cell adhesion and ECM organization pathways.

Previous studies have demonstrated that genes related to the extracellular matrix (ECM) and cell adhesion are enriched in the ventricular zone (hVZ) and subventricular zone (hSVZ) during the development of the human cortex^28^. The presence of these ECM genes supports the maintenance and self-renewal of the proliferating apical and basal progenitors in hVZ and the inner and outer SVZ (hISVZ/hOSVZ). Our investigation into the genes enriched in different germinal zones revealed an abundance of ECM genes regulated by LSD1 in either hVZ and/or hISVZ/hOSVZ (Figure 4B). Among these are key players such as the ECM receptor PLAUR^46^ collagen proteins COL9A2, COL9A3, and COL6A2^47^ HAPLN3 (Hyaluronan and Proteoglycan Link Protein 3), glycoproteins LGALS3BP and EMILIN2^48,49^ serine protease inhibitor SERPINE1,^50^ as well as the WNT antagonist, NOTUM.^51^

To investigate the effects of ECM/cell adhesion genes on neuronal differentiation we overexpressed select target genes in hNSCs. hNSCs were nucleofected with the *pCAG-IRES2-EGFP* construct in which the *SERPINE1*, *LGALS3BP*, and *NOTUM* ORFs were cloned under the constitutive CAG promoter and the progenitors were allowed to differentiate 7 DIV (Fig. 4D–4F). In hNSCs, we observed overexpression of *LGALS3BP*, *SERPINE1*, and *NOTUM* resulted in a drastic reduction in neurogenesis to 25%, 23.5%, and 21.5%, respectively (Figure 4G) as compared to NSCs expressing *GFP* alone (41%) (Figure 4C, 4G).

In our EdU incorporation experiments, we observe that the overexpression of LGALS3BP, SERPINE1, and NOTUM significantly increased the total number of cycling progenitors (SOX2 + EdU) when normalized to DAPI. Additionally, these overexpressions exhibited a cell-autonomous increase in progenitor proliferation when normalized to GFP, except for SERPINE1, which showed a significance value of *P* = .063. Moreover, there was a significant increase in the total number of cell-autonomous SOX2/GFP-positive progenitors upon overexpression. Importantly, there was no significant change in cell death, as measured by PI staining (Supplementary Fig. 4A, 4B).

Our results show that LSD1 facilitates neuronal differentiation in hNSCs by repressing human-enriched ECM/cell adhesion genes that are expressed in proliferative zones which are uniquely human. The various ECM and cell adhesion genes, both cell-autonomously or non-cell-autonomously, alter progenitor proliferation, ultimately resulting in reduced neurogenesis.

### Genome-Wide Histone ChIP-Seq Upon Inhibiting LSD1 Reveals an Upregulation of Active and Enhancer Marks

A genome-wide profiling study of histone modifications during corticogenesis revealed a significant difference in the enriched locus of H3K4me2 between human fetal brains and mice.^23^

Based on this finding, we hypothesized that the direct target genes of LSD1 in hNSCs, which we have identified as distinct from mice, may have a different epigenetic landscape between mice and humans. Using the published dataset,^23^ we analyzed the TSS and promoter regions of *SERPINE1*, *LGALS3BP*, and *TLE1* (Supplementary Fig. S5B, boxed regions) and found that the active H3K4me2 marks are highly enriched for *SERPINE1*, *LGALS3BP*, and *TLE1* in the analyzed genomic loci of the human fetal brain as compared to age-matched mouse embryonic brain.

LSD1 is a histone demethylase that demethylates H3K4me1/2 and H3K9me1/2. To gain a deeper understanding of the epigenetic regulatory mechanisms of LSD1 function, we conducted a global histone ChIP-seq analysis of marks that are directly regulated by LSD1 (Supplementary Fig. S5A). High-resolution functional annotation of human chromatin signatures mapped H3K4me2 to active promoters and H3K4me1 to enhancers.^52,53^ Analysis of hematopoietic stem cell chromatin revealed that H3K4me1 and H3K9me1 marks are associated with enhancers of differentiation genes before activation, suggesting a role in maintaining activation potential for differentiation.^54^ Nearly all eukaryotes use the repressive H3K9me2 mark to silence their genomes.^53^

**Figure 5.**
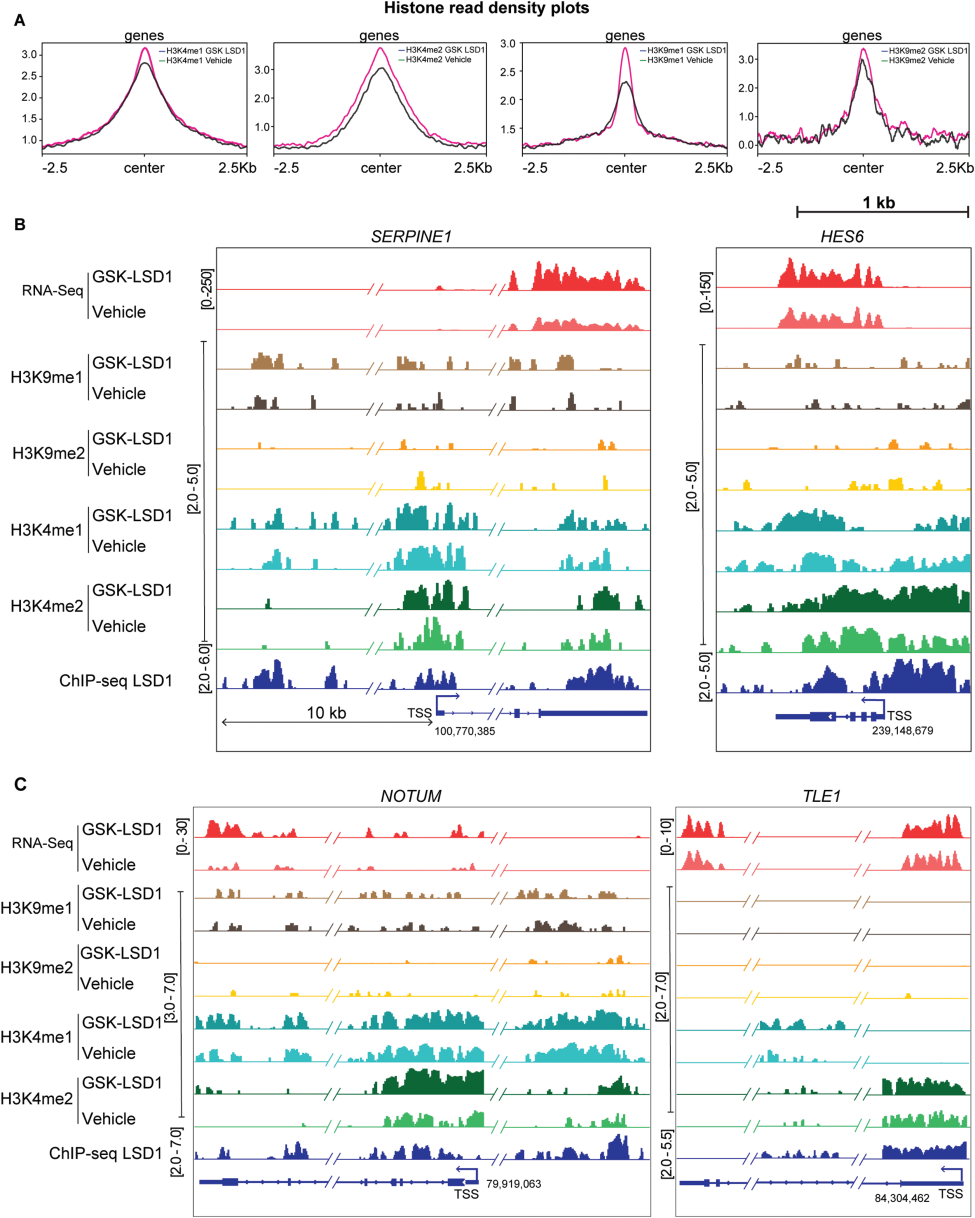
Genome-wide histone ChIP seq data upon LSD1 inhibition. (**A**) Read density profiles of H3K4me1/2 and H3K9me1/2 on gene bodies in the hNSCs treated with vehicle vs GSK-LSD1. Genome browser tracks illustrating the RNA-seq data, H3K9me2 ChIP-seq data, H3K4me2 ChIP-seq data, and LSD1 ChIP-seq data at the *SERPINE1*, *HES6* (**B**) NOTUM, and *TLE1* (**C**) loci following GSK-LSD1 treatment in hNSCs.

Upon inhibiting LSD1, histone density plots revealed a significant upregulation of histone modifications associated with active and enhancer loci (H3K4me2, H3K4me1, H3K9me1), whereas only a modest change was observed globally in the repressive H3K9me2 marks (Fig. 5A). Integrated genomics viewer (IGV) tracks of *SERPINE1*, *HES6*, *NOTUM*, and *TLE1* loci revealed an upregulation of H3K4me2 marks at TSS and LSD1 occupancy sites. Additionally, H3K4me1 and H3K9me1 marks showed an increase at putative distal regulatory regions, while H3K9me2 marks showed little or no change at these loci (Fig. 5B, 5C). In summary, inhibiting LSD1 in hNSCs upregulates active H3K4me2 marks, which leads to increased expression of genes involved in the ECM/cell adhesion genes and Notch pathway.

By integrating ChIP-seq, RNA-seq, and histone ChIP-seq data, we provide a comprehensive understanding of LSD1 binding, gene regulation, and the role of histone modifications in human neuronal differentiation. LSD1 functions by removing methyl marks from the active H3K4me2 from Notch signaling pathway genes and human-enriched ECM/cell adhesion genes to regulate neurogenesis in hNSCs.

## Discussion

Neurogenesis is a multicellular event coordinated spatially and temporally in the developing brain. One of the crucial factors that influence neuronal differentiation is the dynamic regulation of chromatin which affects the accessibility and the transcriptional activity of proliferative and neurogenic genes. Thus, the regulation of chromatin structure and function is essential for the proper maintenance of neural stem cells, differentiation, and maturation of neurons and for the establishment of proper neural connectivity.^1,55^

We utilized a comprehensive approach by integrating ChIP-seq, RNA-seq, and Histone ChIP-seq data to study genome occupancy, gene regulation, and the role of histone modifications in shaping the epigenetic landscape, to uncover novel human-enriched mechanisms, and interactions affecting human neuronal differentiation.

Our study shows that LSD1 binds to nervous and neuronal system genes in the hNSCs to regulate their function in neuronal cell fate specification. Combining LSD1 ChIP-seq with RNA-seq data revealed mechanisms of LSD1-mediated gene regulation. LSD1 acts as a repressor and negatively regulates signal transduction, cell adhesion, and regulators of transcription and ECM genes.

Upon binding with its cognate ligand (JAG/DLL),^56-59^ the Notch receptor protein undergoes proteolytic cleavage mediated by ADAM proteases and the γ-secretase complex, leading to the release of the Notch intracellular domain (NICD).^60^ The NICD translocate to the nucleus and activates downstream targets, including Hes and Hey.^56,61,62^ HES/HEY and TLE1-co-repressor then inhibit proneural genes such as *Mash1*, *Neurog2*, preventing neuronal differentiation and promoting progenitor proliferation.^63-66^ HES1, NOTCH4, and NOTCH1 also repress CDKN1A (Cyclin Dependent Kinase Inhibitor 1A), which is a negative regulator of neural stem cell proliferation,^67,68^ leading to progenitor proliferation.

Notch signaling is involved in regulating proliferative and neurogenic divisions of neural stem cells.^69-72^ Notch regulates a switch between symmetric and asymmetric division of neuroepithelial cells by regulating cell adhesion proteins such as cadherins in drosophila. Notch loss of function leads to delamination of neuroepithelial cells and their differentiation into neuroblasts.^73^ Hippocampal radial glial neural stem cells undergoing symmetric proliferative divisions show upregulated expression of Notch signaling.^74^

Put together Notch and its pathway genes have a critical role in symmetric proliferative divisions and is important to downregulate Notch to enable neurogenesis in the developing neocortex.^75^ Here we show that a histone demethylase LSD1 performs this critical function of Notch downregulation to enable neurogenesis. LSD1 binds to and negatively impacts several Notch pathway genes namely *JAG2*, *ADMA12*, *PSEN2*, *HES6*, *TLE1*, and *CDKN1A*. We compared our dataset with the data from Nowakowski et al. (2017), focusing on different cell-type clusters. Among the Notch signaling genes, TLE1 exhibited expression in radial glial cells, outer radial glial cells, as well as neurons. Conversely, HES6 was expressed in radial glial cells, outer radial glial cells, and intermediate progenitors. Additionally, ADAM33 demonstrated expression in truncated radial glial cells (tRG). While our comparison did not reveal significant expression differences among the various Notch target genes, it did highlight the shared expression of HES6 and TLE1 in apical radial glia. Given our interest in understanding LSD1’s function in progenitors, we specifically focused on HES6 and TLE1, both of which are expressed in apical radial glia. We validated functionally that HES6 and TLE1 overexpression phenocopies LSD1 inhibition and affected neuronal differentiation.

Mouse and human brains differ significantly in size, with progenitor proliferation and neuronal differentiation being key determinants of brain size.^76,77^ The initial size and type of neocortical progenitor pool, as well as subsequent neurogenesis, can greatly influence brain size.^6^ Compared to their primate counterparts, human cortical progenitors exhibit neoteny, with a longer period of balancing progenitor cell expansion and neurogenesis.^78^ The extended duration of human cortical neurogenesis is maintained in human cortical cells cultured in vitro, indicating that the mechanisms responsible for this process are primarily intrinsic to human cortical progenitors.^78,79^ Another aspect of cortical neurogenesis that underwent specific evolution in nonhuman primates and humans is the outer radial glia (oRGs)—a type of basal progenitors that are highly expanded in the human neocortex and almost absent in the mouse.^80^

ECM components have been shown to activate NSC proliferation through integrin signaling.^81^ Stimulation of the integrin α_v_β_3_ receptor in mouse basal progenitors has been found to increase intermediate progenitor proliferation.^82^ Cell adhesion and ECM genes are more expressed in the human neocortical progenitors in the germinal zones than in mice. The relevant genes associated with the extracellular matrix include specific sets of collagens, laminins, proteoglycans, and integrins, along with growth factors and morphogens.^24,28^

To identify uniquely human-enriched downstream effector targets of LSD1, we curated LSD1-bound and upregulated datasets with published mouse and human-developing brain single-cell RNA sequencing gene data. We found several ECM/cell adhesion genes to be upregulated upon LSD1 inhibition, with human-enriched expression. Some of these ECM/cell adhesion genes are expressed more in the VZ and some are only expressed in the ISVZ/OSVZ (data compared to Fietz et al.^51^ suggesting that LSD1 regulates human progenitor pool size) and subsequent neurogenesis by specifically regulating genes whose expression is in proliferative zones and are uniquely human.

NOTUM functions as a secretory deacylase, acting as an antagonist to the WNT signaling pathway. Its role is to regulate WNT signaling by inhibiting the interaction between Wnt ligands and Frizzled receptors.^51,83^ In addition, recombinant NOTUM overexpression has been shown to reduce the proliferation of SVZ progenitors in the adult mouse olfactory bulb.^84^

Serine proteases are recognized for their role in regulating neuronal migration, axon formation, and synaptic plasticity by influencing the proteolysis of the extracellular matrix.^31,85,86^ SERPINE1, an inhibitor of serine proteases, is crucial for maintaining the balance between ECM degradation and formation, which is essential for regulating these processes.^87,88^

Our analysis reveals that both NOTUM and SERPINE1 play roles in regulating the proliferation of cycling progenitors by both cell-autonomous and non-cell-autonomous mechanisms. Our study unveils a novel role for NOTUM and SERPINE1 in the differentiation of human cortical neurons. The mechanism of how these genes regulate human neural stem cell proliferation by interaction with its neurogenic niche needs further investigation.

LGALS3BP is a secretory glycoprotein involved in cell adhesion through interactions with various ECM proteins like laminins, collagens, fibronectin, and integrins.^48,89^ It is expressed in both apical and basal progenitors within the human cortex, where it plays a pivotal role in regulating proliferation, fate determination, and aRG to bRG cell transition.^25^ Loss of LGALS3BP function can shift ventral progenitors toward a dorsal identity, impacting interneuron specification, and migration in the developing cortex.^90^ De novo mutations in LGALS3BP have been linked to cortical malformations characterized by developmental delay, autism, and microcephaly.^25^ Our in vitro studies validate the function of LGALS3BP in regulating neuronal differentiation in hNSCs by regulating the number of cycling progenitors.

There is an intricate crosstalk between Notch signaling and the extracellular matrix (ECM), facilitating the integration of environmental cues. Notably, Notch directly interacts with various ECM receptors, including MAGP2, EGFL7, CCN3, Thrombospondin2, Syndecan-2, and Galectin-3, as reviewed in LaFoya et al^91^ Additionally, the secreted glycoprotein Reelin plays a critical role in neuronal migration by directly activating Notch-1 and inducing a radial glia phenotype.^92^ Furthermore, the disruption of adherens junctions at the apical surface, achieved through the overexpression of dominant-negative Cadherin, results in impaired Notch signaling and premature neurogenesis.^93^ Notch signaling also acts as a downstream effector of the cell adhesion protein protocadherin 8 (Pcdh8). The ectopic expression of protocadherin 8 in the neocortical pallium represses Notch ligands Delta (Dll1) and Jag1, ultimately leading to premature cell cycle exit.^94^

LSD1 could function to regulate ECM/cell adhesion genes either directly through epigenetic regulation or indirectly via the Notch pathway, which can also modulate the function of ECM/cell adhesion genes.

Thus, we propose that LSD1 plays a critical role in regulating the balance between progenitor cell expansion and neurogenesis in human neural progenitors. It performs this function by repressing Notch signaling and human neural progenitor-enriched ECM/cell adhesion genes. Loss of LSD1 function leads to upregulation of Notch and ECM/Cell adhesion genes, thereby switching to more proliferative divisions, leading to increased stem cell production. A consequence of increased stem cell production could be delayed or decreased neurogenesis or increased apoptosis which in turn results in reduced neuronal output.

## Conclusion

The highly conserved histone lysine demethylating enzyme LSD1 displays differential functions in human and mouse neural development. Our study reveals that LSD1 regulates the expression of novel downstream effector genes with enriched human progenitor expression. In summary, LSD1 plays a crucial role in controlling human neural stem cell differentiation through human-enriched extracellular matrix/cell adhesion and Notch signaling pathway genes.

## Supplementary Material

sxad088_suppl_Supplementary_Figures

sxad088_suppl_Supplementary_Tables

## Data Availability

The ChIP and RNA sequencing data associated with this manuscript have been submitted to GEO with a submission ID: SUB12940500.
