# Supplementary material for: LSD1 Regulates Neurogenesis in Human Neural Stem Cells Through the Repression of Human-Enriched Extracellular Matrix and Cell Adhesion Genes
Source: Stem Cells. Author manuscript; Available in PMC 2024 Feb 9. (PMC10852026; doi:10.1093/stmcls/sxad088)
Supplement: Supplementary tables [file EMS193063-supplement-Supplementary_tables.pdf]

| Genes bound by LSD1 and upregulated upon LSD1 inhibition | | | | | |
|---|---|---|---|---|---|
| **Gene name** | **gene_ID** | **logFC** | **logCPM** | **PValue** | **FDR** |
| COL9A3 | COL9A3_18512 | 2.252724454 | 1.470077739 | 2.07E-75 | 3.85E-72 |
| NOTUM | NOTUM_17664 | 1.967166332 | 0.871318415 | 3.10E-40 | 2.57E-37 |
| STXBP2 | STXBP2_18784 | 1.959330297 | 1.110305438 | 1.58E-43 | 1.68E-40 |
| SBK2 | SBK2_19877 | 1.636687868 | 0.200747496 | 8.04E-19 | 1.05E-16 |
| TRIM47 | TRIM47_17550 | 1.563362905 | 0.044380869 | 5.22E-11 | 2.28E-09 |
| PLEKHG4 | PLEKHG4_16235 | 1.55373778 | -0.1255783 | 7.36E-13 | 4.24E-11 |
| TNNT1 | TNNT1_19854 | 1.522515475 | 1.124488017 | 4.25E-30 | 1.51E-27 |
| C1QL1 | C1QL1_17218 | 1.512803975 | 1.191291528 | 1.51E-27 | 4.24E-25 |
| CLIC6 | CLIC6_20646 | 1.485007798 | -0.267671866 | 4.23E-12 | 2.20E-10 |
| UAP1L1 | UAP1L1_10738 | 1.36628836 | -0.070309155 | 7.46E-11 | 3.15E-09 |
| ADAM33 | ADAM33_18055 | 1.337930141 | -0.418252375 | 2.84E-09 | 9.18E-08 |
| ADM2 | ADM2_20512 | 1.330936265 | 1.725656405 | 1.41E-32 | 5.69E-30 |
| SPINT1 | SPINT1_15091 | 1.313511705 | -0.411281052 | 3.13E-08 | 8.33E-07 |
| CPNE7 | CPNE7_16450 | 1.293534459 | 1.553815181 | 1.61E-25 | 4.06E-23 |
| APOE | APOE_19509 | 1.275947648 | 4.030566212 | 9.53E-112 | 4.74E-108 |
| TMIGD2 | TMIGD2_18700 | 1.264570128 | 0.283520358 | 3.22E-12 | 1.71E-10 |
| YBX2 | YBX2_16622 | 1.203692193 | -0.048871585 | 5.95E-10 | 2.15E-08 |
| BMP8B | BMP8B_576 | 1.198165054 | 3.134347249 | 1.97E-78 | 4.20E-75 |
| HSD11B2 | HSD11B2_16240 | 1.196021962 | -0.286529737 | 1.94E-08 | 5.45E-07 |
| CDC42BPG | CDC42BPG_12315 | 1.194000616 | 1.624194048 | 7.05E-27 | 1.91E-24 |
| EEF1A2 | EEF1A2_18527 | 1.174710412 | 4.385113027 | 5.34E-167 | 7.97E-163 |
| NPTX1 | NPTX1_17623 | 1.140722866 | 0.536212784 | 1.25E-12 | 7.06E-11 |
| PCSK9 | PCSK9_766 | 1.13974747 | 2.021654636 | 2.36E-26 | 6.17E-24 |
| CDH5 | CDH5_16200 | 1.134819554 | 2.294219209 | 3.02E-17 | 3.31E-15 |
| CNFN | CNFN_19443 | 1.134368362 | -0.206906423 | 4.35E-08 | 1.11E-06 |
| FBLN2 | FBLN2_3536 | 1.134158643 | 2.94583462 | 9.91E-57 | 1.48E-53 |
| OCIAD2 | OCIAD2_4798 | 1.092299376 | 2.379023317 | 4.38E-20 | 6.54E-18 |
| TMEM119 | TMEM119_13753 | 1.048654417 | 0.209795906 | 3.76E-09 | 1.19E-07 |
| KISS1R | KISS1R_18589 | 1.038425919 | 0.969534435 | 2.43E-14 | 1.78E-12 |
| KRT80 | KRT80_13346 | 1.03256101 | 0.417292075 | 1.72E-10 | 6.91E-09 |
| BCAN | BCAN_1433 | 1.021559019 | 2.575350921 | 1.04E-40 | 9.71E-38 |
| POU4F1 | POU4F1_14232 | 1.018224979 | 0.444047191 | 9.00E-10 | 3.17E-08 |
| CCR7 | CCR7_17047 | 1.01136745 | 0.354162167 | 2.50E-09 | 8.27E-08 |
| NGFR | NGFR_17300 | 0.986205106 | 1.262054641 | 8.83E-16 | 7.89E-14 |
| CRIP1 | CRIP1_14957 | 0.985861036 | 0.885361555 | 7.29E-13 | 4.22E-11 |
| C11orf35 | C11orf35_11599 | 0.977137147 | 0.451640715 | 2.77E-09 | 9.00E-08 |
| BMP8A | BMP8A_568 | 0.972312144 | 1.925103751 | 3.23E-21 | 5.88E-19 |
| C1QL4 | C1QL4_13299 | 0.966428258 | 1.477678563 | 1.61E-15 | 1.37E-13 |
| NXNL2 | NXNL2_10280 | 0.961430893 | -0.20681627 | 2.33E-06 | 4.09E-05 |

| | | | | | |
|---|---|---|---|---|---|
| SOWAHB | SOWAHB_4942 | 0.961267772 | 0.205244956 | 6.95E-07 | 1.37E-05 |
| JAG2 | JAG2_14950 | 0.95477774 | 1.544817793 | 6.35E-18 | 7.66E-16 |
| SLC4A11 | SLC4A11_18051 | 0.942187782 | -0.273873709 | 1.18E-05 | 0.000173514 |
| SERPINE1 | SERPINE1_7950 | 0.930395923 | 6.70697546 | 6.81E-39 | 5.35E-36 |
| AHNAK2 | AHNAK2_14946 | 0.921371889 | 4.590057675 | 1.04E-99 | 3.09E-96 |
| IGF2 | IGF2_11647 | 0.909151562 | 1.138688922 | 2.63E-13 | 1.64E-11 |
| COL17A1 | COL17A1_11408 | 0.900707608 | 2.869879863 | 1.17E-34 | 6.70E-32 |
| PARVG | PARVG_20463 | 0.876667701 | -0.189190552 | 0.000130107 | 0.001382525 |
| RENBP | RENBP_9143 | 0.874197732 | 1.608969941 | 1.34E-16 | 1.32E-14 |
| GAL | GAL_12445 | 0.870149896 | 1.753492704 | 4.27E-15 | 3.43E-13 |
| COL13A1 | COL13A1_11103 | 0.858802561 | 2.605405246 | 7.28E-20 | 1.05E-17 |
| PLEKHB1 | PLEKHB1_12497 | 0.85793791 | 0.877267776 | 2.20E-08 | 6.03E-07 |
| SIGIRR | SIGIRR_11593 | 0.821862034 | 0.762990927 | 9.93E-09 | 2.93E-07 |
| CLDN6 | CLDN6_15739 | 0.820104401 | 3.61194714 | 3.28E-52 | 4.45E-49 |
| RPRM | RPRM_2951 | 0.814456011 | 0.266541446 | 4.57E-06 | 7.42E-05 |
| C8orf47 | C8orf47_9685 | 0.808420455 | 0.846098896 | 2.08E-07 | 4.73E-06 |
| FAM83G | FAM83G_16791 | 0.787813985 | 2.257815392 | 2.31E-17 | 2.61E-15 |
| VWA1 | VWA1_47 | 0.78226991 | 2.17455664 | 1.34E-18 | 1.69E-16 |
| RP11-267C16.1 | RP11-267C16.1_17891 | 0.781959209 | 0.813728853 | 7.31E-08 | 1.80E-06 |
| KIF1A | KIF1A_3442 | 0.777468942 | 5.520604974 | 8.22E-115 | 6.13E-111 |
| SHC2 | SHC2_18566 | 0.769083382 | 3.332645406 | 3.04E-32 | 1.20E-29 |
| GDF15 | GDF15_19092 | 0.762571817 | 2.330640935 | 1.77E-16 | 1.70E-14 |
| GPR157 | GPR157_131 | 0.762556419 | -0.027574436 | 5.17E-05 | 0.000627066 |
| KIAA1211L | KIAA1211L_2714 | 0.762305312 | 0.496308078 | 1.08E-06 | 2.05E-05 |
| ADAMTS14 | ADAMTS14_11116 | 0.761354508 | 2.715468228 | 1.65E-16 | 1.61E-14 |
| CELF5 | CELF5_18669 | 0.756675742 | 0.972178003 | 8.51E-09 | 2.54E-07 |
| STEAP3 | STEAP3_2831 | 0.755662465 | 0.805140988 | 6.20E-08 | 1.55E-06 |
| CATSPER3 | CATSPER3_5871 | 0.755386756 | 2.364280356 | 0.000776799 | 0.006210644 |
| STC2 | STC2_6161 | 0.753733555 | 2.232863295 | 5.48E-17 | 5.84E-15 |
| ANO1 | ANO1_12459 | 0.751140076 | 0.53622661 | 8.80E-06 | 0.000135223 |
| RLTPR | RLTPR_16245 | 0.751089853 | 0.961863497 | 1.60E-08 | 4.55E-07 |
| PHLDA2 | PHLDA2_11664 | 0.750964398 | 2.952423612 | 2.44E-23 | 5.13E-21 |
| RP11-43F13.3 | RP11-43F13.3_5384 | 0.746577739 | 0.629325168 | 2.15E-06 | 3.80E-05 |
| TBX10 | TBX10_12432 | 0.744351737 | 0.921186711 | 2.83E-07 | 6.15E-06 |
| MMP23A | MMP23A_57 | 0.734998282 | 0.525513164 | 3.28E-05 | 0.000424221 |
| CDCP1 | CDCP1_3698 | 0.734210878 | 3.424264448 | 5.87E-17 | 6.17E-15 |
| MAP1LC3C | MAP1LC3C_2072 | 0.728089439 | 2.369919479 | 1.11E-18 | 1.41E-16 |
| TIE1 | TIE1_625 | 0.725537511 | 0.150585092 | 7.60E-05 | 0.000875892 |
| DLK1 | DLK1_14899 | 0.723243404 | 3.597232549 | 7.97E-33 | 3.30E-30 |
| PTPN3 | PTPN3_10410 | 0.72081299 | 0.813956584 | 1.27E-06 | 2.37E-05 |
| COL9A2 | COL9A2_586 | 0.720246409 | 1.702101557 | 3.99E-11 | 1.79E-09 |

| | | | | | |
|---|---|---|---|---|---|
| RPP25 | RPP25_15389 | 0.719644291 | 1.84691502 | 1.15E-12 | 6.51E-11 |
| TLR6 | TLR6_4743 | 0.712844747 | -0.086886628 | 0.000520782 | 0.004478121 |
| CHRNB1 | CHRNB1_16637 | 0.711571669 | 2.103205956 | 1.73E-10 | 6.94E-09 |
| CYBA | CYBA_16428 | 0.710442495 | 0.064277197 | 0.000124666 | 0.001335168 |
| NFATC2 | NFATC2_18431 | 0.708887432 | 2.471077131 | 6.05E-19 | 8.06E-17 |
| GPR123 | GPR123_11556 | 0.7037862 | 1.469119761 | 3.40E-06 | 5.71E-05 |
| LAMP3 | LAMP3_4434 | 0.702326851 | 3.750499331 | 5.07E-35 | 3.15E-32 |
| F12 | F12_6199 | 0.69946661 | 0.12241551 | 9.68E-05 | 0.001075614 |
| CD9 | CD9_12975 | 0.692374244 | 1.440305479 | 3.38E-10 | 1.27E-08 |
| KCNMB1 | KCNMB1_6140 | 0.684026438 | 1.667177444 | 3.68E-11 | 1.66E-09 |
| RASEF | RASEF_10249 | 0.679083329 | 1.227286997 | 3.08E-07 | 6.64E-06 |
| TMEM130 | TMEM130_7874 | 0.678675224 | 1.755198709 | 1.05E-11 | 5.15E-10 |
| HMOX1 | HMOX1_20306 | 0.672210058 | 0.093555773 | 0.00019054 | 0.001911988 |
| SCG2 | SCG2_3319 | 0.668558255 | 0.775386721 | 8.10E-06 | 0.000126466 |
| PLA2G3 | PLA2G3_20279 | 0.665711728 | 3.054253116 | 1.52E-24 | 3.61E-22 |
| SNAI1 | SNAI1_18419 | 0.66559922 | 0.649269454 | 1.72E-05 | 0.000241187 |
| SLC5A10 | SLC5A10_16790 | 0.664801126 | 2.769023789 | 1.52E-16 | 1.49E-14 |
| MAOA | MAOA_8505 | 0.662645956 | 0.04909905 | 0.000570836 | 0.004822367 |
| FSTL3 | FSTL3_18577 | 0.657459502 | 3.135556263 | 3.77E-21 | 6.78E-19 |
| PIK3R5 | PIK3R5_16690 | 0.656038798 | 0.635794219 | 1.30E-05 | 0.000189792 |
| NKX6-2 | NKX6-2_11554 | 0.654360113 | 4.221640708 | 6.39E-24 | 1.42E-21 |
| SPP1 | SPP1_5000 | 0.648372598 | 2.378603881 | 5.00E-12 | 2.55E-10 |
| INHBA | INHBA_7584 | 0.647551025 | 5.145647539 | 8.42E-21 | 1.43E-18 |
| SCARF1 | SCARF1_16490 | 0.646002059 | 0.868647104 | 2.42E-06 | 4.23E-05 |
| TMBIM1 | TMBIM1_3261 | 0.644858249 | 1.323401971 | 4.33E-08 | 1.11E-06 |
| PGF | PGF_14749 | 0.644636543 | 0.321323036 | 0.000347951 | 0.003188629 |
| CYP1B1 | CYP1B1_2357 | 0.641036118 | 4.52712616 | 8.73E-17 | 8.89E-15 |
| P2RY2 | P2RY2_12491 | 0.640224263 | -0.017187611 | 0.00080016 | 0.006358575 |
| ELN | ELN_7732 | 0.639834302 | 2.402031837 | 2.87E-10 | 1.09E-08 |
| VGF | VGF_7952 | 0.638628281 | 2.869371964 | 9.41E-14 | 6.44E-12 |
| EHD2 | EHD2_19590 | 0.638163627 | 0.240792335 | 0.000182082 | 0.001841682 |
| ADAP2 | ADAP2_16902 | 0.636107378 | 1.772036298 | 3.59E-08 | 9.44E-07 |
| CYGB | CYGB_17571 | 0.633962833 | 1.40181403 | 1.00E-06 | 1.93E-05 |
| MAP3K7CL | MAP3K7CL_20571 | 0.632381343 | 2.183975314 | 1.66E-09 | 5.63E-08 |
| FAM110C | FAM110C_2151 | 0.629513858 | 1.417250495 | 1.76E-08 | 4.99E-07 |
| FAM20C | FAM20C_7346 | 0.626475488 | 2.564342107 | 3.68E-13 | 2.24E-11 |
| LRRK1 | LRRK1_15583 | 0.625486989 | 0.926757318 | 1.51E-05 | 0.000216278 |
| PLP2 | PLP2_8581 | 0.624300065 | 0.362131859 | 0.000127265 | 0.001356194 |
| S100Z | S100Z_5656 | 0.621657316 | 0.173694754 | 0.000932399 | 0.007237493 |
| STC1 | STC1_9368 | 0.617625226 | 0.098476831 | 0.001321528 | 0.009712252 |
| CHODL | CHODL_20550 | 0.61628792 | 4.133532317 | 9.71E-34 | 4.99E-31 |

| | | | | | |
|---|---|---|---|---|---|
| NR1H4 | NR1H4_13696 | 0.61615692 | 0.304945863 | 0.000991676 | 0.007618337 |
| GRIK5 | GRIK5_19431 | 0.615996475 | 1.27952931 | 1.47E-06 | 2.71E-05 |
| ID1 | ID1_18192 | 0.61446666 | 2.129958556 | 7.06E-07 | 1.39E-05 |
| ZYG11A | ZYG11A_732 | 0.613802899 | 0.575009833 | 0.000131717 | 0.001397643 |
| CPT1A | CPT1A_12447 | 0.612910512 | 2.247843676 | 3.13E-11 | 1.43E-09 |
| C6orf141 | C6orf141_6853 | 0.610636795 | 0.044372353 | 0.000942995 | 0.007300746 |
| IL11 | IL11_19867 | 0.610618253 | 2.913168557 | 2.16E-10 | 8.45E-09 |
| TGFBI | TGFBI_5883 | 0.608947969 | 2.111418843 | 3.46E-10 | 1.30E-08 |
| NRK | NRK_8854 | 0.603716436 | 1.900619101 | 1.24E-08 | 3.60E-07 |
| MBP | MBP_17976 | 0.59954089 | 2.396624294 | 4.49E-13 | 2.71E-11 |
| MLPH | MLPH_3407 | 0.599202928 | 4.085907821 | 1.05E-26 | 2.80E-24 |
| CACNA1B | CACNA1B_10769 | 0.5944191 | 1.386169066 | 2.13E-07 | 4.81E-06 |
| LLGL2 | LLGL2_17540 | 0.594204295 | 2.85135148 | 4.27E-19 | 5.74E-17 |
| OCA2 | OCA2_15000 | 0.593405166 | 2.119759273 | 1.98E-10 | 7.87E-09 |
| OTOGL | OTOGL_13618 | 0.592107751 | 2.509761449 | 1.26E-14 | 9.56E-13 |
| NPPB | NPPB_170 | 0.592001619 | 3.314003656 | 7.45E-11 | 3.15E-09 |
| TP73 | TP73_87 | 0.59169333 | 1.225219282 | 1.36E-06 | 2.54E-05 |
| TERT | TERT_5389 | 0.589466175 | 1.617299604 | 1.75E-06 | 3.17E-05 |
| DBNDD1 | DBNDD1_16465 | 0.585368043 | 3.168403755 | 8.83E-19 | 1.15E-16 |
| ENG | ENG_10557 | 0.584597665 | 0.868484297 | 3.01E-05 | 0.00039569 |
| SPOCD1 | SPOCD1_465 | 0.583975015 | 0.079119757 | 0.00240125 | 0.015774658 |
| PARM1 | PARM1_4921 | 0.583104449 | 3.409993883 | 2.39E-15 | 1.96E-13 |
| ACHE | ACHE_7945 | 0.582721773 | 2.752630784 | 2.48E-08 | 6.70E-07 |
| NOS3 | NOS3_8278 | 0.581734647 | 0.592029701 | 0.000208339 | 0.002061147 |
| VAMP8 | VAMP8_2608 | 0.579131897 | 0.921425783 | 2.19E-05 | 0.000298228 |
| HOOK1 | HOOK1_782 | 0.574371416 | 2.634977393 | 2.71E-14 | 1.97E-12 |
| IQSEC2 | IQSEC2_8627 | 0.569667726 | 1.523603599 | 9.99E-08 | 2.38E-06 |
| INSM1 | INSM1_18137 | 0.56936428 | 2.848835661 | 5.76E-15 | 4.52E-13 |
| CMYA5 | CMYA5_5675 | 0.56487001 | 0.117401375 | 0.002542119 | 0.016508113 |
| LPIN3 | LPIN3_18317 | 0.564763026 | 1.678396118 | 1.18E-06 | 2.23E-05 |
| KCNG3 | KCNG3_2379 | 0.563668437 | -0.188994169 | 0.005904704 | 0.031974083 |
| PRCD | PRCD_17572 | 0.563473128 | 1.438441727 | 1.36E-05 | 0.000196436 |
| MPP4 | MPP4_3174 | 0.562874536 | -0.075707514 | 0.003999828 | 0.023623685 |
| TRIM38 | TRIM38_6399 | 0.562304835 | 0.735467006 | 0.000122265 | 0.001315593 |
| HMHA1 | HMHA1_18596 | 0.561473776 | 3.977008758 | 6.68E-29 | 2.26E-26 |
| ARSI | ARSI_6050 | 0.558838277 | 0.772136926 | 0.000152066 | 0.001580956 |
| ASS1 | ASS1_10618 | 0.552315102 | 1.236077069 | 9.24E-06 | 0.000140452 |
| LGALS1 | LGALS1_20345 | 0.551952628 | 5.888729923 | 2.95E-73 | 4.89E-70 |
| PTPRU | PTPRU_449 | 0.550980305 | 4.287734041 | 1.65E-33 | 7.70E-31 |
| KRTAP5-AS1 | KRTAP5-AS1_11632 | 0.549544784 | 0.924244908 | 5.11E-05 | 0.000620496 |
| RGS9 | RGS9_17457 | 0.549029477 | 2.757644546 | 5.20E-15 | 4.10E-13 |

| | | | | | |
|---|---|---|---|---|---|
| AQP7 | AQP7_10048 | 0.545794473 | 0.11751802 | 0.002424203 | 0.015893512 |
| PLAUR | PLAUR_19473 | 0.539106192 | 3.385335915 | 1.26E-13 | 8.44E-12 |
| EFNA1 | EFNA1_1376 | 0.53816112 | -0.103191474 | 0.007678108 | 0.039431909 |
| BIRC3 | BIRC3_12644 | 0.536518391 | 0.064221339 | 0.003528246 | 0.021371436 |
| S100A4 | S100A4_1327 | 0.534382898 | 0.529176944 | 0.000780274 | 0.006225087 |
| BATF3 | BATF3_1901 | 0.533761434 | 2.899890731 | 2.98E-15 | 2.40E-13 |
| NEFH | NEFH_20245 | 0.531746075 | 4.206860868 | 6.95E-35 | 4.15E-32 |
| GAD1 | GAD1_3024 | 0.530113891 | 0.629242389 | 0.000273057 | 0.002599704 |
| C19orf66 | C19orf66_18848 | 0.529950115 | 0.223126371 | 0.003564838 | 0.021540633 |
| HSPA12B | HSPA12B_18057 | 0.528939988 | -0.108881538 | 0.006451597 | 0.034239872 |
| FAM189A2 | FAM189A2_10208 | 0.526226233 | 1.04680692 | 0.000119993 | 0.001295352 |
| EMP1 | EMP1_13121 | 0.525591248 | 5.526057623 | 1.00E-37 | 6.80E-35 |
| RPS6KL1 | RPS6KL1_14748 | 0.524219915 | 2.319235783 | 3.26E-10 | 1.23E-08 |
| KCNJ2 | KCNJ2_17485 | 0.524125789 | 2.602989664 | 1.28E-11 | 6.18E-10 |
| KDR | KDR_4816 | 0.524029504 | 5.187602321 | 9.95E-14 | 6.78E-12 |
| LGALS3BP | LGALS3BP_17603 | 0.523739961 | 4.719534619 | 7.67E-34 | 4.09E-31 |
| CEND1 | CEND1_11613 | 0.523626306 | 1.49629343 | 1.22E-06 | 2.29E-05 |
| AIM1L | AIM1L_390 | 0.522052153 | 1.91605016 | 5.30E-06 | 8.50E-05 |
| ANKRD45 | ANKRD45_1639 | 0.521630776 | 3.437815572 | 2.79E-20 | 4.27E-18 |
| PEAR1 | PEAR1_1444 | 0.521473804 | 2.521826522 | 5.58E-11 | 2.43E-09 |
| RRAD | RRAD_16215 | 0.518082172 | 2.73921198 | 1.27E-12 | 7.11E-11 |
| CD6 | CD6_12201 | 0.516901192 | 0.455519949 | 0.001903361 | 0.013049741 |
| ALDOC | ALDOC_16858 | 0.516616429 | 0.474072469 | 0.001808783 | 0.012527963 |
| ITGA3 | ITGA3_17311 | 0.516061959 | 4.509329331 | 9.73E-29 | 3.23E-26 |
| ERAP2 | ERAP2_5732 | 0.51301759 | 0.862883286 | 0.000602397 | 0.005048966 |
| SLC16A6 | SLC16A6_17474 | 0.512712043 | 1.610570841 | 1.41E-06 | 2.61E-05 |
| SNCG | SNCG_11212 | 0.510638717 | 1.126944107 | 3.12E-05 | 0.00040754 |
| ADAMTS16 | ADAMTS16_5400 | 0.509865567 | 3.936504744 | 1.66E-20 | 2.67E-18 |
| EHBP1L1 | EHBP1L1_12350 | 0.509677518 | 3.447972068 | 1.40E-13 | 9.30E-12 |
| LAMC2 | LAMC2_1700 | 0.507442322 | 0.141338284 | 0.006111567 | 0.032845269 |
| OLFML3 | OLFML3_1081 | 0.506659646 | 2.970164238 | 9.79E-15 | 7.61E-13 |
| CACNG6 | CACNG6_19809 | 0.505548522 | 2.336503851 | 4.04E-09 | 1.27E-07 |
| S100B | S100B_20780 | 0.505497455 | 0.240694157 | 0.003091635 | 0.019242431 |
| ENO2 | ENO2_13010 | 0.505350072 | 5.363849829 | 1.45E-33 | 6.99E-31 |
| SRRM3 | SRRM3_7773 | 0.505201434 | 1.05698543 | 0.000728738 | 0.005902307 |
| KRT8 | KRT8_13372 | 0.499879306 | 2.871180124 | 1.68E-13 | 1.09E-11 |
| LOXL4 | LOXL4_11326 | 0.49741557 | 0.831362831 | 0.000543471 | 0.004643786 |
| EMILIN2 | EMILIN2_17712 | 0.496272323 | 5.057129677 | 1.95E-27 | 5.38E-25 |
| SYNPO | SYNPO_6055 | 0.495264678 | 4.084313878 | 1.60E-11 | 7.58E-10 |
| FGFBP3 | FGFBP3_11256 | 0.494584726 | 8.637795464 | 9.41E-79 | 2.34E-75 |
| BHLHE40 | BHLHE40_3476 | 0.493711413 | 4.454788211 | 2.81E-20 | 4.27E-18 |

| | | | | | |
|---|---|---|---|---|---|
| CD34 | CD34_1867 | 0.490945976 | 1.709132734 | 6.47E-05 | 0.000760904 |
| CREB3L1 | CREB3L1_12004 | 0.490842787 | 1.831347872 | 4.14E-07 | 8.68E-06 |
| LIF | LIF_20255 | 0.488856275 | 4.763040652 | 5.31E-16 | 4.89E-14 |
| ARHGDIB | ARHGDIB_13135 | 0.488791066 | 1.138536463 | 0.000359281 | 0.003276354 |
| EMILIN1 | EMILIN1_2276 | 0.488122665 | 2.238292331 | 2.10E-08 | 5.78E-07 |
| CD97 | CD97_18981 | 0.486936892 | 0.99835888 | 0.000757193 | 0.006083252 |
| LAYN | LAYN_12699 | 0.486877893 | 2.344084472 | 3.06E-07 | 6.62E-06 |
| MYOM1 | MYOM1_17714 | 0.485656167 | 1.141029961 | 8.18E-05 | 0.000929839 |
| HTR1D | HTR1D_331 | 0.483695362 | 0.390136296 | 0.003788683 | 0.022591273 |
| CRISPLD2 | CRISPLD2_16399 | 0.483636917 | 0.093689045 | 0.009197462 | 0.04534598 |
| BEST3 | BEST3_13580 | 0.482784094 | 2.485555021 | 1.34E-08 | 3.88E-07 |
| SNTG2 | SNTG2_2156 | 0.482382206 | 1.937854162 | 3.94E-06 | 6.54E-05 |
| RAP1GAP2 | RAP1GAP2_16517 | 0.479744991 | 2.776762685 | 7.06E-07 | 1.39E-05 |
| KCNJ4 | KCNJ4_20364 | 0.479075312 | 2.154027082 | 5.93E-08 | 1.49E-06 |
| CEBPD | CEBPD_9490 | 0.479016741 | 2.040494575 | 5.73E-07 | 1.16E-05 |
| GDNF | GDNF_5488 | 0.478408751 | 5.975917654 | 1.94E-31 | 7.41E-29 |
| GRAP | GRAP_16792 | 0.476330931 | 2.637742288 | 7.11E-08 | 1.75E-06 |
| ABCC6P1 | ABCC6P1_15870 | 0.475603562 | 0.366388459 | 0.00543371 | 0.030079968 |
| ITK | ITK_6093 | 0.475572426 | 3.594792412 | 5.62E-09 | 1.74E-07 |
| SLCO4C1 | SLCO4C1_5742 | 0.4728563 | 0.200853396 | 0.006912675 | 0.036224164 |
| ODF3B | ODF3B_20518 | 0.471387284 | 0.401903435 | 0.003525864 | 0.021365705 |
| NT5E | NT5E_6969 | 0.470755265 | 3.848579325 | 1.32E-11 | 6.34E-10 |
| ABCA4 | ABCA4_942 | 0.469689504 | 0.474261544 | 0.002383682 | 0.015677593 |
| STMN4 | STMN4_9383 | 0.46831067 | 1.103037869 | 0.000488782 | 0.004260435 |
| CLDN1 | CLDN1_4500 | 0.466094508 | 3.504621515 | 2.39E-14 | 1.76E-12 |
| DNAH5 | DNAH5_5423 | 0.464982202 | 3.117016213 | 1.64E-12 | 8.97E-11 |
| HERC6 | HERC6_5004 | 0.464001651 | 1.4925426 | 0.000301243 | 0.002815944 |
| GREM1 | GREM1_15033 | 0.463882184 | 6.077733679 | 1.71E-18 | 2.15E-16 |
| C12orf39 | C12orf39_13162 | 0.463107482 | 1.962862428 | 1.85E-06 | 3.33E-05 |
| ID3 | ID3_338 | 0.462982554 | 4.258585223 | 3.34E-11 | 1.51E-09 |
| SLFN13 | SLFN13_16944 | 0.462865766 | 4.303299503 | 3.71E-23 | 7.69E-21 |
| CLDN4 | CLDN4_7729 | 0.462848124 | 1.725933409 | 8.19E-05 | 0.000930277 |
| SBSPON | SBSPON_9587 | 0.462355491 | 1.021756179 | 0.000450785 | 0.003984163 |
| KRT18 | KRT18_13373 | 0.462206162 | 3.441385006 | 3.12E-14 | 2.26E-12 |
| TMEM102 | TMEM102_16635 | 0.458799126 | 1.031668134 | 0.000407204 | 0.003643511 |
| CTHRC1 | CTHRC1_9716 | 0.458783423 | 0.317153569 | 0.006661915 | 0.035169534 |
| NPAS1 | NPAS1_19574 | 0.456991718 | 1.066657455 | 0.000299823 | 0.002809211 |
| EMILIN3 | EMILIN3_18318 | 0.455512542 | 3.704264511 | 5.33E-18 | 6.52E-16 |
| CD74 | CD74_6052 | 0.45495748 | 1.016558972 | 0.001463661 | 0.010538786 |
| NPL | NPL_1696 | 0.454207806 | 1.593636318 | 2.41E-05 | 0.000324396 |
| FOXL1 | FOXL1_16412 | 0.454100665 | 0.30472496 | 0.007698814 | 0.039483881 |

| | | | | | |
|---|---|---|---|---|---|
| PLEKHG6 | PLEKHG6_12977 | 0.453941823 | 1.593709713 | 1.90E-05 | 0.000264015 |
| RAPGEF3 | RAPGEF3_13258 | 0.453738782 | 1.564194262 | 2.08E-05 | 0.000286077 |
| TAGLN2 | TAGLN2_1498 | 0.453236986 | 5.727809572 | 8.76E-17 | 8.89E-15 |
| MLK4 | MLK4_2034 | 0.452736521 | 0.921420742 | 0.000690711 | 0.005652613 |
| NECAB1 | NECAB1_9646 | 0.452566943 | 2.034179064 | 5.01E-07 | 1.02E-05 |
| COL18A1 | COL18A1_20764 | 0.448948839 | 7.215184743 | 1.56E-40 | 1.37E-37 |
| SPINT2 | SPINT2_19312 | 0.448858361 | 2.493225012 | 1.65E-05 | 0.000233191 |
| SPRY4 | SPRY4_5998 | 0.448197501 | 4.857131935 | 1.12E-16 | 1.12E-14 |
| EPAS1 | EPAS1_2400 | 0.447484367 | 0.539681327 | 0.004215669 | 0.024683503 |
| PAPPA | PAPPA_10466 | 0.44653378 | 6.497673911 | 2.39E-21 | 4.41E-19 |
| COL6A2 | COL6A2_20770 | 0.445714974 | 4.130681056 | 3.92E-22 | 7.79E-20 |
| TTBK1 | TTBK1_6799 | 0.444325217 | 3.473523354 | 1.90E-15 | 1.59E-13 |
| LTBP2 | LTBP2_14742 | 0.443977861 | 2.507651686 | 3.45E-07 | 7.40E-06 |
| SERPINB9 | SERPINB9_6281 | 0.441980747 | 2.365998126 | 7.53E-07 | 1.47E-05 |
| IKZF1 | IKZF1_7643 | 0.441529691 | 0.921432307 | 0.001021107 | 0.007820273 |
| GNG3 | GNG3_12252 | 0.439398006 | 2.345057587 | 3.71E-07 | 7.87E-06 |
| SYNGR3 | SYNGR3_15694 | 0.438711059 | 2.876373807 | 3.95E-10 | 1.47E-08 |
| F2RL1 | F2RL1_5655 | 0.437592239 | 4.303519894 | 1.76E-10 | 7.04E-09 |
| WNT3A | WNT3A_1985 | 0.436870662 | 3.098774128 | 6.51E-09 | 1.99E-07 |
| GPR176 | GPR176_15065 | 0.43647816 | 2.043320855 | 3.06E-05 | 0.000401537 |
| HIC1 | HIC1_16506 | 0.43549345 | 1.117435895 | 0.000660315 | 0.005445682 |
| OCSTAMP | OCSTAMP_18398 | 0.434766843 | 2.435794769 | 8.84E-08 | 2.14E-06 |
| ELTD1 | ELTD1_868 | 0.433305833 | 2.634408921 | 2.70E-05 | 0.000358396 |
| ETV4 | ETV4_17176 | 0.432222964 | 2.875868215 | 9.94E-06 | 0.000149541 |
| DLL3 | DLL3_19357 | 0.431628415 | 0.808142014 | 0.00332245 | 0.020332777 |
| ENPP4 | ENPP4_6833 | 0.431184398 | 2.805457947 | 4.59E-08 | 1.17E-06 |
| DEPDC7 | DEPDC7_11949 | 0.430927437 | 0.735368144 | 0.00353785 | 0.021412242 |
| MFRP | MFRP_12802 | 0.429719094 | 1.382092193 | 0.000163551 | 0.001681611 |
| C1QTNF5 | C1QTNF5_12801 | 0.429718317 | 1.382092193 | 0.000163351 | 0.001680711 |
| MORN3 | MORN3_13883 | 0.427836929 | 0.578313851 | 0.008251459 | 0.041730003 |
| IL1A | IL1A_2802 | 0.427219604 | 1.466534364 | 0.000467062 | 0.004113398 |
| NUDT14 | NUDT14_14951 | 0.426930656 | 3.524409852 | 8.24E-13 | 4.73E-11 |
| TGFA | TGFA_2516 | 0.426366316 | 1.813988619 | 0.000192651 | 0.001930134 |
| PKP2 | PKP2_13223 | 0.424878413 | 4.647898072 | 5.66E-24 | 1.30E-21 |
| PLCG2 | PLCG2_16375 | 0.42473938 | 1.071596076 | 0.000851719 | 0.006719621 |
| CFLAR | CFLAR_3166 | 0.423127993 | 4.731253129 | 1.17E-11 | 5.67E-10 |
| MUC12 | MUC12_7947 | 0.422908453 | 1.150337108 | 0.000965508 | 0.007440293 |
| PLA2G4C | PLA2G4C_19600 | 0.422900215 | 2.453445835 | 2.48E-07 | 5.50E-06 |
| PGPEP1 | PGPEP1_19091 | 0.422850542 | 5.670432969 | 4.49E-30 | 1.56E-27 |
| KIAA1755 | KIAA1755_18299 | 0.422408936 | 2.200716491 | 1.94E-05 | 0.000268632 |
| ITGA2B | ITGA2B_17203 | 0.421922415 | 0.716563188 | 0.010286489 | 0.049431522 |

| | | | | | |
|---|---|---|---|---|---|
| JOSD2 | JOSD2_19706 | 0.421520074 | 0.760049616 | 0.003169814 | 0.019666633 |
| S100A16 | S100A16_1330 | 0.421150331 | 4.052340125 | 1.04E-14 | 7.96E-13 |
| FAM129A | FAM129A_1711 | 0.421012427 | 4.00057817 | 2.18E-16 | 2.06E-14 |
| MROH6 | MROH6_9853 | 0.420827421 | 1.218578175 | 0.001048216 | 0.008007342 |
| S100A10 | S100A10_1271 | 0.418970629 | 3.520077128 | 5.97E-10 | 2.15E-08 |
| RHBDF2 | RHBDF2_17570 | 0.418853735 | 2.234971451 | 2.03E-06 | 3.62E-05 |
| ROBO3 | ROBO3_12863 | 0.41884766 | 3.161883994 | 2.35E-10 | 9.13E-09 |
| MRGPRF | MRGPRF_12451 | 0.418117666 | 0.876917088 | 0.005609274 | 0.030732558 |
| BEX2 | BEX2_8831 | 0.417791203 | 3.243747944 | 2.18E-10 | 8.52E-09 |
| SHISA2 | SHISA2_14024 | 0.41685866 | 1.81681425 | 0.000777576 | 0.006211806 |
| C10orf55 | C10orf55_11159 | 0.416082333 | 4.766855281 | 5.81E-17 | 6.15E-15 |
| SERPINB8 | SERPINB8_17947 | 0.41572661 | 3.742201366 | 6.37E-08 | 1.58E-06 |
| PLAU | PLAU_11158 | 0.415474877 | 4.76247118 | 9.91E-17 | 9.92E-15 |
| NNMT | NNMT_12731 | 0.412965097 | 2.235377824 | 4.73E-05 | 0.000581847 |
| ELAVL3 | ELAVL3_18897 | 0.412954566 | 6.064462354 | 3.96E-36 | 2.57E-33 |
| TNC | TNC_10464 | 0.412900049 | 6.176721052 | 3.54E-14 | 2.54E-12 |
| HAPLN3 | HAPLN3_15517 | 0.412112353 | 1.44048693 | 0.000650808 | 0.005391116 |
| ADAM12 | ADAM12_11532 | 0.411656146 | 4.257242655 | 1.58E-07 | 3.66E-06 |
| KCNQ4 | KCNQ4_595 | 0.409563625 | 1.136515015 | 0.001575505 | 0.011155651 |
| NCR3LG1 | NCR3LG1_11872 | 0.409528672 | 5.22526638 | 4.02E-25 | 9.83E-23 |
| CLCNKB | CLCNKB_236 | 0.409463091 | 0.967122488 | 0.002215458 | 0.014768732 |
| SERTM1 | SERTM1_14078 | 0.408686007 | 1.356297748 | 0.000395571 | 0.003561574 |
| KIF17 | KIF17_303 | 0.408357601 | 2.921663723 | 8.14E-09 | 2.45E-07 |
| SARDH | SARDH_10671 | 0.407462894 | 1.607459921 | 0.000139242 | 0.001464986 |
| CHST6 | CHST6_16348 | 0.405873725 | 2.332902549 | 6.32E-05 | 0.000747079 |
| CTD-2008L17.2 | CTD-2008L17.2_17901 | 0.404635678 | 1.214539697 | 0.004242068 | 0.024818594 |
| LPPR5 | LPPR5_957 | 0.403584799 | 1.682983059 | 0.000367531 | 0.003343415 |
| SLC19A1 | SLC19A1_20766 | 0.402651969 | 6.530961897 | 1.80E-38 | 1.35E-35 |
| RAB7L1 | RAB7L1_1834 | 0.402320526 | 1.202837572 | 0.001107129 | 0.008364493 |
| NLRC5 | NLRC5_16161 | 0.401571321 | 2.036889217 | 0.000104419 | 0.001147143 |
| MIR137HG | MIR137HG_955 | 0.401503266 | 1.283817261 | 0.000964127 | 0.007433496 |
| KCNU1 | KCNU1_9427 | 0.400931077 | 1.639156635 | 0.000104202 | 0.001145607 |
| INPP5D | INPP5D_3381 | 0.400575056 | 1.220966541 | 0.003007645 | 0.018805974 |
| NRG3 | NRG3_11197 | 0.399890858 | 4.017760315 | 4.73E-13 | 2.83E-11 |
| FST | FST_5532 | 0.399729786 | 5.718129932 | 1.55E-12 | 8.48E-11 |
| USP18 | USP18_20078 | 0.398488147 | 1.4018134 | 0.000342687 | 0.003146183 |
| DNAJC6 | DNAJC6_806 | 0.398157337 | 2.641114322 | 1.22E-06 | 2.29E-05 |
| FABP3 | FABP3_458 | 0.395801302 | 4.484769946 | 3.28E-17 | 3.57E-15 |
| HPSE | HPSE_4976 | 0.395461988 | 0.655788326 | 0.007023805 | 0.03670338 |
| GRAPL | GRAPL_16797 | 0.394703259 | 2.373143876 | 9.45E-06 | 0.000142974 |
| KCNK5 | KCNK5_6742 | 0.393899005 | 1.071511461 | 0.002691838 | 0.017258072 |

| | | | | | |
|---|---|---|---|---|---|
| HSD17B14 | HSD17B14_19630 | 0.393869299 | 3.499394841 | 1.08E-09 | 3.73E-08 |
| COL1A1 | COL1A1_17317 | 0.393763478 | 9.30996682 | 1.71E-20 | 2.72E-18 |
| ICAM5 | ICAM5_18858 | 0.393086084 | 0.639399982 | 0.009443746 | 0.046223596 |
| TRANK1 | TRANK1_3618 | 0.392275681 | 1.398150036 | 0.001123023 | 0.008461985 |
| HAP1 | HAP1_17109 | 0.391904557 | 2.546735292 | 2.08E-06 | 3.71E-05 |
| PLCB2 | PLCB2_15072 | 0.389879845 | 4.609246005 | 8.58E-20 | 1.23E-17 |
| FAM179A | FAM179A_2316 | 0.389745265 | 1.08640009 | 0.002586316 | 0.016696343 |
| IL32 | IL32_15747 | 0.389533288 | 4.478181504 | 2.68E-11 | 1.24E-09 |
| HES6 | HES6_3420 | 0.389410456 | 4.837214006 | 7.02E-18 | 8.31E-16 |
| GABRA5 | GABRA5_14998 | 0.388582843 | 3.32394531 | 2.09E-11 | 9.81E-10 |
| ABCA3 | ABCA3_15713 | 0.388504608 | 5.556120432 | 8.98E-28 | 2.58E-25 |
| MICAL2 | MICAL2_11846 | 0.387262124 | 5.095391983 | 5.43E-13 | 3.20E-11 |
| CSRNP1 | CSRNP1_3644 | 0.387161702 | 4.292959559 | 2.51E-17 | 2.82E-15 |
| SPTB | SPTB_14662 | 0.386182737 | 3.155439261 | 1.61E-10 | 6.52E-09 |
| CEBPB | CEBPB_18422 | 0.385556581 | 2.054463577 | 0.000142347 | 0.001493438 |
| ALPL | ALPL_312 | 0.385367004 | 3.695850354 | 2.86E-10 | 1.09E-08 |
| KIAA1239 | KIAA1239_4735 | 0.384977506 | 2.881862998 | 5.25E-07 | 1.07E-05 |
| MFAP3L | MFAP3L_5289 | 0.384661813 | 3.798288912 | 4.93E-08 | 1.25E-06 |
| PTPRH | PTPRH_19858 | 0.384585446 | 1.366105979 | 0.000720367 | 0.005844021 |
| SNAI3 | SNAI3_16431 | 0.383414898 | 1.088766726 | 0.003443537 | 0.02096903 |
| TMEM71 | TMEM71_9802 | 0.383379543 | 2.826232943 | 5.31E-08 | 1.34E-06 |
| TCERG1L | TCERG1L_11544 | 0.382691681 | 3.20119098 | 9.16E-10 | 3.20E-08 |
| SH2D5 | SH2D5_304 | 0.382038007 | 2.493977499 | 8.45E-05 | 0.000955553 |
| ANO4 | ANO4_13698 | 0.381254517 | 4.545680997 | 3.02E-12 | 1.61E-10 |
| TAP2 | TAP2_6640 | 0.380558227 | 1.753942244 | 0.000194791 | 0.001949084 |
| FURIN | FURIN_15546 | 0.379934957 | 4.762162619 | 6.27E-24 | 1.42E-21 |
| PTPRN | PTPRN_3295 | 0.379505578 | 1.395846828 | 0.000836157 | 0.006607321 |
| TOR4A | TOR4A_10755 | 0.378404211 | 1.329672412 | 0.000957834 | 0.007392612 |
| RAPGEF4 | RAPGEF4_3038 | 0.377702258 | 1.815494671 | 0.000287902 | 0.002713863 |
| PRUNE2 | PRUNE2_10234 | 0.377214065 | 4.679003628 | 3.79E-12 | 2.00E-10 |
| WSCD1 | WSCD1_16592 | 0.37664703 | 5.081061364 | 1.92E-23 | 4.10E-21 |
| TFPI2 | TFPI2_7847 | 0.376267874 | 5.841626134 | 2.89E-33 | 1.31E-30 |
| OSGIN1 | OSGIN1_16383 | 0.37568607 | 0.871542766 | 0.006966637 | 0.036455722 |
| AQP4 | AQP4_17804 | 0.37501555 | 1.481647769 | 0.000777777 | 0.006211806 |
| TMEM217 | TMEM217_6730 | 0.374903436 | 2.167933303 | 3.61E-05 | 0.000459738 |
| SLC15A3 | SLC15A3_12200 | 0.373528734 | 2.01909468 | 4.17E-05 | 0.000520635 |
| BNC2 | BNC2_9982 | 0.373217207 | 3.506632992 | 4.68E-06 | 7.58E-05 |
| BDNF | BDNF_11929 | 0.373060641 | 4.14358394 | 1.11E-13 | 7.47E-12 |
| UNC13A | UNC13A_19071 | 0.372176024 | 1.29214328 | 0.003184555 | 0.01970748 |
| MICB | MICB_6568 | 0.371491562 | 1.180388327 | 0.002571798 | 0.016621721 |
| F11R | F11R_1524 | 0.371268061 | 3.828672572 | 4.89E-10 | 1.79E-08 |

| | | | | | |
|---|---|---|---|---|---|
| ECM1 | ECM1_1223 | 0.370928465 | 4.321209364 | 1.58E-13 | 1.03E-11 |
| COL6A3 | COL6A3_3406 | 0.370895424 | 1.358075832 | 0.00203885 | 0.013794833 |
| OCEL1 | OCEL1_19052 | 0.370715212 | 1.222973678 | 0.00488852 | 0.027657121 |
| ABCB4 | ABCB4_7811 | 0.370221813 | 1.96585428 | 0.000142603 | 0.00149508 |
| PSMB9 | PSMB9_6642 | 0.369833116 | 1.851489278 | 0.000219175 | 0.002149815 |
| TRIM22 | TRIM22_11746 | 0.369374206 | 4.383217078 | 1.06E-12 | 6.03E-11 |
| PAMR1 | PAMR1_11968 | 0.368603082 | 1.178147696 | 0.003948857 | 0.0233689 |
| SQSTM1 | SQSTM1_6242 | 0.36768742 | 6.845423212 | 6.65E-43 | 6.61E-40 |
| LRRC55 | LRRC55_12115 | 0.367657434 | 3.31929255 | 1.60E-10 | 6.51E-09 |
| LUM | LUM_13647 | 0.367383717 | 2.169015288 | 0.000159181 | 0.001643114 |
| GPSM3 | GPSM3_6627 | 0.367257888 | 1.902155444 | 0.000254484 | 0.002446295 |
| RAB11FIP5 | RAB11FIP5_2538 | 0.367167294 | 3.37935691 | 5.79E-10 | 2.10E-08 |
| PCBP3 | PCBP3_20767 | 0.366845017 | 2.116552334 | 0.003772471 | 0.022503599 |
| CYP1B1-AS1 | CYP1B1-AS1_2358 | 0.366191397 | 2.123216361 | 0.00013873 | 0.001461664 |
| HBEGF | HBEGF_5926 | 0.364156778 | 6.055417057 | 1.26E-21 | 2.38E-19 |
| ABCA13 | ABCA13_7638 | 0.362523364 | 4.000344437 | 4.03E-14 | 2.86E-12 |
| ICAM1 | ICAM1_18856 | 0.361293824 | 4.905513608 | 1.19E-10 | 4.94E-09 |
| SYNM | SYNM_15569 | 0.360003132 | 6.11566823 | 1.47E-34 | 8.12E-32 |
| DHCR7 | DHCR7_12464 | 0.359776186 | 7.039141816 | 3.47E-38 | 2.47E-35 |
| PLXND1 | PLXND1_4188 | 0.359758417 | 7.582555366 | 3.20E-24 | 7.47E-22 |
| TAP1 | TAP1_6643 | 0.359727515 | 1.822566716 | 0.000465093 | 0.00409848 |
| RAB3B | RAB3B_719 | 0.35946677 | 3.403769967 | 3.77E-09 | 1.19E-07 |
| RAB11FIP1 | RAB11FIP1_9434 | 0.358333526 | 1.909247087 | 0.000195091 | 0.00195078 |
| TMEM170B | TMEM170B_6338 | 0.357640826 | 6.236837723 | 1.29E-21 | 2.41E-19 |
| SP140L | SP140L_3347 | 0.357027406 | 2.499083668 | 7.64E-05 | 0.000879102 |
| DUSP4 | DUSP4_9404 | 0.356970482 | 4.885574506 | 6.65E-12 | 3.34E-10 |
| GLRA3 | GLRA3_5302 | 0.354825409 | 1.350090617 | 0.008078183 | 0.041062491 |
| SPTBN5 | SPTBN5_15111 | 0.353839359 | 1.612313949 | 0.001123913 | 0.008464243 |
| ANKRD1 | ANKRD1_11249 | 0.35355744 | 8.972358659 | 4.55E-28 | 1.33E-25 |
| SLC25A43 | SLC25A43_8918 | 0.35312672 | 2.30048975 | 1.39E-05 | 0.000199965 |
| HIVEP3 | HIVEP3_601 | 0.352383931 | 4.299828513 | 4.14E-08 | 1.07E-06 |
| NFE2L3 | NFE2L3_7492 | 0.3512384 | 4.475578403 | 1.19E-10 | 4.94E-09 |
| C5orf45 | C5orf45_6243 | 0.349753222 | 5.930469944 | 7.86E-33 | 3.30E-30 |
| AP1M2 | AP1M2_18871 | 0.349410728 | 2.986734202 | 3.14E-06 | 5.31E-05 |
| PMAIP1 | PMAIP1_17923 | 0.34918892 | 3.20026058 | 5.43E-08 | 1.37E-06 |
| PLEKHA4 | PLEKHA4_19631 | 0.348756077 | 4.469571348 | 9.55E-16 | 8.48E-14 |
| PARD6A | PARD6A_16247 | 0.348352932 | 2.200670141 | 4.75E-05 | 0.000583646 |
| KCNJ6 | KCNJ6_20667 | 0.348100529 | 4.433240062 | 2.11E-07 | 4.78E-06 |
| STAP2 | STAP2_18702 | 0.34769219 | 2.60627889 | 5.88E-06 | 9.36E-05 |
| HS1BP3 | HS1BP3_2230 | 0.3473213 | 4.060515747 | 5.83E-11 | 2.53E-09 |
| S100A11 | S100A11_1272 | 0.346472196 | 2.67711089 | 0.008672566 | 0.043359922 |

| | | | | | |
|---|---|---|---|---|---|
| VSIG10L | VSIG10L_19740 | 0.34515068 | 0.98021 | 0.009243309 | 0.045501968 |
| CRYBA1 | CRYBA1_16882 | 0.343492183 | 1.672081056 | 0.001839099 | 0.012702555 |
| HSD17B7 | HSD17B7_1565 | 0.343228083 | 4.867311618 | 6.24E-20 | 9.12E-18 |
| CX3CL1 | CX3CL1_16168 | 0.34314575 | 3.108554668 | 8.99E-06 | 0.000137696 |
| HID1 | HID1_17521 | 0.342809494 | 1.740012944 | 0.000506149 | 0.004369932 |
| RAB11FIP4 | RAB11FIP4_16908 | 0.342300665 | 2.308897351 | 2.13E-05 | 0.000291868 |
| HAPLN4 | HAPLN4_19121 | 0.342286286 | 1.18496156 | 0.004737579 | 0.026966786 |
| ZCWPW2 | ZCWPW2_3592 | 0.342150063 | 0.972303919 | 0.009801296 | 0.047677058 |
| GDF6 | GDF6_9673 | 0.342111238 | 4.314929633 | 1.53E-12 | 8.45E-11 |
| CPEB2 | CPEB2_4690 | 0.341597352 | 4.535268684 | 7.12E-12 | 3.54E-10 |
| BRI3 | BRI3_7871 | 0.341545713 | 5.854568496 | 6.06E-26 | 1.56E-23 |
| SULF1 | SULF1_9569 | 0.341419972 | 3.006267858 | 5.53E-05 | 0.000666146 |
| HSD17B7P2 | HSD17B7P2_10948 | 0.340142331 | 4.098898644 | 2.60E-12 | 1.39E-10 |
| HTATIP2 | HTATIP2_11908 | 0.339143289 | 1.023984473 | 0.008269445 | 0.041799127 |
| DBNDD2 | DBNDD2_18359 | 0.338819329 | 3.82232223 | 2.67E-10 | 1.03E-08 |
| SLC2A14 | SLC2A14_13033 | 0.338411615 | 4.520442583 | 1.54E-15 | 1.33E-13 |
| HSBP1L1 | HSBP1L1_17985 | 0.337460677 | 1.900870447 | 0.000347083 | 0.00318263 |
| PROSER2 | PROSER2_10821 | 0.336310955 | 2.024365481 | 0.000759398 | 0.00609158 |
| SYT14 | SYT14_1878 | 0.336073124 | 2.52196924 | 6.36E-05 | 0.000749508 |
| LZTS1 | LZTS1_9329 | 0.336005565 | 6.440491842 | 1.34E-15 | 1.17E-13 |
| ARHGAP18 | ARHGAP18_7141 | 0.334312584 | 3.078659512 | 6.63E-05 | 0.000777753 |
| C10orf35 | C10orf35_11102 | 0.333403283 | 2.110297037 | 0.000633653 | 0.005275372 |
| NKD2 | NKD2_5385 | 0.333318776 | 2.650988425 | 0.000212733 | 0.002097659 |
| TSPO | TSPO_20453 | 0.333131886 | 2.017771627 | 0.001265204 | 0.009362884 |
| GTF2A1L | GTF2A1L_2419 | 0.330935685 | 2.80857673 | 1.72E-06 | 3.13E-05 |
| MVD | MVD_16429 | 0.330843752 | 6.66098702 | 2.05E-31 | 7.65E-29 |
| Mar-04 | MARCH4_3246 | 0.330656309 | 2.766519384 | 0.002190716 | 0.014639792 |
| IGFBP3 | IGFBP3_7628 | 0.330518608 | 7.741149297 | 2.70E-13 | 1.67E-11 |
| LHCGR | LHCGR_2420 | 0.330335396 | 2.834929991 | 1.38E-06 | 2.57E-05 |
| TRIB3 | TRIB3_17999 | 0.329649397 | 3.171503778 | 1.04E-07 | 2.48E-06 |
| HMGCS1 | HMGCS1_5515 | 0.328894215 | 10.05609276 | 5.92E-45 | 6.79E-42 |
| COL12A1 | COL12A1_6938 | 0.328162645 | 5.786324843 | 1.98E-15 | 1.65E-13 |
| HPCAL4 | HPCAL4_573 | 0.327215214 | 2.257124487 | 0.000100146 | 0.001108365 |
| SCN9A | SCN9A_2996 | 0.327118817 | 4.228126587 | 5.45E-11 | 2.38E-09 |
| SLC7A5 | SLC7A5_16419 | 0.326769327 | 8.188212404 | 1.53E-19 | 2.15E-17 |
| ST3GAL5 | ST3GAL5_2617 | 0.326693313 | 3.495208855 | 1.56E-07 | 3.61E-06 |
| FAM131B | FAM131B_8214 | 0.325694911 | 4.965691066 | 7.27E-13 | 4.22E-11 |
| SLC9A3R2 | SLC9A3R2_15697 | 0.323274745 | 3.391065063 | 8.68E-06 | 0.000133568 |
| MYEOV | MYEOV_12453 | 0.322968004 | 2.762927976 | 0.000209339 | 0.002068251 |
| CDKN2B | CDKN2B_10017 | 0.32227338 | 4.337075098 | 1.47E-09 | 5.04E-08 |
| ARHGEF19 | ARHGEF19_239 | 0.322265773 | 2.677571242 | 6.47E-06 | 0.000102672 |

| | | | | | |
|---|---|---|---|---|---|
| SOX8 | SOX8_15650 | 0.322132411 | 1.610673601 | 0.002299682 | 0.015234883 |
| CPPED1 | CPPED1_15834 | 0.32121854 | 2.75014381 | 9.67E-06 | 0.00014588 |
| CXCL6 | CXCL6_4906 | 0.321035392 | 1.733765246 | 0.005807119 | 0.031550039 |
| RASGRF1 | RASGRF1_15441 | 0.321034896 | 1.194219743 | 0.009776092 | 0.047585489 |
| LPCAT2 | LPCAT2_16134 | 0.320877584 | 3.682328446 | 2.42E-08 | 6.58E-07 |
| COL25A1 | COL25A1_5078 | 0.320675806 | 3.028553916 | 2.46E-05 | 0.000329677 |

| Genes bound by LSD1 and downregulated upon LSD1 inhibition | | | | | |
|---|---|---|---|---|---|
| **Gene name** | **gene_ID** | **logFC** | **logCPM** | **PValue** | **FDR** |
| CNTN2 | CNTN2_1821 | -1.514186467 | 0.200874416 | 2.27E-15 | 1.87E-13 |
| STMN2 | STMN2_9605 | -1.085922784 | 0.214441869 | 6.44E-10 | 2.31E-08 |
| HES5 | HES5_75 | -0.975515131 | 0.05938655 | 2.12E-07 | 4.80E-06 |
| OPRK1 | OPRK1_9505 | -0.840515168 | 2.685576818 | 2.04E-22 | 4.12E-20 |
| TSHZ1 | TSHZ1_17971 | -0.812322582 | 0.571592939 | 2.22E-05 | 0.000301183 |
| NCAM1-AS1 | NCAM1-AS1_12720 | -0.663287142 | 1.034351595 | 2.42E-07 | 5.38E-06 |
| PRMT8 | PRMT8_12956 | -0.60215432 | 4.330360526 | 5.30E-17 | 5.69E-15 |
| VCAM1 | VCAM1_972 | -0.593033826 | 5.113019815 | 2.62E-47 | 3.26E-44 |
| RHBDL3 | RHBDL3_16914 | -0.588409921 | 3.299847119 | 3.52E-12 | 1.86E-10 |
| IMPG2 | IMPG2_4004 | -0.575777154 | 0.819995056 | 6.74E-05 | 0.000789234 |
| FRAT1 | FRAT1_11306 | -0.548887604 | 0.463263802 | 0.00069779 | 0.005701163 |
| ZNF503-AS2 | ZNF503-AS2_11170 | -0.54381439 | 1.093852881 | 1.93E-05 | 0.000267124 |
| PCDH8 | PCDH8_14197 | -0.529159353 | 5.413304059 | 1.97E-20 | 3.09E-18 |
| CDS1 | CDS1_4982 | -0.51800646 | 1.34193878 | 3.49E-05 | 0.000446921 |
| ZNF503 | ZNF503_11169 | -0.515992483 | 2.297581586 | 2.00E-08 | 5.57E-07 |
| SOX3 | SOX3_9043 | -0.511197112 | 3.147009325 | 1.00E-14 | 7.73E-13 |
| LRRTM1 | LRRTM1_2591 | -0.509961051 | -0.017086889 | 0.006184648 | 0.033130617 |
| DLL1 | DLL1_7341 | -0.506121025 | 3.345515768 | 6.76E-14 | 4.69E-12 |
| CNR1 | CNR1_6987 | -0.505847254 | 7.271062596 | 2.92E-31 | 1.06E-28 |
| MMD2 | MMD2_7387 | -0.504542611 | 0.200882928 | 0.00446713 | 0.025857425 |
| DCX | DCX_8891 | -0.500998851 | 5.179559015 | 4.15E-21 | 7.20E-19 |
| C5orf49 | C5orf49_5409 | -0.498270792 | 1.834588764 | 2.30E-07 | 5.16E-06 |
| MDGA1 | MDGA1_6735 | -0.481303783 | 3.471852941 | 6.10E-11 | 2.64E-09 |
| EPHA3 | EPHA3_3963 | -0.477791332 | 3.090292512 | 5.98E-15 | 4.67E-13 |
| SNCAIP | SNCAIP_5799 | -0.47589291 | 3.030841075 | 3.36E-13 | 2.05E-11 |
| GLI1 | GLI1_13508 | -0.475247284 | 1.374236584 | 8.53E-05 | 0.000962869 |
| C17orf97 | C17orf97_16472 | -0.463926529 | 0.822470463 | 0.010037895 | 0.048507729 |
| TMEM35 | TMEM35_8798 | -0.463539665 | 2.543509648 | 6.06E-08 | 1.52E-06 |
| FGFR2 | FGFR2_11490 | -0.461193692 | 4.931681192 | 4.64E-20 | 6.86E-18 |
| FZD7 | FZD7_3177 | -0.456227472 | 5.440161069 | 8.00E-24 | 1.76E-21 |
| GADD45G | GADD45G_10288 | -0.450167678 | 3.045859019 | 2.00E-08 | 5.57E-07 |
| PCDH19 | PCDH19_8787 | -0.439519739 | 6.556765451 | 1.33E-33 | 6.59E-31 |
| EBF4 | EBF4_18035 | -0.43949534 | 2.61998452 | 2.75E-06 | 4.75E-05 |
| BMF | BMF_15068 | -0.438078583 | 4.014134413 | 1.73E-15 | 1.47E-13 |
| NDNF | NDNF_5126 | -0.43741702 | 3.872833481 | 6.40E-18 | 7.66E-16 |
| FGF10 | FGF10_5522 | -0.436406552 | 2.456686363 | 9.79E-08 | 2.34E-06 |
| CYP26A1 | CYP26A1_11266 | -0.43379955 | 5.31881213 | 6.32E-19 | 8.34E-17 |
| RUNX1T1 | RUNX1T1_9651 | -0.432542008 | 2.625536716 | 1.17E-06 | 2.21E-05 |
| DACT3 | DACT3_19567 | -0.429788415 | 2.788285342 | 2.73E-10 | 1.05E-08 |
| ZNF385B | ZNF385B_3084 | -0.426620168 | 0.525567862 | 0.004632787 | 0.02660375 |
| PAIP2B | PAIP2B_2530 | -0.424551885 | 1.432772632 | 0.000185624 | 0.001869897 |

| | | | | | |
|---|---|---|---|---|---|
| TCP11L2 | TCP11L2_13737 | -0.42247074 | 0.754013463 | 0.003991743 | 0.023603968 |
| NME3 | NME3_15675 | -0.42215689 | 0.710740573 | 0.00537889 | 0.029835964 |
| ARRDC4 | ARRDC4_15565 | -0.414447256 | 0.996177527 | 0.002236777 | 0.014884244 |
| CLNK | CLNK_4684 | -0.413991536 | 0.769508275 | 0.007342429 | 0.038061742 |
| CA2 | CA2_9629 | -0.412579897 | 1.699316839 | 0.0006601 | 0.005445682 |
| NPY2R | NPY2R_5239 | -0.412186668 | 2.915387727 | 5.29E-09 | 1.64E-07 |
| DNAJC12 | DNAJC12_11076 | -0.408343456 | 0.793296793 | 0.006514822 | 0.034490641 |
| MAPK8IP2 | MAPK8IP2_20523 | -0.407277772 | 4.993696393 | 8.89E-24 | 1.92E-21 |
| KCNH8 | KCNH8_3567 | -0.405737444 | 4.048538093 | 5.55E-10 | 2.02E-08 |
| HS3ST1 | HS3ST1_4685 | -0.40167311 | 1.787881027 | 0.000493643 | 0.004289263 |
| MIR940 | MIR940_15712 | -0.394435884 | 0.91340504 | 0.008154453 | 0.041379689 |
| CDKN2C | CDKN2C_711 | -0.394393859 | 1.472398429 | 0.000542206 | 0.004638283 |
| PIF1 | PIF1_15284 | -0.394158018 | 3.826660351 | 2.54E-09 | 8.35E-08 |
| PRDM8 | PRDM8_4958 | -0.393701926 | 1.743552597 | 0.001862719 | 0.012824138 |
| MAP2K6 | MAP2K6_17483 | -0.390672692 | 3.489318063 | 4.99E-07 | 1.02E-05 |
| PTCH1 | PTCH1_10324 | -0.386680979 | 6.425867994 | 1.23E-28 | 3.98E-26 |
| RPS6KA6 | RPS6KA6_8767 | -0.386276623 | 4.296038612 | 8.43E-17 | 8.67E-15 |
| KCNH2 | KCNH2_8277 | -0.375553468 | 2.080309377 | 4.04E-05 | 0.00050801 |
| NCAM1 | NCAM1_12719 | -0.369136185 | 5.089421774 | 2.70E-16 | 2.51E-14 |
| PCDH18 | PCDH18_5163 | -0.366302041 | 6.094502215 | 1.61E-28 | 4.99E-26 |
| ANKRD30BL | ANKRD30BL_2898 | -0.359591233 | 5.548677871 | 0.001746429 | 0.012163855 |
| RGMA | RGMA_15561 | -0.358304603 | 7.0012619 | 4.06E-21 | 7.12E-19 |
| GAS1 | GAS1_10269 | -0.355777606 | 2.958657015 | 1.49E-06 | 2.75E-05 |
| KLF15 | KLF15_4144 | -0.355486668 | 1.143569723 | 0.003930871 | 0.023299431 |
| TMEM196 | TMEM196_7460 | -0.354736666 | 1.340080368 | 0.002035355 | 0.013790002 |
| TOX2 | TOX2_18328 | -0.353987531 | 4.622500864 | 3.08E-13 | 1.90E-11 |
| TRHDE | TRHDE_13595 | -0.351951183 | 1.848762057 | 0.00040014 | 0.003591868 |
| DTX4 | DTX4_12160 | -0.349686887 | 7.236390616 | 1.35E-20 | 2.18E-18 |
| KCNC4 | KCNC4_1036 | -0.349255151 | 0.899707163 | 0.009527886 | 0.04648681 |
| EFNB3 | EFNB3_16654 | -0.346145592 | 5.619432416 | 1.53E-24 | 3.61E-22 |
| EFNA4 | EFNA4_1374 | -0.34445703 | 3.434409959 | 9.24E-09 | 2.75E-07 |
| PABPC5 | PABPC5_8779 | -0.343050383 | 2.549047347 | 9.72E-06 | 0.00014649 |
| NR2F1-AS1 | NR2F1-AS1_5712 | -0.340403591 | 7.213565371 | 1.10E-08 | 3.24E-07 |
| CDC42EP4 | CDC42EP4_17494 | -0.339518955 | 4.334448218 | 8.83E-11 | 3.71E-09 |
| KIRREL2 | KIRREL2_19261 | -0.336424391 | 1.372518714 | 0.009829241 | 0.047766269 |
| RTN1 | RTN1_14627 | -0.333906279 | 1.431061165 | 0.002693722 | 0.017262729 |
| PAX6 | PAX6_11941 | -0.332023137 | 3.934106613 | 6.19E-09 | 1.90E-07 |
| ICA1L | ICA1L_3185 | -0.330416404 | 3.188868116 | 4.70E-07 | 9.66E-06 |
| NR2F1 | NR2F1_5713 | -0.32858759 | 8.019358802 | 4.91E-11 | 2.16E-09 |
| CEP19 | CEP19_4548 | -0.326901248 | 1.815415904 | 0.000923645 | 0.00717701 |
| ST6GAL2 | ST6GAL2_2762 | -0.3265123 | 1.164479216 | 0.00941475 | 0.046112496 |
| CENPE | CENPE_5056 | -0.325639971 | 6.386674798 | 4.28E-21 | 7.33E-19 |
| SEMA5A | SEMA5A_5413 | -0.325502435 | 6.125072754 | 1.45E-28 | 4.61E-26 |

| | | | | | |
|---|---|---|---|---|---|
| LAMA4 | LAMA4_7077 | -0.325077563 | 4.492032977 | 1.38E-09 | 4.75E-08 |
| ST8SIA4 | ST8SIA4_5740 | -0.324880534 | 3.851151669 | 1.12E-06 | 2.12E-05 |
| HS3ST3B1 | HS3ST3B1_16722 | -0.324572056 | 5.560283061 | 2.21E-16 | 2.08E-14 |
| ABCA7 | ABCA7_18595 | -0.322100957 | 2.10599043 | 0.00143615 | 0.010416773 |

**Biologicial pathways associated with the genes upregualted upon LSD1 inhibition**

| signal_transduction | cell_adhesion | positive_regulation_of_transcription_from_RNA_polymerase_II_promoter |
|---|---|---|
| 41 | 34 | 32 |
| LGALS3BP | LGALS3BP | CSRNP1 |
| CXCL6 | COL18A1 | CEBPB |
| ITK | PTPRU | CEBPD |
| BEX2 | ACHE | EPAS1 |
| ECM1 | COL12A1 | PTPRN |
| RRAD | ITGA2B | SERPINE1 |
| EPAS1 | TNC | NLRC5 |
| LRRK1 | LAMC2 | CX3CL1 |
| RASGRF1 | ICAM5 | FSTL3 |
| STC1 | HAPLN3 | CREB3L1 |
| TGFA | F11R | SOX8 |
| TTBK1 | HAPLN4 | HTATIP2 |
| FAM83G | CLDN1 | IL11 |
| TMEM102 | CX3CL1 | CDKN2B |
| GLRA3 | ICAM1 | LUM |
| PLAU | SPP1 | WNT3A |
| OLFML3 | EMILIN2 | LIF |
| INPP5D | CYP1B1 | IGF2 |
| SPP1 | EMILIN1 | NR1H4 |
| SOX8 | SCARF1 | NFATC2 |
| CD34 | CD34 | INHBA |
| S100A11 | IL32 | ETV4 |
| IL32 | ITGA3 | POU4F1 |
| IL11 | CLDN6 | GREM1 |
| CHRNB1 | COL1A1 | IL1A |
| ERAP2 | BCAN | GAL |
| GDF15 | CLDN4 | GDNF |
| GABRA5 | PARVG | F2RL1 |
| TIE1 | COL6A2 | SQSTM1 |
| PLAUR | ADAM12 | LPIN3 |
| OSGIN1 | COL6A3 | ENG |
| HPCAL4 | CD9 | TP73 |
| PGF | TGFBI | |
| GREM1 | ENG | |
| SIGIRR | | |
| VGF | | |
| GDNF | | |
| TLR6 | | |
| GRAPL | | |
| RAPGEF3 | | |

| | | |
|---|---|---|
| HBEGF | | |

| extracellular_matrix_organization | positive_regulation_of_gene_expression | cell_differentiation |
|---|---|---|
| 31 | 26 | 25 |
| COL17A1 | MYOM1 | PTPRU |
| COL18A1 | TNC | COL13A1 |
| COL13A1 | TTBK1 | ELAVL3 |
| ELN | CDH5 | FSTL3 |
| ITGA2B | LAMP3 | HTATIP2 |
| SERPINE1 | PLCG2 | JAG2 |
| TNC | SOX8 | SLC2A14 |
| LAMC2 | EMILIN1 | NKX6-2 |
| FURIN | CD34 | UNC13A |
| ICAM5 | IL32 | FST |
| F11R | CD74 | BMP8A |
| FBLN2 | ITGA3 | NR1H4 |
| ICAM1 | NOS3 | OSGIN1 |
| ADAMTS16 | WNT3A | BMP8B |
| ADAMTS14 | LIF | MICAL2 |
| KDR | NFATC2 | INHBA |
| SPP1 | SERPINB9 | FOXL1 |
| ITGA3 | INHBA | ETV4 |
| LUM | POU4F1 | DLK1 |
| WNT3A | IL1A | PGF |
| COL1A1 | ADM2 | SPOCD1 |
| BCAN | ID1 | ID1 |
| SPINT1 | TMEM119 | SLCO4C1 |
| CRISPLD2 | ID3 | ID3 |
| COL6A2 | TLR6 | SQSTM1 |
| ADAM12 | ENG | |
| COL6A3 | | |
| VWA1 | | |
| COL9A3 | | |
| TGFBI | | |
| COL9A2 | | |
| | | |
| | | |
| | | |
| | | |
| | | |
| | | |
| | | |
| | | |
| | | |

| multicellular_organism_development | response_to_drug | apoptotic_process |
|---|---|---|
| 24 | 23 | 22 |
| SLC2A14 | COL18A1 | CSRNP1 |
| CATSPER3 | CPT1A | BEX2 |
| TIE1 | NNMT | STEAP3 |
| FST | HMGCS1 | IGFBP3 |
| PLEKHB1 | ITGA3 | PRUNE2 |
| SPRY4 | GAD1 | PCSK9 |
| LIF | ABCA3 | SERPINB9 |
| IGF2 | NFATC2 | CFLAR |
| OSGIN1 | TGFA | PTPRH |
| MICAL2 | CYBA | SULF1 |
| SHISA2 | INHBA | GDF6 |
| FOXL1 | ASS1 | GREM1 |
| HIC1 | ICAM1 | IL1A |
| FSTL3 | COL1A1 | TMEM102 |
| DLL3 | HSD11B2 | LGALS1 |
| ANO1 | FABP3 | INPP5D |
| CDH5 | GAL | PMAIP1 |
| HOOK1 | LGALS1 | PHLDA2 |
| OLFML3 | TSPO | SQSTM1 |
| CREB3L1 | HMOX1 | BIRC3 |
| SLCO4C1 | ENG | HTATIP2 |
| ARHGDIB | S100A10 | TP73 |
| KDR | TP73 | |
| ID3 | | |
| | | |
| | | |
| | | |
| | | |
| | | |
| | | |
| | | |
| | | |
| | | |
| | | |
| | | |
| | | |
| | | |
| | | |
| | | |
| | | |

| positive_regulation_of_angiogenesis | angiogenesis | positive_regulation_of_apoptotic_process |
| --- | --- | --- |
| 20 | 20 | 19 |
| ECM1 | COL18A1 | NGFR |
| NOS3 | ECM1 | IGFBP3 |
| TIE1 | PLXND1 | ARHGEF19 |
| SERPINE1 | NOS3 | OSGIN1 |
| CX3CL1 | EPAS1 | POU4F1 |
| PGF | TIE1 | GAL |
| GREM1 | ITGA2B | LGALS1 |
| IL1A | SERPINE1 | INPP5D |
| CDH5 | TGFA | EEF1A2 |
| TMIGD2 | EFNA1 | ANKRD1 |
| TERT | ADM2 | TSPO |
| ADM2 | ID1 | EMILIN2 |
| ADAM12 | KDR | ID3 |
| KDR | CYP1B1 | CYP1B1 |
| EMILIN2 | HMOX1 | PMAIP1 |
| CYP1B1 | TGFBI | HMOX1 |
| HMOX1 | SCG2 | PHLDA2 |
| CD34 | RAPGEF3 | SQSTM1 |
| RAPGEF3 | ENG | TP73 |
| ENG | HTATIP2 | |
| | | |
| | | |
| | | |
| | | |
| | | |
| | | |
| | | |
| | | |
| | | |
| | | |
| | | |
| | | |
| | | |
| | | |
| | | |
| | | |
| | | |
| | | |
| | | |
| | | |

| **response_to_xenobiotic_stimulus** | **negative_regulation_of_apoptotic_process** | **intracellular_signal_transduction** |
|---|---|---|
| 18 | 18 | 17 |
| CPT1A | NGFR | ITK |
| NNMT | CD74 | SHC2 |
| ITGA3 | PLAUR | UNC13A |
| ABCA3 | NR1H4 | CDC42BPG |
| NFATC2 | TGFA | NRK |
| TGFA | SERPINB9 | PLA2G4C |
| CYBA | CFLAR | NLRC5 |
| INHBA | POU4F1 | TGFA |
| ENO2 | CX3CL1 | DEPDC7 |
| PGF | GREM1 | NRG3 |
| ASS1 | KRT18 | INPP5D |
| COL1A1 | GDNF | PLCG2 |
| HSD11B2 | ID1 | HMOX1 |
| GAL | KDR | SQSTM1 |
| TSPO | SOX8 | SCG2 |
| HMOX1 | SQSTM1 | PLCB2 |
| ENG | BIRC3 | RGS9 |
| TP73 | HTATIP2 | |
| | | |
| | | |
| | | |
| | | |
| | | |
| | | |
| | | |
| | | |
| | | |
| | | |
| | | |
| | | |
| | | |
| | | |
| | | |
| | | |
| | | |
| | | |
| | | |
| | | |
| | | |
| | | |

| **inflammatory_response** | **immune_response** | **collagen_fibril_organization** |
|---|---|---|
| 16 | 16 | 16 |
| CXCL6 | IL32 | COL17A1 |
| ECM1 | CXCL6 | COL18A1 |
| CEBPB | CD74 | COL13A1 |
| PLA2G4C | CEBPB | COL25A1 |
| NR1H4 | LIF | LUM |
| CYBA | SERPINB9 | COL12A1 |
| F11R | CRIP1 | LOXL4 |
| CX3CL1 | CX3CL1 | LAMC2 |
| IL1A | IL1A | GREM1 |
| GAL | TMIGD2 | COL1A1 |
| SCN9A | CCR7 | ADAMTS14 |
| SPP1 | MBP | COL6A2 |
| F2RL1 | TLR6 | CYP1B1 |
| CCR7 | GPSM3 | COL6A3 |
| TLR6 | TRIM22 | COL9A3 |
| SCG2 | MICB | COL9A2 |
| | | |
| | | |
| | | |
| | | |
| | | |
| | | |
| | | |
| | | |
| | | |
| | | |
| | | |
| | | |
| | | |
| | | |
| | | |
| | | |
| | | |
| | | |
| | | |
| | | |
| | | |
| | | |
| | | |
| | | |

| cellular_protein_metabolic_process | transmembrane_transport | post-translational_protein_modification |
|---|---|---|
| 16 | 15 | 15 |
| ABCA3 | OCA2 | EPAS1 |
| IGFBP3 | ABCB4 | IGFBP3 |
| TNC | ABCA4_TAP2_TAP1 | TNC |
| IGF2 | SLC4A11 | PCSK9 |
| PCSK9 | AQP4 | FSTL3 |
| FSTL3 | ANO4 | PSMB9 |
| LGALS1 | ABCA13 | LGALS1 |
| VGF | PSMB9 | VGF |
| STC2 | ANO1 | STC2 |
| PAPPA | SLCO4C1 | SPP1 |
| SPP1 | SLC16A6 | FAM20C |
| FAM20C | SLC25A43 | VWA1 |
| VWA1 | SLC19A1 | NOTUM |
| NOTUM | APOE | TRIM22 |
| APOE | SCG2 | BIRC3 |
| SCG2 | | |

| positive_regulation_of_I-kappaB_kinase_NF-kappaB_signaling | negative_regulation_of_cell_proliferation | cell-cell_adhesion |
|---|---|---|
| 15 | 15 | 14 |
| CD74 | COL18A1 | COL13A1 |
| ECM1 | PTPRU | ITGA3 |
| CFLAR | CDKN2B | ITGA2B |
| S100B | NOS3 | AQP4 |
| CX3CL1 | IGFBP3 | ICAM5 |
| IL1A | LIF | F11R |
| LGALS1 | INHBA | CX3CL1 |
| F2RL1 | IL1A | ICAM1 |
| HMOX1 | CDH5 | CDH5 |
| S100A4 | FABP3 | KRT18 |
| CCR7 | CD9 | LGALS1 |
| TRIM38 | CYP1B1 | PKP2 |
| TLR6 | INSM1 | CD34 |
| S100A11 | S100A11 | |
| TP73 | | |
| | | |
| | | |
| | | |
| | | |
| | | |
| | | |
| | | |
| | | |
| | | |
| | | |
| | | |
| | | |
| | | |
| | | |
| | | |
| | | |
| | | |
| | | |
| | | |
| | | |
| | | |
| | | |
| | | |
| | | |
| | | |

| regulation_of_ion_transmembrane_transport | negative_regulation_of_gene_expression | MAPK_cascade |
|---|---|---|
| 13 | 13 | 13 |
| KCNK5 | PLA2G3 | SPTBN5 |
| KCNJ4 | POU4F1 | SHC2 |
| CLIC6 | TTBK1 | NRK |
| KCNG3 | SLC7A5 | RASGRF1 |
| CATSPER3 | TERT | TGFA |
| KCNJ6 | STC2 | SPTB |
| CLCNKB | CREB3L1 | PSMB9 |
| CACNA1B | KDR | SBK2 |
| CACNG6 | ID3 | GDNF |
| KCNU1 | EMILIN1 | MBP |
| SCN9A | APOE | SCG2 |
| KCNQ4 | CD34 | PTPN3 |
| KCNJ2 | ENG | HBEGF |

| skeletal_system_development | regulation_of_apoptotic_process | positive_regulation_of_cell_migration |
|---|---|---|
| 12 | 12 | 12 |
| JAG2 | BEX2 | COL1A1 |
| COL1A1 | TMEM102 | CDH5 |
| BCAN | STEAP3 | CLDN4 |
| COL13A1 | LGALS1 | PLAU |
| FST | IGFBP3 | FAM110C |
| IGF2 | OSGIN1 | SNAI1 |
| BMP8B | PMAIP1 | KDR |
| ALPL | HS1BP3 | F2RL1 |
| COL9A2 | CFLAR | LAMC2 |
| HAPLN3 | APOE | INSM1 |
| HAPLN4 | TP73 | CLDN1 |
| DLL3 | BIRC3 | HBEGF |

| positive_regulation_of_MAPK_cascade | positive_regulation_of_GTPase_activity | cytokine-mediated_signaling_pathway |
|---|---|---|
| 12 | 12 | 12 |
| EFNA1 | RAP1GAP2 | IL32 |
| IL11 | ADAP2 | IL1A |
| CD74 | PLEKHG6 | IL11 |
| SHC2 | RASGRF1 | CEBPD |
| GDF15 | ARHGDIB | MAOA |
| IGFBP3 | F2RL1 | INPP5D |
| PLCG2 | F11R | PTPRN |
| LIF | CX3CL1 | LIF |
| IGF2 | RAPGEF3 | HMOX1 |
| KDR | ICAM1 | PLP2 |
| CX3CL1 | S100A10 | CX3CL1 |
| TP73 | RAPGEF4 | ICAM1 |
| | | |
| | | |
| | | |
| | | |
| | | |
| | | |
| | | |
| | | |
| | | |
| | | |
| | | |
| | | |
| | | |
| | | |
| | | |
| | | |
| | | |
| | | |
| | | |
| | | |
| | | |
| | | |
| | | |
| | | |
| | | |
| | | |
| | | |
| | | |

| chemical_synaptic_transmission | visual_perception | positive_regulation_of_protein_phosphorylation |
|---|---|---|
| 12 | 11 | 11 |
| CHRNB1 | COL1A1 | EFNA1 |
| GPR176 | COL18A1 | CD74 |
| GLRA3 | MFRP | CD6 |
| GABRA5 | EPAS1 | WNT3A |
| KCNMB1 | LUM | TMEM119 |
| GAD1 | PRCD | PLAUR |
| HTR1D | ABCA4 | IGF2 |
| CACNA1B | CRYBA1 | KDR |
| SNCG | TGFBI | SQSTM1 |
| MBP | RGS9 | PGF |
| NPTX1 | NXNL2 | ENG |
| HAP1 | | |
| | | |
| | | |
| | | |
| | | |
| | | |
| | | |
| | | |
| | | |
| | | |
| | | |
| | | |
| | | |
| | | |
| | | |
| | | |
| | | |
| | | |
| | | |
| | | |
| | | |
| | | |
| | | |
| | | |
| | | |
| | | |
| | | |
| | | |

| positive_regulation_of_protein_kinase_B_signaling | osteoblast_differentiation | negative_regulation_of_cell_migration |
|---|---|---|
| 11 | 11 | 11 |
| GDF15 | COL1A1 | NGFR |
| FAM110C | WNT3A | PTPRU |
| IGF2 | CREB3L1 | CD74 |
| TGFA | IGFBP3 | TIE1 |
| TRIB3 | TMEM119 | SERPINE1 |
| CCR7 | SNAI1 | EMILIN2 |
| HPSE | SPP1 | CYP1B1 |
| CX3CL1 | IGF2 | STC1 |
| ENG | TNC | SULF1 |
| PIK3R5 | ALPL | CX3CL1 |
| HBEGF | SOX8 | ENG |

| central_nervous_system_development | cellular_response_to_hypoxia | heart_development |
|---|---|---|
| 11 | 11 | 10 |
| BCAN | TERT | IL1A |
| NGFR | EPAS1 | ADAP2 |
| SNTG2 | KCNMB1 | ITGA3_DNAH5 |
| ID3 | STC2 | PKP2 |
| MBP | ANKRD1 | ID3 |
| NPTX1 | PMAIP1 | MICAL2 |
| HAPLN3 | HMOX1 | FOXL1 |
| S100B | STC1 | POU4F1 |
| HPCAL4 | CFLAR | CRIP1 |
| HAPLN4 | CPEB2 | |
| NPAS1 | ICAM1 | |

| **response_to_hypoxia** | **positive_regulation_of_peptidyl-tyrosine_phosphorylation** | **positive_regulation_of_ERK1_and_ERK2_cascade** |
|---|---|---|
| 9 | 9 | 9 |
| IL1A | EFNA1 | IL1A |
| HSD11B2 | IL11 | CD74 |
| PLAU | CD74 | KDR |
| EPAS1 | LRRK1 | F2RL1 |
| HMOX1 | PLCG2 | CCR7 |
| CYBA | LIF | CFLAR |
| CYGB | IGF2 | APOE |
| PGF | TGFA | CX3CL1 |
| ENG | ICAM1 | ICAM1 |

| platelet_degranulation | negative_regulation_of_neuron_apoptotic_process | negative_regulation_of_endopeptidase_activity | leukocyte_migration |
|---|---|---|---|
| 9 | 9 | 9 | 9 |
| LGALS3BP | CEBPB | SPINT1 | COL1A1 |
| TOR4A | TERT | TFPI2 | SLC7A5 |
| ECM1 | GDNF | SERPINE1 | CD74 |
| ITGA2B | BDNF | COL6A3 | ITGA3 |
| STXBP2 | GABRA5 | FURIN | INPP5D |
| SERPINE1 | PLA2G3 | SERPINB9 | F2RL1 |
| IGF2 | HMOX1 | SPINT2 | F11R |
| CD9 | APOE | RENBP | CD34 |
| TAGLN2 | POU4F1 | SERPINB8 | ICAM1 |

| ion_transmembrane_transport | chloride_transport | cellular_response_to_tumor_necrosis_factor | cellular_response_to_lipopolysaccharide |
|---|---|---|---|
| 9 | 9 | 9 | 9 |
| CLIC6 | CLIC6 | COL1A1 | IL1A |
| ANO1 | ANO1 | OCSTAMP | CXCL6 |
| CHRNB1 | CLDN4 | ANKRD1 | CEBPB |
| GLRA3 | GLRA3 | CYP1B1 | SERPINE1 |
| GABRA5 | GABRA5 | CYBA | NR1H4 |
| GRIK5 | CLCNKB | CLDN1 | ANKRD1 |
| CLCNKB | TSPO | CX3CL1 | TSPO |
| PLP2 | ANO4 | ASS1 | ASS1 |
| ANO4 | BEST3 | ICAM1 | ICAM1 |

| cell-cell_signaling | transforming_growth_factor_beta_receptor_signaling_pathway | potassium_ion_transmembrane_transport | positive_regulation_of_neuron_projection_development |
|---|---|---|---|
| 9 | 8 | 8 | 8 |
| EFNA1 | CDH5 | KCNJ4 | BDNF |
| GREM1 | PARD6A | KCNK5 | ITGA3 |
| CXCL6 | GDF15 | KCNG3 | ANKRD1 |
| GDF15 | ID1 | KCNU1 | PLA2G3 |
| INHBA | FURIN | KCNMB1 | CFLAR |
| GRAP | LTBP2 | KCNQ4 | APOE |
| FGFBP3 | F11R | LRRC55 | SCARF1 |
| CX3CL1 | ENG | KCNJ2 | CX3CL1 |
| PGF | | | |
| | | | |
| | | | |
| | | | |
| | | | |
| | | | |
| | | | |
| | | | |
| | | | |
| | | | |
| | | | |
| | | | |
| | | | |
| | | | |
| | | | |
| | | | |
| | | | |
| | | | |
| | | | |
| | | | |
| | | | |
| | | | |
| | | | |
| | | | |
| | | | |
| | | | |
| | | | |
| | | | |
| | | | |
| | | | |
| | | | |
| | | | |

| positive_regulation_of_NF-kappaB_transcription_factor_activity | ossification | cell-matrix_adhesion | blood_coagulation | sensory_perception_of_sound |
|---|---|---|---|---|
| 8 | 8 | 8 | 8 | 7 |
| GREM1 | COL1A1 | COL17A1 | COL1A1 | COL1A1 |
| PLCG2 | ECM1 | PARVG | EHD2 | GABRA5 |
| CFLAR | TMEM119 | COL13A1 | PLAU | KCNQ4 |
| TRIM38 | SPP1 | ITGA3 | F12 | MBP |
| TLR6 | BMP8A | ITGA2B | TFPI2 | GRAP |
| CX3CL1 | BMP8B | EMILIN1 | PLAUR | ICAM1 |
| TRIM22 | STC1 | HPSE | F2RL1 | OTOGL |
| ICAM1 | FSTL3 | CD34 | ENPP4 | |

| protein_localization_to_plasma_membrane | positive_regulation_of_interleukin-6_production | male_gonad_development |
|---|---|---|
| 7 | 7 | 7 |
| SLC9A3R2 | IL1A | LHCGR |
| RAB3B | CD74 | HMGCS1 |
| EHD2 | PLCG2 | CYP1B1 |
| PKP2 | F2RL1 | COL9A3 |
| F11R | MBP | SOX8 |
| NKD2 | CYBA | INHBA |
| S100A10 | TLR6 | FSTL3 |

| bicellular_tight_junction_assembly | activation_of_MAPK_activity | BMP_signaling_pathway |
|---|---|---|
| 7 | 7 | 7 |
| CLDN6 | SBK2 | FAM83G |
| CDH5 | CD74 | GDF15 |
| CLDN4 | GNG3 | FST |
| PARD6A | SHC2 | BMP8A |
| F11R | GDF15 | BMP8B |
| CLDN1 | TGFA | GDF6 |
| OCEL1 | TP73 | ENG |
| | | |
| | | |
| | | |
| | | |
| | | |
| | | |
| | | |
| | | |
| | | |
| | | |
| | | |
| | | |
| | | |
| | | |
| | | |
| | | |
| | | |
| | | |
| | | |
| | | |
| | | |
| | | |
| | | |
| | | |
| | | |
| | | |
| | | |
| | | |
| | | |
| | | |
| | | |
| | | |

| wound_healing | small_GTPase_mediated_signal_transduction | response_to_insulin |
|---|---|---|
| 6 | 6 | 6 |
| COL1A1 | RRAD | HSD11B2 |
| ARHGEF19 | RASGRF1 | FABP3 |
| TNC | HMOX1 | GAL |
| TGFA | ARHGAP18 | VGF |
| CFLAR | RAPGEF3 | PTPRN |
| ENG | RAPGEF4 | ICAM1 |

| response_to_glucocorticoid | regulation_of_blood_pressure | protein_secretion |
|---|---|---|
| 6 | 6 | 6 |
| HSD11B2 | NPPB | RAB3B |
| PTPRU | ERAP2 | STEAP3 |
| ABCA3 | NOS3 | C1QTNF5 |
| PAPPA | HMOX1 | SNCG |
| ALPL | CD34 | LTBP2 |
| AQP4 | RENBP | SCG2 |

| potassium_ion_transport | positive_regulation_of_pathway-restricted_SMAD_protein_phosphorylation | ovarian_follicle_development |
|---|---|---|
| 6 | 6 | 6 |
| KCNJ4 | GDF15 | LHCGR |
| KCNK5 | BMP8A | CEBPB |
| KCNJ6 | BMP8B | VGF |
| KCNMB1 | INHBA | KDR |
| KCNQ4 | GDF6 | INHBA |
| KCNJ2 | ENG | ICAM1 |

| negative_regulation_of_sequence-specific_DNA_binding_transcription_factor_activity | negative_regulation_of_extrinsic_apoptotic_signaling_pathway_via_death_domain_receptors | negative_regulation_of_endothelial_cell_apoptotic_process |
|---|---|---|
| 6 | 6 | 6 |
| SIGIRR | NOS3 | CDH5 |
| HES6 | TMBIM1 | TERT |
| ID1 | SERPINE1 | SERPINE1 |
| BHLHE40 | HMOX1 | KDR |
| ID3 | CFLAR | SCG2 |
| HMOX1 | ICAM1 | ICAM1 |
| | | |
| | | |
| | | |
| | | |
| | | |
| | | |
| | | |
| | | |
| | | |
| | | |
| | | |
| | | |
| | | |
| | | |
| | | |
| | | |
| | | |
| | | |
| | | |
| | | |
| | | |
| | | |
| | | |
| | | |
| | | |
| | | |
| | | |
| | | |
| | | |
| | | |
| | | |
| | | |
| | | |
| | | |

| negative_regulation_of_ERK1_and_ERK2_cascade | modulation_of_synaptic_transmission | lung_development |
|---|---|---|
| 6 | 6 | 6 |
| DUSP4 | IQSEC2 | EPAS1 |
| SPRY4 | NRG3 | CRISPLD2 |
| LIF | BDNF | ITGA3 |
| CRYBA1 | WNT3A | NOS3 |
| EMILIN1 | GRIK5 | ABCA3 |
| C1QL4 | CACNA1B | FSTL3 |
| | | |
| | | |
| | | |
| | | |
| | | |
| | | |
| | | |
| | | |
| | | |
| | | |
| | | |
| | | |
| | | |
| | | |
| | | |
| | | |
| | | |
| | | |
| | | |
| | | |
| | | |
| | | |
| | | |
| | | |
| | | |
| | | |
| | | |
| | | |
| | | |
| | | |
| | | |
| | | |
| | | |
| | | |

| hematopoietic_progenitor_cell_differentiation | female_pregnancy | cellular_response_to_organic_cyclic_compound |
|---|---|---|
| 6 | 6 | 6 |
| CEBPD | HSD11B2 | CEBPB |
| FST | CLDN4 | LGALS1 |
| KDR | PAPPA | ANKRD1 |
| INHBA | AQP4 | ALPL |
| FSTL3 | PGF | CYP1B1 |
| HERC6 | SLC19A1 | CYBA |

| cellular_response_to_mechanical_stimulus | cellular_response_to_glucose_stimulus | aortic_valve_morphogenesis |
|---|---|---|
| 6 | 6 | 6 |
| COL1A1 | LGALS1 | EFNA1 |
| ANKRD1 | GRIK5 | NOS3 |
| CYBA | AQP4 | TIE1 |
| F11R | CYBA | ELN |
| KCNJ2 | NPTX1 | SNAI1 |
| ENG | ICAM1 | EMILIN1 |

| vasodilation | vascular_endothelial_growth_factor_receptor_signaling_pathway | regulation_of_cell_adhesion |
|---|---|---|
| 5 | 5 | 5 |
| NPPB | SHC2 | JAG2 |
| NOS3 | KDR | PLAU |
| KCNMB1 | CYBA | PLAUR |
| F2RL1 | SULF1 | TNC |
| APOE | PGF | ENG |

| positive_regulation_of_protein_secretion | positive_regulation_of_protein_binding | positive_regulation_of_osteoblast_differentiation |
|---|---|---|
| 5 | 5 | 5 |
| IL1A | TERT | CEBPB |
| MYOM1 | PLXND1 | CEBPD |
| ACHE | WNT3A | TMEM119 |
| PARD6A | TRIB3 | FAM20C |
| ANKRD1 | CTHRC1 | CTHRC1 |

| **positive_regulation_of_endothelial_cell_proliferation** | **negative_regulation_of_fibroblast_growth_factor_receptor_signaling_pathway** | **liver_regeneration** |
|---|---|---|
| 5 | 5 | 5 |
| ECM1 | NGFR | CEBPB |
| KDR | CREB3L1 | CPT1A |
| CYBA | SPRY4 | HMOX1 |
| SCG2 | SHISA2 | CLDN1 |
| PGF | SULF1 | PTPN3 |
| | | |
| | | |
| | | |
| | | |
| | | |
| | | |
| | | |
| | | |
| | | |
| | | |
| | | |
| | | |
| | | |
| | | |
| | | |
| | | |
| | | |
| | | |
| | | |
| | | |
| | | |
| | | |
| | | |
| | | |
| | | |
| | | |
| | | |
| | | |
| | | |
| | | |
| | | |
| | | |
| | | |
| | | |

| defense_response | antigen_processing_and_presentation_of_exogenous_peptide_antigen_via_MHC_class_I_TAP-dependent | SMAD_protein_signal_transduction |
|---|---|---|
| 5 | 5 | 5 |
| IL32 | VAMP8_TAP2_TAP1 | GDF15 |
| CXCL6_TAP1 | CYBA | BMP8A |
| INHBA | PSMB9 | BMP8B |
| CX3CL1 | INHBA | SOX8 |
| GDF6 | | |

| skeletal_muscle_cell_differentiation | response_to_vitamin_D | response_to_progesterone |
|---|---|---|
| 4 | 4 | 4 |
| WNT3A | STC2 | CATSPER3 |
| ANKRD1 | SPP1 | CLDN4 |
| HIVEP3 | ALPL | TSPO |
| STC1 | MBP | CX3CL1 |

| regulation_of_synaptic_plasticity | regulation_of_neuronal_synaptic_plasticity | regulation_of_neurogenesis |
| --- | --- | --- |
| 4 | 4 | 4 |
| VGF | VGF | HES6 |
| RASGRF1 | RASGRF1 | BHLHE40 |
| LZTS1 | APOE | POU4F1 |
| S100B | CX3CL1 | LIF |

| regulation_of_cell_differentiation | regulation_of_angiogenesis | potassium_ion_import_across_plasma_membrane |
|---|---|---|
| 4 | 4 | 4 |
| CEBPB | EFNA1 | KCNJ4 |
| CEBPD | PLXND1 | KCNK5 |
| PLEKHB1 | HMOX1 | KCNJ6 |
| HTATIP2 | KCNJ2 | SULF1 |

| positive_regulation_of_vascular_endothelial_growth_factor_production | positive_regulation_of_receptor_internalization | positive_regulation_of_potassium_ion_transmembrane_transport |
|---|---|---|
| 4 | 4 | 4 |
| IL1A | GREM1 | KCNJ4 |
| CYP1B1 | WNT3A | KCNJ6 |
| HPSE | PLCG2 | KCNMB1 |
| PCSK9 | KCNJ2 | CTHRC1 |

| positive_regulation_of_osteoblast_proliferation | positive_regulation_of_cytokine_production_involved_in_inflammatory_response | positive_regulation_of_cell_division |
|---|---|---|
| 4 | 4 | 4 |
| TMEM119 | CD6 | IL1A |
| SOX8 | PLA2G3 | IGF2 |
| HPSE | TLR6 | TGFA |
| GPSM3 | PGF | HPSE |

| positive_regulation_of_blood_coagulation | positive_regulation_of_BMP_signaling_pathway | negative_regulation_of_smooth_muscle_cell_proliferation |
|---|---|---|
| 4 | 4 | 4 |
| F12 | CDH5 | NOS3 |
| SERPINE1 | KDR | IGFBP3 |
| ENPP4 | SULF1 | HMOX1 |
| ENG | APOE | CX3CL1 |

| negative_regulation_of_extrinsic_apoptotic_signaling_pathway_in_absence_of_ligand | negative_regulation_of_endothelial_cell_proliferation | negative_regulation_of_cell_adhesion |
|---|---|---|
| 4 | 4 | 4 |
| IL1A | APOE | PLXND1 |
| TERT | SULF1 | ARHGDIB |
| GDNF | SCG2 | TNC |
| ENG | TGFBI | NKD2 |
| | | |
| | | |
| | | |
| | | |
| | | |
| | | |
| | | |
| | | |
| | | |
| | | |
| | | |
| | | |
| | | |
| | | |
| | | |
| | | |
| | | |
| | | |
| | | |
| | | |
| | | |
| | | |
| | | |
| | | |
| | | |
| | | |
| | | |
| | | |
| | | |
| | | |
| | | |
| | | |
| | | |
| | | |
| | | |
| | | |
| | | |
| | | |

| insulin_secretion | fibrinolysis | establishment_of_endothelial_barrier |
|---|---|---|
| 4 | 4 | 4 |
| GAL | PLAU | F2RL1 |
| VGF | F12 | HPSE |
| PTPRN | SERPINE1 | RAPGEF3 |
| PLAUR | ICAM1 | SCG2 |
| | | |
| | | |
| | | |
| | | |
| | | |
| | | |
| | | |
| | | |
| | | |
| | | |
| | | |
| | | |
| | | |
| | | |
| | | |
| | | |
| | | |
| | | |
| | | |
| | | |
| | | |
| | | |
| | | |
| | | |
| | | |
| | | |
| | | |
| | | |
| | | |
| | | |
| | | |
| | | |
| | | |
| | | |
| | | |

| endothelial_cell_migration | endochondral_ossification | embryo_implantation |
|---|---|---|
| 4 | 4 | 4 |
| PLXND1 | COL1A1 | STC2 |
| NOS3 | COL13A1 | SPP1 |
| CYP1B1 | TMEM119 | LIF |
| ALPL | STC1 | STC1 |

| decidualization | cholesterol_biosynthetic_process | cell_maturation |
|---|---|---|
| 4 | 4 | 4 |
| STC2 | HMGCS1 | EPAS1 |
| SPP1 | MVD | KDR |
| LIF | DHCR7 | PLA2G3 |
| HSD17B7 | SOX8 | ENG |

| bone_development | biomineral_tissue_development | xenobiotic_transport |
|---|---|---|
| 4 | 4 | 3 |
| STC1 | ECM1 | SLC7A5 |
| NOTUM | TMEM119 | ABCA3 |
| SULF1 | SPP1 | ABCB4 |
| FAM20C | | |

| water_transport | vascular_wound_healing | surfactant_homeostasis |
|---|---|---|
| 3 | 3 | 3 |
| SLC4A11 | KDR | EPAS1 |
| AQP7 | HPSE | ABCA3 |
| AQP4 | CD34 | KDR |

| sperm-egg_recognition | response_to_organonitrogen_compound | regulation_of_receptor_activity |
|---|---|---|
| 3 | 3 | 3 |
| CATSPER3 | IL1A | PLAU |
| KCNU1 | NNMT | SERPINE1 |
| CD9 | TP73 | PCSK9 |

| **regulation_of_nitric-oxide_synthase_activity** | **regulation_of_blood_vessel_diameter** | **regulation_of_actin_cytoskeleton_reorganization** |
|---|---|---|
| 3 | 3 | 3 |
| IL1A | NPPB | ARHGDIB |
| NOS3 | NOS3 | F11R |
| CYGB | P2RY2 | RAPGEF3 |

| regulation_of_BMP_signaling_pathway | regulated_exocytosis | positive_regulation_of_wound_healing |
|---|---|---|
| 3 | 3 | 3 |
| ITGA3 | RAB11FIP1 | CLDN4 |
| FST | SYNGR3 | CLDN1 |
| FSTL3 | RAB11FIP5 | HBEGF |
| | | |
| | | |
| | | |
| | | |
| | | |
| | | |
| | | |
| | | |
| | | |
| | | |
| | | |
| | | |
| | | |
| | | |
| | | |
| | | |
| | | |
| | | |
| | | |
| | | |
| | | |
| | | |
| | | |
| | | |
| | | |
| | | |
| | | |
| | | |
| | | |
| | | |
| | | |
| | | |
| | | |
| | | |
| | | |
| | | |
| | | |
| | | |

| positive_regulation_of_viral_entry_into_host_cell | positive_regulation_of_reactive_oxygen_species_biosynthetic_process | positive_regulation_of_myoblast_fusion |
|---|---|---|
| 3 | 3 | 3 |
| CD74 | PLCG2 | EHD2 |
| LGALS1 | CYBA | GDF15 |
| TRIM38 | TLR6 | NFATC2 |

| positive_regulation_of_defense_response_to_bacterium | positive_regulation_of_branching_involved_in_ureteric_bud_morphogenesis | positive_regulation_of_actin_filament_bundle_assembly |
|---|---|---|
| 3 | 3 | 3 |
| EMILIN2 | GREM1 | ID1 |
| EMILIN1 | GDNF | SYNPO |
| CYBA | SOX8 | CX3CL1 |
| | | |
| | | |
| | | |
| | | |
| | | |
| | | |
| | | |
| | | |
| | | |
| | | |
| | | |
| | | |
| | | |
| | | |
| | | |
| | | |
| | | |
| | | |
| | | |
| | | |
| | | |
| | | |
| | | |
| | | |
| | | |
| | | |
| | | |
| | | |
| | | |
| | | |
| | | |
| | | |
| | | |
| | | |
| | | |
| | | |
| | | |

| positive_regulation_of_DNA_damage_response_signal_transduction_by_p53_class_mediator | neurotrophin_TRK_receptor_signaling_pathway | neuron_remodeling |
|---|---|---|
| 3 | 3 | 3 |
| ANKRD1 | NGFR | SCARF1 |
| PMAIP1 | BDNF | CX3CL1 |
| HIC1 | HAP1 | C1QL1 |

| negative_regulation_of_pathway-restricted_SMAD_protein_phosphorylation | negative_regulation_of_cell-substrate_adhesion | negative_regulation_of_calcium_ion_transport |
|---|---|---|
| 3 | 3 | 3 |
| GREM1 | COL1A1 | NOS3 |
| EMILIN1 | LGALS1 | STC1 |
| ENG | CX3CL1 | ICAM1 |

| negative_regulation_of_blood_coagulation | innervation | establishment_of_endothelial_intestinal_barrier |
|---|---|---|
| 3 | 3 | 3 |
| SERPINE1 | GABRA5 | F11R |
| APOE | SULF1 | CLDN1 |
| CD34 | POU4F1 | ICAM1 |

| embryonic_placenta_development | cellular_response_to_bile_acid | calcium-independent_cell-cell_adhesion_via_plasma_membrane_cell-adhesion_molecules |
|---|---|---|
| 3 | 3 | 3 |
| CEBPB | KCNMB1 | CLDN6 |
| EPAS1 | ABCB4 | CLDN4 |
| IGF2 | NR1H4 | CLDN1 |
| | | |
| | | |
| | | |
| | | |
| | | |
| | | |
| | | |
| | | |
| | | |
| | | |
| | | |
| | | |
| | | |
| | | |
| | | |
| | | |
| | | |
| | | |
| | | |
| | | |
| | | |
| | | |
| | | |
| | | |
| | | |
| | | |
| | | |
| | | |
| | | |
| | | |
| | | |
| | | |
| | | |
| | | |
| | | |
| | | |
| | | |
| | | |

| behavioral_response_to_pain | antigen_processing_and_presentation_of_endogenous_peptide_antigen_via_MHC_class_I | adrenal_gland_development |
|---|---|---|
| 3 | 3 | 3 |
| SCN9A | ERAP2_TAP2_TAP1 | TSPO |
| TSPO | CYP1B1 | GDNF |
| VWA1 | FSTL3 | |

| ureteric_bud_formation | smooth_muscle_hyperplasia | response_to_hydrostatic_pressure |
|---|---|---|
| 2 | 2 | 2 |
| GREM1 | NOS3 | COL18A1 |
| HMOX1 | KRT8 | ICAM1 |

| response_to_gonadotropin | regulation_of_organelle_transport_along_microtubule | regulation_of_cysteine-type_endopeptidase_activity |
|---|---|---|
| 2 | 2 | 2 |
| ITGA3 | HAP1 | PSMB9 |
| NEFH | BIRC3 | F11R |
| | | |
| | | |
| | | |
| | | |
| | | |
| | | |
| | | |
| | | |
| | | |
| | | |
| | | |
| | | |
| | | |
| | | |
| | | |
| | | |
| | | |
| | | |
| | | |
| | | |
| | | |
| | | |
| | | |
| | | |
| | | |
| | | |
| | | |
| | | |
| | | |
| | | |
| | | |
| | | |
| | | |
| | | |
| | | |
| | | |
| | | |

| protein_localization_to_bicellular_tight_junction | postsynaptic_membrane_organization | positive_regulation_of_phospholipid_efflux |
|---|---|---|
| 2 | 2 | 2 |
| CDH5 | CHRNB1 | ABCA3 |
| GDNF | APOE | LIF |
| | | |
| | | |
| | | |
| | | |
| | | |
| | | |
| | | |
| | | |
| | | |
| | | |
| | | |
| | | |
| | | |
| | | |
| | | |
| | | |
| | | |
| | | |
| | | |
| | | |
| | | |
| | | |
| | | |
| | | |
| | | |
| | | |
| | | |
| | | |
| | | |
| | | |
| | | |
| | | |
| | | |
| | | |
| | | |
| | | |
| | | |

| positive_regulation_of_mesenchymal_to_epithelial_transition_involved_in_metanephros_morphogenesis | positive_regulation_of_low-density_lipoprotein_particle_receptor_catabolic_process | positive_regulation_of_histamine_secretion_by_mast_cell |
|---|---|---|
| 2 | 2 | 2 |
| GDNF | PCSK9 | VAMP8 |
| APOE | PLA2G3 | SPINT1 |

| positive_regulation_of_glial_cell_differentiation | platelet_activating_factor_biosynthetic_process | negative_regulation_of_hair_follicle_development | negative_regulation_of_adiponectin_secretion | mature_conventional_dendritic_cell_differentiation |
|---|---|---|---|---|
| 2 | 2 | 2 | 2 | 2 |
| NKX6-2 | LPCAT2 | NGFR | RAB11FIP1 | F2RL1 |
| PLA2G4C | INHBA | RAB11FIP5 | CCR7 | NEFH |

| intermediate_filament_bundle_assembly | gamma-delta_T_cell_activation | extracellular_matrix_constituent_secretion | cytosol_to_ER_transport | chemoattraction_of_axon |
|---|---|---|---|---|
| 2 | 2 | 2 | 2 | 2 |
| PKP2 | ITK | CREB3L1 | TAP2_TAP1 | ROBO3 |
| MICB | ENG | GDNF | | |

**Biologicial pathways associated with the genes downregulated upon LSD1 inhibition**

| positive_regulation_of_transcription_from_RNA_polymerase_II_promoter | cell_adhesion | nervous_system_development | multicellular_organism_development |
|---|---|---|---|
| 11 | 11 | 7 | 7 |
| SOX3 | SEMA5A | SEMA5A | CENPE |
| TOX2 | VCAM1 | EFNB3 | PAX6 |
| NR2F1 | KIRREL2 | NR2F1 | EBF4 |
| PAX6 | LAMA4 | DCX | DLL1 |
| GLI1 | PCDH8 | ST8SIA4 | FGFR2 |
| DLL1 | CNTN2 | MDGA1 | EPHA3 |
| KLF15 | NCAM1 | PCDH18 | GADD45G |
| FGFR2 | PCDH19 | | |
| HES5 | PCDH18 | | |
| RGMA | EPHA3 | | |
| FGF10 | HES5 | | |

| axon_guidance | cell-cell_signaling | regulation_of_smoothened_signaling_pathway | positive_regulation_of_neuron_projection_development | negative_regulation_of_neuron_differentiation |
|---|---|---|---|---|
| 7 | 6 | 5 | 5 | 5 |
| SEMA5A | SEMA5A | PTCH1 | CNR1 | SOX3 |
| EFNB3 | EFNB3 | GAS1 | STMN2 | CNTN2 |
| CNTN2 | PCDH8 | GLI1 | NDNF | PAX6 |
| PAX6 | TRHDE | FGFR2 | EPHA3 | DLL1 |
| NCAM1 | EFNA4 | FGF10 | RGMA | HES5 |
| EFNA4 | FGFR2 | | | |
| EPHA3 | | | | |
| | | | | |
| | | | | |
| | | | | |
| | | | | |

| brain_development | MAPK_cascade | smoothened_signaling_pathway | response_to_lipopolysaccharide | response_to_ethanol | positive_regulation_of_canonical_Wnt_signaling_pathway |
|---|---|---|---|---|---|
| 5 | 5 | 4 | 4 | 4 | 4 |
| PTCH1 | NCAM1 | PTCH1 | VCAM1 | VCAM1 | SEMA5A |
| PCDH19 | MAPK8IP2 | PAX6 | CNR1 | CNR1 | FRAT1 |
| MDGA1 | FGFR2 | GLI1 | FGFR2 | OPRK1 | FGFR2 |
| PCDH18 | MAP2K6 | HES5 | FGF10 | FGFR2 | FGF10 |
| HES5 | FGF10 | | | | |
| | | | | | |
| | | | | | |
| | | | | | |
| | | | | | |
| | | | | | |
| | | | | | |

| pituitary_gland_development | neuron_migration | homophilic_cell_adhesion_via_plasma_membrane_adhesion_molecules | extracellular_matrix_organization | central_nervous_system_development | animal_organ_morphogenesis |
|---|---|---|---|---|---|
| 4 | 4 | 4 | 4 | 4 | 4 |
| SOX3 | DCX | PCDH8 | VCAM1 | SOX3 | PTCH1 |
| PAX6 | NDNF | CNTN2 | LAMA4 | RPS6KA6 | PAX6 |
| GLI1 | PAX6 | PCDH19 | NDNF | DCX | FGFR2 |
| FGF10 | MDGA1 | PCDH18 | IMPG2 | PAX6 | FGF10 |
| | | | | | |
| | | | | | |
| | | | | | |
| | | | | | |
| | | | | | |
| | | | | | |
| | | | | | |

| transmembrane_receptor_protein_tyrosine_kinase_signaling_pathway | retina_development_in_camera-type_eye | regulation_of_cell_migration | potassium_ion_transmembrane_transport | positive_regulation_of_Notch_signaling_pathway |
|---|---|---|---|---|
| 3 | 3 | 3 | 3 | 3 |
| CLNK | DCX | LAMA4 | KCNH2 | DLL1 |
| FGFR2 | PAX6 | PAX6 | KCNH8 | HES5 |
| EPHA3 | DLL1 | FGF10 | KCNC4 | FGF10 |
| | | | | |
| | | | | |
| | | | | |
| | | | | |
| | | | | |
| | | | | |
| | | | | |
| | | | | |

| peptidyl-tyrosine_phosphorylation | locomotory_behavior | lacrimal_gland_development | glucose_homeostasis |
|---|---|---|---|
| 3 | 3 | 3 | 3 |
| FGFR2 | LRRTM1 | PAX6 | CNR1 |
| EPHA3 | NPY2R | FGFR2 | PTCH1 |
| MAP2K6 | OPRK1 | FGF10 | PAX6 |
| | | | |
| | | | |
| | | | |
| | | | |
| | | | |
| | | | |
| | | | |
| | | | |

| ephrin_receptor_signaling_pathway | cellular_response_to_retinoic_acid | cell_fate_determination | canonical_Wnt_signaling_pathway |
|---|---|---|---|
| 3 | 3 | 3 | 3 |
| EFNB3 | FZD7 | PTCH1 | FRAT1 |
| EFNA4 | FGFR2 | PAX6 | FZD7 |
| EPHA3 | EPHA3 | DLL1 | GLI1 |
| | | | |
| | | | |
| | | | |
| | | | |
| | | | |
| | | | |
| | | | |
| | | | |

| axonal_fasciculation | Notch_signaling_pathway | sialylation | secretion | retina_morphogenesis_in_camera-type_eye |
|---|---|---|---|---|
| 3 | 3 | 2 | 2 | 2 |
| SEMA5A | DTX4 | ST6GAL2 | CA2 | DLL1 |
| CNR1 | DLL1 | ST8SIA4 | NPY2R | IMPG2 |
| CNTN2 | HES5 | | | |
| | | | | |
| | | | | |
| | | | | |
| | | | | |
| | | | | |
| | | | | |
| | | | | |
| | | | | |

| regulation_of_saliva_secretion | regulation_of_osteoblast_differentiation | pyramidal_neuron_development | proximal_distal_pattern_formation |
|---|---|---|---|
| 2 | 2 | 2 | 2 |
| OPRK1 | GLI1 | DCX | GLI1 |
| FGF10 | FGFR2 | FGFR2 | DLL1 |
| | | | |
| | | | |
| | | | |
| | | | |
| | | | |
| | | | |
| | | | |
| | | | |
| | | | |

| prostate_gland_development | positive_regulation_of_epithelial_cell_proliferation_involved_in_wound_healing | positive_regulation_of_dopamine_secretion | otic_vesicle_formation |
|---|---|---|---|
| 2 | 2 | 2 | 2 |
| PTCH1 | FZD7 | NPY2R | FGFR2 |
| GLI1 | FGF10 | OPRK1 | FGF10 |
| | | | |
| | | | |
| | | | |
| | | | |
| | | | |
| | | | |
| | | | |
| | | | |
| | | | |

| organ_growth | oligosaccharide_metabolic_process | nitric_oxide_mediated_signal_transduction | neuronal_stem_cell_population_maintenance |
|---|---|---|---|
| 2 | 2 | 2 | 2 |
| DLL1 | ST6GAL2 | NPY2R | DLL1 |
| FGFR2 | ST8SIA4 | NDNF | HES5 |
| | | | |
| | | | |
| | | | |
| | | | |
| | | | |
| | | | |
| | | | |
| | | | |
| | | | |

| negative_regulation_of_endocytosis | negative_regulation_of_cardiac_muscle_cell_differentiation | negative_regulation_of_auditory_receptor_cell_differentiation | morphogenesis_of_embryonic_epithelium |
|---|---|---|---|
| 2 | 2 | 2 | 2 |
| ABCA7 | FZD7 | DLL1 | PCDH8 |
| EPHA3 | DLL1 | HES5 | FGFR2 |
| | | | |
| | | | |
| | | | |
| | | | |
| | | | |
| | | | |
| | | | |
| | | | |
| | | | |

| mesenchymal_cell_differentiation_involved_in_lung_development | mammary_gland_bud_formation | limb_bud_formation | fibroblast_growth_factor_receptor_signaling_pathway_involved_in_mammary_gland_specification |
|---|---|---|---|
| 2 | 2 | 2 | 2 |
| FGFR2 | FGFR2 | FGFR2 | FGFR2 |
| FGF10 | FGF10 | FGF10 | FGF10 |
| | | | |
| | | | |
| | | | |
| | | | |
| | | | |
| | | | |
| | | | |
| | | | |
| | | | |

| epithelial_cell_proliferation_involved_in_salivary_gland_morphogenesis | embryonic_digestive_tract_morphogenesis | commissural_neuron_axon_guidance | central_nervous_system_myelination |
|---|---|---|---|
| 2 | 2 | 2 | 2 |
| FGFR2 | FGFR2 | PTCH1 | CNTN2 |
| FGF10 | FGF10 | NCAM1 | HES5 |
| | | | |
| | | | |
| | | | |
| | | | |
| | | | |
| | | | |
| | | | |
| | | | |
| | | | |

| bud_elongation_involved_in_lung_branching | branch_elongation_involved_in_salivary_gland_morphogenesis | astrocyte_differentiation |
|---|---|---|
| 2 | 2 | 2 |
| FGFR2 | FGFR2 | PAX6 |
| FGF10 | FGF10 | HES5 |
| | | |
| | | |
| | | |
| | | |
| | | |
| | | |
| | | |
| | | |
| | | |

| 187 genes upregulated upon LSD1 inhibiton and expressed in human cortical primordium (Nowakowski et al., 2017) | Expression in different cell type clusters in human cortical primordium |
|---|---|
| ABCA3 | Astrocyte |
| ABCA4 | Choroid |
| ABCB4 | Endothelial |
| ADAM12 | Choroid,Mural |
| ADAM33 | tRG,Mural |
| ADAP2 | Microglia,Mural |
| ANKRD45 | Choroid |
| ANO4 | EN-V1-3,EN-PFC3,EN-PFC2,EN-V1-1,EN-PFC1 |
| AQP4 | Astrocyte,oRG |
| ARHGAP18 | RG-div2,oRG,Choroid,Endothelial |
| ARHGDIB | Microglia,Mural,Endothelial |
| ASS1 | |
| BEST3 | MGE-IPC3,MGE-IPC2,MGE-div,nIN5,nIN4,nIN1 |
| BEX2 | MGE-IPC3,MGE-IPC2,MGE-IPC1,MGE-div,MGE-RG2,RG-div1,tRG,Microglia,Glyc,Mural,Endothelial,nIN4,nIN2,nIN1,IN-CTX-MGE1,IN-CTX-CGE1,IN-STR,EN-V1-3,nEN-early1,EN-PFC2,EN-V1-2,EN-V1-1,EN-PFC1 |
| BHLHE40 | oRG,Microglia,Mural,Endothelial |
| BMP8B | OPC |
| BNC2 | |
| BRI3 | Microglia,Endothelial |
| CACNA1B | EN-PFC2,EN-V1-1,EN-PFC1 |
| CD34 | Endothelial |
| CD74 | Microglia |
| CD97 | Microglia |
| CDH5 | Endothelial |
| CEBPB | Microglia,Mural,Endothelial |
| CEBPD | Microglia,Mural,Endothelial |
| CELF5 | EN-V1-3,EN-PFC3,nEN-early1,EN-PFC2,EN-V1-2,EN-V1-1,EN-PFC1 |
| CFLAR | OPC,Microglia,Endothelial |
| CHRNB1 | oRG |
| CLDN1 | Choroid |
| CLIC6 | Choroid |
| COL12A1 | |
| COL18A1 | Choroid,Mural |
| COL1A1 | Mural |

| | |
|---|---|
| COL25A1 | EN-PFC1 |
| COL6A2 | |
| COL6A3 | |
| COL9A2 | OPC,Choroid |
| COL9A3 | |
| CPEB2 | OPC,Endothelial,IPC-div1,IPC-nEN1,EN-PFC1 |
| CPPED1 | Mural |
| CPT1A | RG-div2,Astrocyte,OPC,RG-div1,vRG,oRG,Choroid,Mural,Endothelial |
| CREB3L1 | |
| CRISPLD2 | |
| CSRNP1 | tRG,Microglia,Mural,Endothelial |
| CTHRC1 | Endothelial |
| CX3CL1 | Endothelial |
| CYP1B1 | |
| DEPDC7 | |
| DHCR7 | RG-div2,oRG,Glyc,nEN-early1,EN-PFC2 |
| DNAH5 | Choroid |
| DNAJC6 | Glyc,EN-V1-3,nEN-early1,EN-PFC2,EN-V1-2,EN-PFC1 |
| DUSP4 | EN-PFC3,EN-PFC2 |
| ECM1 | Mural,Endothelial |
| EFNA1 | Endothelial |
| EHD2 | Mural,Endothelial |
| ELN | Choroid |
| ELTD1 | Endothelial |
| EMP1 | Endothelial |
| ENG | Mural,Endothelial |
| ENO2 | MGE-RG2,MGE-RG1,RG-early,Microglia,Glyc,nIN4,nIN2,IPC-div2,IPC-nEN2,IN-CTX-CGE2,EN-V1-3,nEN-early1,EN-PFC2,EN-V1-1,nEN-early2,EN-PFC1 |
| EPAS1 | Mural,Endothelial |
| ERAP2 | Endothelial |
| F11R | Endothelial |
| FABP3 | Glyc,EN-V1-3,EN-PFC2 |
| FAM129A | Endothelial |
| FAM179A | Choroid |
| FAM189A2 | oRG,Endothelial |
| FAM20C | |
| FGFBP3 | NEN-early1,MGE-RG1,RG-div2,Astrocyte,OPC,RG-div1,vRG,tRG,oRG |

| | |
|---|---|
| FST | |
| GABRA5 | EN-PFC1 |
| HAPLN3 | Endothelial |
| HBEGF | tRG,oRG,Microglia,Mural,Endothelial |
| HERC6 | oRG |
| HIVEP3 | IN-CTX-MGE1,EN-V1-3,EN-V1-2,nEN-early2 |
| HMGCS1 | MGE-IPC2,MGE-IPC1,MGE-RG1,RG-div2,Astrocyte,RG-div1,vRG,tRG,oRG,Choroid,Glyc,Endothelial,nIN5,nIN3,nIN2,nIN1,IPC-div2,IN-CTX-MGE2,IN-CTX-MGE1,IN-CTX-CGE2,IN-CTX-CGE1,IN-STR,EN-V1-3,EN-PFC3,nEN-early1,EN-PFC2,EN-V1-2,nEN-late,EN-PFC1 |
| HMOX1 | Microglia |
| HOOK1 | Choroid,EN-PFC1 |
| HSD17B7 | RG-div2,RG-div1,tRG,oRG,nEN-early1 |
| HSD17B7P2 | RG-div2,Astrocyte,OPC,tRG,oRG,EN-PFC2 |
| HSPA12B | Endothelial |
| ICAM1 | Microglia,Endothelial |
| ID1 | Astrocyte,Choroid,Endothelial |
| IGF2 | Choroid,Mural |
| IKZF1 | Microglia |
| IL1A | Microglia |
| INHBA | EN-V1-3 |
| INPP5D | Microglia,Endothelial |
| KCNJ2 | MGE-IPC2,Endothelial,IN-CTX-CGE2 |
| KCNJ6 | EN-PFC3,EN-PFC2,EN-V1-1,EN-PFC1 |
| KCNMB1 | Mural |
| KDR | Endothelial |
| LAMC2 | |
| LGALS3BP | MGE-RG1,RG-div2,Astrocyte,RG-div1,tRG,oRG,Mural |
| LPCAT2 | Microglia |
| LPPR5 | EN-V1-3,EN-PFC2,EN-V1-2,EN-V1-1,EN-PFC1 |
| LRRC55 | Endothelial,nEN-early1 |
| LRRK1 | Microglia |
| LUM | Mural,Endothelial |
| MAOA | Endothelial |
| MBP | OPC,Microglia |
| MFAP3L | |
| MIR137HG | EN-V1-3,EN-PFC2,EN-PFC1 |
| MVD | RG-div2,tRG,oRG,EN-V1-3,EN-PFC2,EN-V1-2 |

| | |
|---|---|
| NFATC2 | tRG,Microglia,Endothelial |
| NKD2 | Endothelial |
| NLRC5 | Endothelial |
| NPL | Microglia |
| NPTX1 | nEN-early1,EN-V1-1 |
| NRG3 | EN-V1-3,EN-PFC1 |
| NT5E | OPC |
| NUDT14 | Endothelial |
| OCIAD2 | MGE-div,MGE-RG2,MGE-RG1,OPC,RG-div1,vRG,oRG,RG-early,Microglia,Mural,IN-CTX-MGE2,IN-CTX-CGE2,IN-CTX-CGE1,EN-V1-3,nEN-early1,EN-V1-2,nEN-late,nEN-early2 |
| OLFML3 | Microglia,Mural,Endothelial |
| PAPPA | EN-V1-1,EN-PFC1 |
| PARM1 | Choroid |
| PARVG | Microglia |
| PCBP3 | Mural |
| PCSK9 | |
| PEAR1 | Mural,Endothelial |
| PGF | Endothelial |
| PIK3R5 | Microglia |
| PKP2 | Choroid |
| PLA2G4C | Endothelial |
| PLAU | Microglia |
| PLAUR | Microglia,Endothelial |
| PLCB2 | Microglia |
| PLEKHA4 | Choroid |
| PLP2 | MGE-div,RG-div1,Endothelial |
| PLXND1 | Endothelial |
| PMAIP1 | MGE-div,Microglia,Endothelial |
| PRUNE2 | RG-div1,oRG,Glyc |
| PTPRN | Glyc |
| RAB11FIP1 | Choroid |
| RAB11FIP4 | EN-V1-3,EN-V1-1,EN-PFC1 |
| RAB11FIP5 | Endothelial |
| RAB3B | Choroid,Glyc |
| RAPGEF3 | |
| RAPGEF4 | OPC,Mural,Endothelial |
| RHBDF2 | Microglia |
| ROBO3 | Astrocyte |
| S100A10 | Choroid,Endothelial |
| S100A11 | Microglia,Mural,Endothelial |
| S100A4 | |
| S100B | Astrocyte,OPC,oRG |
| SCARF1 | Endothelial |

| | |
|---|---|
| SCG2 | Glyc,EN-V1-3,EN-PFC3,EN-PFC2 |
| SCN9A | EN-V1-3 |
| SERPINB8 | |
| SERPINB9 | Microglia,Mural,Endothelial |
| SERPINE1 | Mural,Endothelial |
| SHC2 | Endothelial |
| SHISA2 | MGE-IPC3,MGE-IPC2,MGE-IPC1,MGE-div,MGE-RG2,MGE-RG1,RG-div2,Astrocyte,OPC,RG-div1,vRG,tRG,oRG,RG-early,Microglia,Mural,Endothelial,nIN5,nIN4,nIN2,nIN1,IPC-div1,IN-CTX-MGE2,IN-CTX-MGE1,IN-CTX-CGE2,IN-CTX-CGE1,IN-STR,EN-PFC3,nEN-early1,EN-V1-1,nEN-late,EN-PFC1 |
| SLC15A3 | Microglia |
| SLC16A6 | Choroid |
| SLC2A14 | Mural,Endothelial |
| SLC7A5 | Endothelial |
| SLFN13 | |
| SNAI1 | Endothelial |
| SNCG | Endothelial |
| SOX8 | Astrocyte |
| SP140L | Mural |
| SPINT2 | Choroid,Glyc,EN-V1-3,EN-V1-1,EN-PFC1 |
| SPP1 | Microglia |
| SPRY4 | OPC,Mural,Endothelial |
| SQSTM1 | oRG,Microglia,Glyc,Endothelial |
| SRRM3 | EN-V1-3,EN-PFC1 |
| ST3GAL5 | Astrocyte,OPC,oRG,Glyc,Mural,EN-V1-1,EN-PFC1 |
| STC1 | Choroid,Glyc,Endothelial,EN-V1-2 |
| SULF1 | Choroid |
| SYNGR3 | EN-V1-3 |
| SYNM | Mural,Endothelial |
| SYT14 | MGE-IPC3,MGE-IPC1,MGE-div,MGE-RG2,MGE-RG1,Astrocyte,OPC,RG-div1,vRG,tRG,Microglia,Mural,Endothelial,IPC-div2,IPC-nEN2,IN-CTX-CGE2,EN-V1-3,EN-PFC3,nEN-early1,EN-PFC2,EN-V1-2,EN-V1-1,EN-PFC1 |
| TAGLN2 | RG-div2,RG-div1,vRG,tRG,oRG,Endothelial,IPC-div1 |
| TGFA | |

| | |
|---|---|
| TGFBI | |
| TIE1 | Endothelial |
| TMBIM1 | oRG,Microglia |
| TMEM119 | |
| TMEM130 | nEN-early1,EN-PFC1 |
| TRIM38 | Microglia,Mural,Endothelial |
| TRIM47 | Astrocyte |
| TSPO | RG-div2,tRG,EN-V1-3 |
| UNC13A | Glyc,EN-V1-3,EN-PFC2 |
| VAMP8 | Microglia,Mural,Endothelial |
| VWA1 | Endothelial |
| WSCD1 | RG-div2,Astrocyte,OPC,RG-div1,tRG,oRG,IPC-div2 |
| | |

| Cluster Name | Cluster Interpretation |
|---|---|
| Astrocyte | Astocyte |
| IN-CTX-CGE1 | CGE/LGE-derived inhibitory neurons |
| IN-CTX-CGE2 | CGE/LGE-derived inhibitory neurons |
| Choroid | Choroid |
| IPC-div1 | Dividing Intermediate Progenitor Cells RG-like |
| MGE-div | dividing MGE Progenitors |
| RG-div1 | Dividing Radial Glia (G2/M-phase) |
| RG-div2 | Dividing Radial Glia (S-phase) |
| EN-PFC2 | Early and Late Born Excitatory Neuron PFC |
| EN-PFC3 | Early and Late Born Excitatory Neuron PFC |
| EN-V1-2 | Early and Late Born Excitatory Neuron V1 |
| EN-PFC1 | Early Born Deep Layer/subplate Excitatory Neuron PFC |
| EN-V1-1 | Early Born Deep Layer/subplate Excitatory Neuron V1 |
| RG-early | earlyRG |
| Endothelial | Endothelial |
| EN-V1-3 | Excitatory Neuron V1 - late born |
| Glyc | Glycolysis |
| IPC-nEN1 | Intermediate Progenitor Cells EN-like |
| IPC-nEN2 | Intermediate Progenitor Cells EN-like |
| IPC-nEN3 | Intermediate Progenitor Cells EN-like |
| IPC-div2 | Intermediate Progenitor Cells RG-like |
| nIN1 | MGE newborn neurons |
| nIN2 | MGE newborn neurons |
| nIN3 | MGE newborn neurons |
| nIN4 | MGE newborn neurons |
| nIN5 | MGE newborn neurons |
| MGE-IPC1 | MGE Progenitors |
| MGE-IPC2 | MGE Progenitors |
| MGE-IPC3 | MGE Progenitors |
| MGE-RG1 | MGE Radial Glia 1 |
| MGE-RG2 | MGE Radial Glia 2 |
| IN-CTX-MGE2 | MGE-derived Ctx inhibitory neuron, Cortical Plate-enriched |
| IN-CTX-MGE1 | MGE-derived Ctx inhibitory neuron, Germinal Zone Enriched |

| | |
|---|---|
| Microglia | Micrgolia |
| Mural | Mural/Pericyte |
| nEN-early2 | Newborn Excitatory Neuron - early born |
| nEN-early1 | Newborn Excitatory Neuron - early born |
| nEN-late | Newborn Excitatory Neuron - late born |
| OPC | Oligodendrocyte progenitor cell |
| oRG | Outer Radial Glia |
| IN-STR | Striatal neurons |
| tRG | Truncated Radial Glia |
| vRG | Ventricular Radial Glia |
