# Supplementary material for: LSD1 Regulates Neurogenesis in Human Neural Stem Cells Through the Repression of Human-Enriched Extracellular Matrix and Cell Adhesion Genes
Source: Stem Cells. 2023 Dec 28;42(2):128–45. doi: 10.1093/stmcls/sxad088 (PMC10852026; doi:10.1093/stmcls/sxad088)
Supplement: Supplementary file 1 [file sxad088_suppl_supplementary_figures.docx]

**Supplementary figure 1**:(A) hNSCs were differentiated into neurons and astrocytes for 14 and 21 days in vitro. Differentiating neurons express deep-layer neuronal markers TBR1, TLE4 and CTIP2 at DIV14 and superficial markers SATB2 and BRN2 at DIV21. All the neurons were co-stained with β-III tubulin or DCX. Differentiating astrocytes express GFAP and S100β (n=3) Scale bar: 50µM. GO ontology enrichment analysis for all LSD1 peaks (B) and peaks around TSS (transcription start site) (C) shows a significant association with biological pathway terms related to signal transduction, nervous system development and neural cellular processes. (D) MEME analysis showing the candidate motifs enriched around transcription start site (TSS).

**Supplementary figure 2:** (A) Schematic of the RNA-seq experimental paradigm - hNSCs were treated with vehicle or 10µM GSK-LSD1 for 48hours and RNA collected for analysis. (B) qRT-PCR validation of HEYL transcript levels in Vehicle or treated cells (n= 3). (C) Western blot shows global H3K4me2 and H3K27ac levels (normalised to Lamin B1) in GSK-LSD1 treated hNSCs as compared to vehicle (left). Densitometric quantification of relative expression levels of H3K4me2 and H3K27ac (right) (n=3). (D) Heat map showing genes differentially expressed in hNSCs on treatment with 10µM GSK-LSD1 compared to vehicle. Out of 584 differentially expressed genes, 483 genes were upregulated and 101 genes were downregulated upon GSK-LSD1 treatment (n=3) (E) Mean average (MA) plot depicting the relationship between log fold change and average abundance of each gene (counts per million). Significantly differentially expressed genes are highlighted in blue and red (FDR <0.05). (F) Distribution of LSD1 bound peaks in the different genomic regions for up and downregulated genes upon LSD1 inhibition.

 

**Supplementary figure 3:** (A) Control GFP, HES6, and TLE1 overexpressing hNSCs were differentiated for 7 DIV and EdU labelling was done for 2 hours before fixation. Representative images for each condition showing EdU, and proliferation marker SOX2 and GFP. White arrows indicate SOX2+ EdU+GFP- cells and arrowheads indicate cells that are SOX2+ EdU+GFP+ or SOX2+ GFP+ (top). Bar graphs depicting percentage of SOX2+EdU+ (normalised to DAPI), SOX2+EdU+GFP+ (normalised to GFP) and SOX2+GFP+ (normalised to GFP) upon GFP expression or HES6 or TLE1 overexpression (n=4) (B) hNSCs expressing GFP, HES6 and TLE1 were treated with propidium iodide (PI) for 30 minutes before fixation. Representative images showing PI and GFP staining (left). Bar graph depicting percentage of PI+ cells/DAPI (n=3) (right). 

Nuclei were counterstained with DAPI (blue). Scale bar 50µM. Error bars represent SEM, *p<0.05, **p<0.01.

 

**Supplementary figure 4:** (A) hNSCs ectopically expressing GFP, LGALS3BP, SERPINE1 and NOTUM were differentiated for 7 days and EdU pulsing was done for 2 hours before fixation. Representative images showing EdU, SOX2 and GFP expression. White arrows indicate SOX2+ EdU+GFP- cells and arrowheads indicate cells that are SOX2+ EdU+GFP+ or SOX2+ GFP+ (left). Quantification of percentage of SOX2+EdU+ (normalised to DAPI), SOX2+EdU+GFP+ cells (normalised to GFP) and SOX2+GFP+ (normalised to GFP) cells upon LGALS3BP, SERPINE1 and NOTUM overexpression vs GFP (right) (n=3 or 4)

(B) hNSCs nucleofected with GFP, LGALS3BP, SERPINE1 and NOTUM were differentiated for 7 DIV. Cells were treated with PI for 30 minutes before fixing. Confocal images showing the PI and GFP staining in each overexpression condition (left). Bar graph depicting percentage of PI+ cells upon GFP, LGALS3BP, SERPINE1 and NOTUM overexpression. Nuclei were counterstained with DAPI (blue). Scale bar 50µM. Error bars represent SEM, *p<0.05, **p<0.01.

**Supplementary figure 5:** (A) Heat map showing the peak density for H3K4me1/2 and H3K9me1/2 around ±2.5kb of the gene centre. Shown above are the profile plots for the corresponding heat maps. (B) H3K4me2 methylation marks at the promoter/TSS of genes associated with ECM and Notch pathway are compared between mouse and human forebrain. IGV tracks showing the H3K4me2 levels over promoters of *SERPINE1, LGALS3BP* and *TLE1* in mouse genome at E14 (top) and human at 8 weeks post conception (bottom) (B).
